# Lanthanide-Centered Polyoxoalkoxide Complexes Provide a Platform for Systematic Room- and Variable-Temperature Nuclear Magnetic Resonance Studies

**DOI:** 10.1021/acs.inorgchem.6c01439

**Published:** 2026-05-21

**Authors:** Dominic Shiels, Nadeeshan Gunarathna, William W. Brennessel, Ellen M. Matson

**Affiliations:** Department of Chemistry, 6927 University of Rochester, Rochester, New York 14627, United States

## Abstract

A single, high-yielding approach was employed to synthesize the complete series of lanthanide (Ln) containing polyoxoalkoxide sandwich-type complexes (TBA)_3_[M­{Mo_5_O_13_(OMe)_4_NO}_2_] (M = La–Lu, except *Pm*). The complexes were characterized by solution-state ^1^H/^17^O NMR spectroscopy, infrared spectroscopy, cyclic voltammetry, and electronic absorption spectroscopy, while the new derivatives (M = Tm, Yb, Lu, and Y) were also characterized using single crystal X-ray diffraction experiments. Compared to similar [Ln­(W_5_O_18_)_2_]^9–^ or [Ln­(PW_11_O_39_)_2_]^11–^ series, the presence of large noncoordinating tetrabutylammonium cations makes this series unique in that all (TBA)_3_[M­{Mo_5_O_13_(OMe)_4_NO}_2_] complexes are isostructural in the solid-state, with no changes in crystal packing or polymorph across the series. Solution-state NMR investigations reveal large Ln induced shifts (ca. 100–2000 ppm) for ^17^O nuclei directly bound to the Ln center. Variable temperature ^17^O NMR spectroscopy reveals that the chemical shift these oxygen nuclei show significant temperature dependence, opening the door to ^17^O NMR thermometry with Ln containing polyoxometalates.

## Graphical abstract

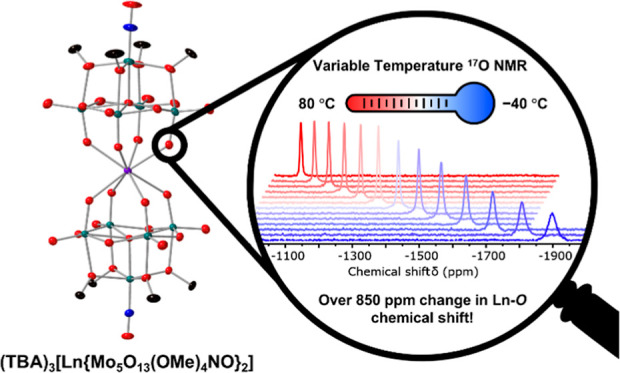

## Introduction

Polyoxometalates (POMs) are a broad class of anionic, molecular metal oxide assemblies typically formed from Mo^VI^, W^VI^, or V^V^ precursors.^1–10^ The accessible structural landscape spans a range of geometries which include fully saturated and lacunary Keggin ([XM_12_O_40_]^*n*−^), Wells-Dawson ([X_2_M_18_O_62_]^*n*−^), Lindqvist ([M_6_O_19_]^*n*−^), Preyssler [XP_5_W_30_O_110_]^*n*−^, and Anderson-Evans ([XM_6_O_24_]^*n*−^) frameworks (X = heteroatom). These disparate structures present distinct coordination geometries, charge densities, and electrochemical profiles that modulate the properties of the assembly.^11–18^ The multidentate, all-oxygen donor surfaces of POMs have translated to their use as uniquely versatile inorganic ligands for f-element coordination chemistry.^1–3,19–28^ Since Peacock and Weakley first reported the archetypical [Ln­(W_5_O_18_)_2_]^9–^ sandwich complex in 1971,^29^ the Ln containing POM (LnPOM) structural landscape has expanded. The monolacunary Keggin ([XW_11_O_39_]^*n*‑^) and Wells-Dawson [X_2_W_17_O_61_]^*n*−^ fragments assemble in both 1:1 and 2:1 complexes with Ln^3+^ ions.^30–33^ In contrast, Preyssler-type POMs (e.g., [LnP_5_W_30_O_110_]^12−^) encapsulate Ln^3+^ ions (see Figure 1 for some example structures).^34–37^ In each of these families, the oxygen-donor POM ligands impose a rigid coordination environment that stabilizes single-ion magnetic anisotropy and slow magnetization relaxation.^38–44^ This has established LnPOM complexes as competitive single-molecule magnets relevant to molecular spintronics and quantum information science, motivating extensive research into the elucidation of structure-property relationships that control these phenomena.^45–48^

**Figure 1:**
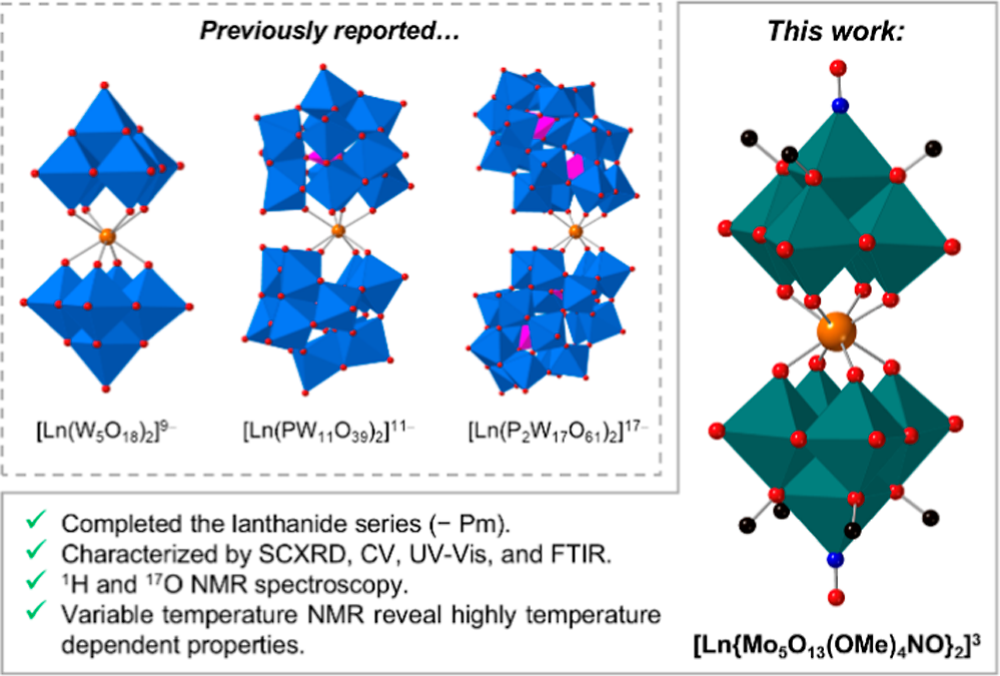
An overview of some of the series of LnPOM complexes previously reported in the literature. These are compared to the general structure of the **Ln­(Mo**_**5**_ **)**_**2**_ complexes discussed in this work, along with a description of the characterization data that was acquired.

Separately, Ln complexes are well studied in the context of nuclear magnetic resonance (NMR) spectroscopy and magnetic resonance imaging (MRI), primarily for biochemical and medical applications.^49–58^ In this setting, the presence of a paramagnetic Ln center can induce shifting in the observed resonances of NMR active nuclei.^56–58^ Paramagnetic shifting can be harnessed to help elucidate the solution structure of biomolecules, with the observed Ln induced shifts (LIS’s) depending on the distance and orientation of NMR active nuclei with respect to the Ln. MRI also takes advantage of paramagnetic shifting by moving features of interest away from the diamagnetic window to selectively increase image contrast, while enhancement of T_1_ relaxation rates of proximal water molecules can increase the signal per unit time.^49,56^ More recently, Ln complexes have found application in magnetic resonance thermometry, which relies on the temperature dependence of LIS’s, where variations in peak positions as a function of temperature provides a noninvasive method to monitor changes in body temperature.^59–64^

Though the use of Ln complexes with organic ligands for these applications is common, there are fewer instances of LnPOM complexes in these fields.^65–73^ Instead, the majority of NMR spectroscopy reported on LnPOMs focuses on understanding the solution speciation of these compounds and how this compares to single crystal X-ray diffraction (SCXRD) structures.^23,74–84^ Notable exceptions come from the groups of Kazansky and Pope.^85–87^ Kazansky and co-workers systematically interrogated the ^183^W and ^17^O NMR spectra of [M­(W_5_O_18_)_2_]^9–^ (M = Y^III^, La^III^, Ce^III^, Pr^III^, Nd^III^, Sm^III^, Gd^III^, Tb^III^, Dy^III^, and Ho^III^) complexes, examining the position dependence of LIS’s, while Pope and co-workers perform similar studies examining the ^31^P NMR spectra of [MP_5_W_30_O_110_]^X–^ (M = Na^I^, Ca^II^, Bi^III^. Y^III^, Nd^III^, Sm^III^, Eu^III^, Gd^III^, Tb^III^, Dy^III^, Er^III^, Tm^III^, Yb^III^, Lu^III^, Ce^IV^, and U^IV^; X = 11–14) complexes. Notably, temperature dependence of chemical shifts associated with the ^183^W and ^17^O NMR spectra is only briefly mentioned.^86^

Recently, we built upon the work of Villanneau and co-workers to isolate four cerium-containing polyoxoalkoxide sandwich-type complexes with the general formula (TBA)_*x*_[Ce­{M_4_O_13_(OMe)_4_MoNO}_2_] (M = Mo or W; X = 2, 3; TBA = tetrabutylammonium).^88,89^ These complexes are readily enriched with ^17^O and can be studied in organic media which prevents complication with loss of the ^17^O label via exchange with nonenriched solvent molecules.^90,91^ The ^17^O NMR spectrum obtained for (TBA)_3_[Ce­{Mo_5_O_13_(OMe)_4_NO}_2_] (**Ce­(Mo**_**5**_ **)**_**2**_) shows that the chemical shift of the oxygen nuclei close to cerium center is strongly influenced by the presence of the unpaired electron of cerium, with the peak occurring ca. 150 ppm downfield of where the resonance is expected. Furthermore, variable temperature (VT) ^17^O NMR shows that the position of this peak shifts over 40 ppm over a 100 °C range (−20 °C → 80 °C).^88^ These effects are diminished when examining the resonances of the ^17^O nuclei further away from cerium, in line with the expected distance dependence of LIS.^57,88^ Given that cerium typically produces smaller LIS’s compared to later Ln’s, we hypothesized that ^17^O enriched, late-Ln centered (e.g., Tb–Tm) complexes would display more significant LIS’s for nuclei close to the Ln center, and temperature dependent behavior that is competitive with known NMR thermometers.^92^

Previous works from Villanneau, Wei, and Transue (and co-workers) have led to the characterization of a large portion of the (TBA)_3_[Ln­{Mo_5_O_13_(OMe)_4_NO}_2_] series (**Ln­(Mo**_**5**_ **)**_**2**_, Figure 1).^93,94^ The complexes are mainly characterized by SCXRD and magnetic methods, with solution spectroscopy, in particular NMR spectroscopy, absent. Herein, we present a single, robust, high yielding method for the synthesis of the complete Ln series (apart from *Pm*) and access the yttrium centered complex (TBA)_3_[Y­{Mo_5_O_13_(OMe)_4_NO}_2_] (**Y­(Mo**_**5**_ **)**_**2**_). The new complexes were characterized by SCXRD, while all complexes were characterized by ^1^H/^17^O NMR spectroscopy, cyclic voltammetry, infrared spectroscopy, electronic absorption (UV–vis) spectroscopy, and elemental analysis.^17,18^ With a complete series of Ln complexes in hand, we performed a thorough analysis of the structural metrics. Importantly, we found that the **Ln­(Mo**_**5**_ **)**_**2**_ series is distinct from related series (e.g., [Ln­(W_5_O_18_)_2_]^9–^ or [Ln­(PW_11_O_39_)_2_]^11–^) in that the presence of large noncoordinating TBA cations leads to all complexes being isostructural. This means the only changes in the structures are driven by the reduction in the ionic radius of the Ln, and the concomitant reduction in Ln–O bond distances.

The absence of strong cation–anion interactions and the resulting structural consistency across the series reduces the likelihood of significant variations in coordination environment, enabling more controlled comparison of Ln-dependent NMR properties. Both ^1^H and ^17^O NMR spectroscopy reveals that the spectra are Ln dependent, with late Ln’s driving huge deviations in the chemical shifts of resonances compared to the positions of the corresponding resonances in the spectra of diamagnetic analogues. The observed LIS’s are also highly position dependent, in line with previous reports.^86^ The largest LIS is observed for the ^17^O nuclei in the Tb–O–Mo bridges of (TBA)_3_[Tb­{Mo_5_O_13_(OMe)_4_NO}_2_] (**Tb­(Mo**_**5**_ **)**_**2**_), which appears ca. 2100 ppm away from the same peak in the spectrum of **Y­(Mo**_**5**_ **)**_**2**_. After seeing these large LIS’s at room temperature, we investigated the temperature dependence of the ^1^H and ^17^O NMR spectra of the Er and Tb derivatives. The nuclei further away from the Ln center display smaller temperature-dependent shifts, consistent with a dominant pseudocontact contribution that decreases with distance. In contrast, nuclei in closer proximity to the Ln center exhibit larger shifts, which may reflect enhanced pseudocontact contributions and, for directly bound nuclei, a possible additional Fermi contact contribution. The Tb–O–Mo nuclei are the most sensitive to temperature, with the resonance associated with these nuclei shifting by ca. 7.2 ppm/K.

## Experimental Section

### General Considerations

All air- and moisture-sensitive manipulations were performed under an inert atmosphere using established protocols. Anhydrous, deoxygenated solvents were obtained from a Seca or a Pure Process Technology, LLC solvent purification system and stored over activated 4 Å molecular sieves. Deuterated solvents were purchased from Cambridge Isotope Laboratories, dried over molecular sieves, and deoxygenated by three consecutive freeze–pump–thaw cycles before use. ACS-grade Ln salts, LnX_3_ (X = Br, Cl, OTf), were purchased from Strem Chemicals, Alfa Aesar (now Thermo Fisher Scientific), and MilliporeSigma and used as received. Tetrabutylammonium hexafluorophosphate (TBAPF_6_) was purchased from Oakwood Chemical and recrystallized three times from hot ethanol before used. ^17^O-enriched water (40 atom % ^17^O) was purchased from CortecNet. All other reagents were purchased from commercial sources (Fisher Scientific, VWR, and MilliporeSigma) and used without further purification. The precursor compounds (TBA)_4_[Mo_8_O_26_][Bibr ref95] and (TBA)_2_[Mo_5_O_13_(OMe)_4_NO]­[Na­(MeOH)] (**NaMo**_**5**_)^96^ were synthesized according to literature procedures. (TBA)_4_[Sr­{Mo_5_O_13_(OMe)_4_NO}_2_] (**Sr­(Mo**_**5**_ **)**_**2**_) was synthesized based on reported procedure;^89^ here we report the SCXRD structure (single crystals were obtained by vapor diffusion of Et_2_O into the concentrated reaction mixture).

### General Procedure for the Synthesis of (TBA)_3_[M^III^{Mo_5_O_13_(OMe)_4_NO}_2_] (M = Y, La, Ce, Pr, Nd, Sm, Eu, Gd, Tb, Dy, Ho, Er, Tm, Yb, and Lu)

In a 15 mL pressure vessel, a solution of the requisite Ln salt MX_3_, (X = Br, Cl, or OTf; 0.20 mmol, 1.1 equiv) in methanol (4 mL) was added to a purple methanolic solution (4 mL) of the precursor complex **NaMo**_**5**_ (0.5 g, 0.36 mmol, 2 equiv). The sealed vessel was heated to 50 °C with stirring for 2 h, during which the solution turned red (M = Ce) or blue-purple (all other metals). The hot reaction mixture was subjected to gravity filtration to remove insoluble byproducts. The resulting filtrate was cooled to ambient temperature and subsequently stored at −30 °C overnight, which afforded the product as block-shaped crystals. These crystals were isolated from the mother liquor by filtration, washed sequentially with cold methanol (2 mL) and diethyl ether (2 × 10 mL), and dried *in vacuo*. Further crystalline material was also obtained via slow vapor diffusion of Et_2_O into a saturated acetonitrile solution of the title compound. ^17^O NMR enriched analogues are obtained following the same procedure with **NaMo**_**5**_ which has been ^17^O enriched using our previously reported method.^97^

### Y­(Mo_5_)_2_

Block shaped crystals (0.331 g, 72% yield). ^1^H NMR (500 MHz, CD_3_CN, δ) (ppm) 4.57 (s, 24H, OMe^−^), 3.17 (t, 24H), 1.68 (m, 24H), 1.42 (m, 24H), 1.00 (t, 36H). ^17^O NMR (400 MHz, CD_3_CN, δ) (ppm) 18.5 (μ_5_–O), 490.7 (Mo–O–Mo), 723.6 (Y–O–Mo), 885.5 (Mo = O). λ_max_ (MeCN) = 562 nm (ε = 118 mol^–1^ dm^3^ cm^–1^). Anal. Calcd for C_56_H_132_N_5_Mo_10_O_36_Y (mol. wt 2499.96 g mol^–1^): C, 26.90%; H, 5.32%; N, 2.80%. Found: C, 26.806%; H, 5.147%; N, 2.772%.

### La­(Mo_5_)_2_

Block shaped crystals (0.331 g, 72% yield). ^1^H NMR (500 MHz, CD_3_CN, δ) (ppm) 4.57 (s, 24H, OMe^−^), 3.18 (t, 24H), 1.69 (m, 24H), 1.43 (m, 24H), 1.01 (t, 36H). ^17^O NMR (400 MHz, CD_3_CN, δ) (ppm) 16.4 (μ_5_–O), 486.7 (Mo–O–Mo), 745.6 (La–O–Mo), 886.9 (Mo = O). λ_max_ (MeCN) = 556.5 nm (ε = 149 mol^–1^ dm^3^ cm^–1^). Anal. Calcd for C_56_H_132_N_5_Mo_10_O_36_La.MeOH (mol. wt with solvent 2582.12 g mol^–1^; without solvent 2550.08 g mol^–1^): C, 26.36%; H, 5.21%; N, 2.74%. Found: C, 26.285%; H, 5.666%; N, 2.693%.

### Ce­(Mo_5_)_2_

Reported in our previous study.^88^

### Pr­(Mo_5_)_2_

Block shaped crystals (0.299 g, 65% yield). ^1^H NMR (500 MHz, CD_3_CN, δ) (ppm) 5.48 (s, 24H, OMe^−^), 2.64 (t, 24H), 1.18 (m, 24H), 0.99 (m, 24H), 0.74 (t, 36H). ^17^O NMR (400 MHz, CD_3_CN, δ) (ppm) 18.3 (μ_5_–O), 440.0 (Mo–O–Mo), 787.4 (Pr–O–Mo), 842.9 (Mo = O). λ_max_ (MeCN) = 558 nm (ε = 107 mol^–1^ dm^3^ cm^–1^). Anal. Calcd for C_56_H_132_N_5_Mo_10_O_36_Pr (mol. wt 2551.96 g mol^–1^): C, 26.36%; H, 5.21%; N, 2.74%. Found: C, 26.399%; H, 5.065%; N, 2.682%.

### Nd­(Mo_5_)_2_

Block shaped crystals. (0.317 g, 69% yield). ^1^H NMR (500 MHz, CD_3_CN, δ) (ppm) 3.60 (s, 24H, OMe^–^), 3.54 (t, 24H), 2.02 (m, 24H), 1.72 (m, 24H), 1.21 (t, 36H). ^17^O NMR (54.3 MHz, CD_3_CN, δ) (ppm) −59.4 (μ_5_–O), 478.9 (Mo–O–Mo), 870.7 (Nd–O–Mo), 1160.4 (Mo = O). λ_max_ (MeCN) = 556 nm (ε = 195 mol^–1^ dm^3^ cm^–1^). Anal. Calcd for C_56_H_132_N_5_Mo_10_O_36_Nd (mol. wt 2555.29 g mol^–1^): C, 26.32%; H, 5.21%; N, 2.74%. Found: C, 26.251%; H, 4.841%; N, 2.828%.

### Sm­(Mo_5_)_2_

Block shaped crystals. (0.332 g, 72% yield). ^1^H NMR (500 MHz, CD_3_CN, δ) (ppm) 4.08 (s, 24H, OMe^–^), 3.42 (t, 24H), 1.91 (m, 24H, interferes with the solvent peak), 1.62 (m, 24H), 1.14 (t, 36H). ^17^O NMR (54.3 MHz, CD_3_CN, δ) (ppm) 9.9 (μ_5_–O), 487.5 (Mo–O–Mo), 702.3 (Sm–O–Mo), 883.9 (Mo = O). λ_max_ (MeCN) = 560 nm (ε = 188 mol^–1^ dm^3^ cm^–1^). Anal. Calcd for C_56_H_132_N_5_Mo_10_O_36_Sm (mol. wt 2561.41 g mol^–1^): C, 26.26%; H, 5.19%; N, 2.73%. Found: C, 26.233%; H, 5.050%; N, 2.640%.

### Eu­(Mo_5_)_2_

Block shaped crystals. (0.314 g, 68% yield). ^1^H NMR (500 MHz, CD_3_CN, δ) (ppm) 5.59 (s, 24H, OMe^–^), 2.59 (t, 24H), 1.12 (m, 24H), 0.92 (m, 24H), 0.67 (t, 36H). ^17^O NMR (54.3 MHz, CD_3_CN, δ) (ppm) −14.8 (Eu–O–Mo), 112.4 (μ_5_–O), 495.4 (Mo–O–Mo), 889.9 (Mo = O). λ_max_ (MeCN) = 562 nm (ε = 143 mol^–1^ dm^3^ cm^–1^). Anal. Calcd for C_56_H_132_N_5_Mo_10_O_36_Eu (mol. wt 2563.02 g mol^–1^): C, 26.24%; H, 5.19%; N, 2.73%. Found: C, 25.996%; H, 5.005%; N, 2.658%.

### Gd­(Mo_5_)_2_

Block shaped crystals. (0.337 g, 73% yield). Paramagnetic broadening of the ^1^H NMR signals prevented characterization of this complex by this analytical mode, therefore subsequent ^17^O NMR analysis was not performed. λ_max_ (MeCN) = 564 nm (ε = 145 mol^–1^ dm^3^ cm^–1^). Anal. Calcd for C_56_H_132_N_5_Mo_10_O_36_Gd (mol. wt 2568.30 g mol^–1^): C, 26.19%; H, 5.18%; N, 2.73%. Found: C, 26.102%; H, 4.982%; N, 2.703%.

### Tb­(Mo_5_)_2_

Block shaped crystals. (0.324 g, 70% yield). ^1^H NMR (500 MHz, CD_3_CN, δ) (ppm) 16.98 (t, 24H), 15.05 (m, 24H), 13.59 (m, 24H), 9.11 (t, 36H), −29.80 (s, 24H, OMe^–^). ^17^O NMR (54.3 MHz, CD_3_CN, δ) (ppm) −1371.5 (Tb–O–Mo), −367.4 (μ_5_–O), 384.2 (Mo–O–Mo), 844.8 (Mo = O). λ_max_ (MeCN) = 564 nm (ε = 163 mol^–1^ dm^3^ cm^–1^). Anal. Calcd for C_56_H_132_N_5_Mo_10_O_36_Tb (mol. wt 2569.98 g mol^–1^): C, 26.17%; H, 5.18%; N, 2.73%. Found: C, 25.896%; H, 5.017%; N, 2.578%.

### Dy­(Mo_5_)_2_

Block shaped crystals. (0.334 g, 72% yield). ^1^H NMR (500 MHz, CD_3_CN): δ (ppm) 7.51 (t, 24H), 5.89 (m, 24H), 5.38 (m, 24H), 3.80 (t, 36H), −7.02 (s, 24H, OMe^–^). ^17^O NMR (54.3 MHz, CD_3_CN) δ (ppm) −1027.7 (Dy–O–Mo), −94.1 (μ_5_–O), 450.3 (Mo–O–Mo), 837.1 (Mo = O). λ_max_ (MeCN) = 562 nm (ε = 140 mol^–1^ dm^3^ cm^–1^). Anal. Calcd for C_56_H_132_N_5_Mo_10_O_36_Dy (mol. wt 2573.55 g mol^–1^): C, 26.17%; H, 5.14%; N, 2.72%. Found: C, 26.700%; H, 5.042%; N, 2.690%.

### Ho­(Mo_5_)_2_

Block shaped crystals. (0.315 g, 68% yield). ^1^H NMR (500 MHz, CD_3_CN): δ (ppm) 8.29 (t, 24H), 6.60 (m, 24H), 5.84 (m, 24H), 3.96 (t, 36H), −9.20 (s, 24H, OMe^–^). ^17^O NMR (54.3 MHz, CD_3_CN) δ (ppm) −669.4 (Ho–O–Mo), −121.7 (μ_5_–O), 449.4 (Ho–O–Mo), 841.1 (Mo = O). λ_max_ (MeCN) = 566 nm (ε = 124 mol^–1^ dm^3^ cm^–1^). Anal. Calcd for C_56_H_132_N_5_Mo_10_O_36_Ho (mol. wt 2575.98 g mol^–1^): C, 26.11%; H, 5.17%; N, 2.72%. Found: C, 26.366%; H, 4.971%; N, 2.620%.

### Er­(Mo_5_)_2_

Block shaped crystals. (0.320 g, 69% yield). ^1^H NMR (500 MHz, CD_3_CN, δ) (ppm) 20.01 (s, 24H, OMe^−^), −2.44 (t, 36H), −2.90 (m, 24H), −3.85 (m, 24H), −4.16 (t, 24H). ^17^O NMR (54.3 MHz, CD_3_CN, δ) (ppm) −392.4 (Er–O–Mo), 267.8 (μ_5_–O), 542.0 (Mo–O–Mo), 886.8 (Mo = O). λ_max_ (MeCN) = 566 nm (ε = 128 mol^–1^ dm^3^ cm^–1^). Anal. Calcd for C_56_H_132_N_5_Mo_10_O_36_Er (mol. wt 2578.31 g mol^–1^): C, 26.09%; H, 5.16%; N, 2.72%. Found: C, 26.389%; H, 4.953%; N, 2.583%.

### Tm­(Mo_5_)_2_

Block shaped crystals. (0.328 g, 71% yield). ^1^H NMR (500 MHz, CD_3_CN, δ) (ppm) 44.68 (s, 24H, OMe^–^), −21.94 (t, 36H), −38.69 (m, 24H), −43.13 (m, 24H), −47.60 (t, 24H). ^17^O NMR (54.3 MHz, CD_3_CN, δ) (ppm) 36.6 (Tm–O–Mo), 608.9 (μ_5_–O), 642.1 (Mo–O–Mo), 890.0 (Mo = O). λ_max_ (MeCN) = 565.0 nm (ε = 136 mol^–1^ dm^3^ cm^–1^). Anal. Calcd for C_56_H_132_N_5_Mo_10_O_36_Tm (mol. wt 2579.99 g mol^–1^): C, 26.07%; H, 5.16%; N, 2.71%. Found: C, 26.096%; H, 5.069%; N, 2.658%.

### Yb­(Mo_5_)_2_

Block shaped crystals. (0.302 g, 65% yield). ^1^H NMR (500 MHz, CD_3_CN, δ) (ppm) 11.92 (s, 24H, OMe^–^), −0.21 (t, 24H), −0.87 (t, 36H), −1.45 (m, 24H), −1.54 (m, 24H). ^17^O NMR (54.3 MHz, CD_3_CN, δ) (ppm) 129.7 (μ_5_–O), 487.7 (Mo–O–Mo), 518.3 (Yb–O–Mo), 883.6 (Mo = O). λ_max_ (MeCN) = 566.5 nm (ε = 100 mol^–1^ dm^3^ cm^–1^). Anal. Calcd for C_56_H_132_N_5_Mo_10_O_36_Yb (mol. wt 2584.09 g mol^–1^): C, 26.03%; H, 5.15%; N, 2.71%. Found: C, 26.673%; H, 4.893%; N, 2.577%.

### Lu­(Mo_5_)_2_

Block shaped crystals. (0.321 g, 69% yield). ^1^H NMR (500 MHz, CD_3_CN, δ) (ppm) 4.57 (s, 24H, OMe^–^), 3.18 (t, 24H), 1.69 (t, 24H), 1.43 (m, 24H), 1.01 (m, 36H). ^17^O NMR (54.3 MHz, CD_3_CN, δ) (ppm) 16.4 (μ_5_–O), 486.7. (Mo–O–Mo), 745.6 (Lu–O–Mo), 886.9 (Mo = O). λ_max_ (MeCN) = 563.5 nm (ε = 148 mol^–1^ dm^3^ cm^–1^). Anal. Calcd for C_56_H_132_N_5_Mo_10_O_36_Lu (mol. wt 2586.02 g mol^–1^): C, 26.01%; H, 5.14%; N, 2.71%. Found: C, 25.896%; H, 5.453%; N, 2.724%.

### Physical Measurements

^1^H NMR spectra were acquired at ambient temperature on a 500 MHz Bruker AVANCE spectrometer, with ^1^H chemical shifts reported relative to tetramethylsilane and referenced to the residual deuterated solvent signal. ^17^O NMR spectra were collected on a JEOL 400 MHz spectrometer with chemical shifts referenced to an external standard of D_2_O. Variable-temperature (VT) ^1^H and ^17^O NMR spectra were collected on a JEOL 400 MHz spectrometer over the temperature range from 80 °C to −40 °C in 10 °C increments, maintaining sample integrity by operating within ±2 °C of CD_3_CN phase-transition limit. Electrochemical analyses were performed via cyclic voltammetry (CV) within an MBraun UniLab glovebox using a Bio-Logic SP-150 potentiostat/galvanostat and a three-electrode cell, which comprised a 3 mm glassy carbon working electrode, a platinum wire auxiliary electrode, and a Ag/Ag^+^ nonaqueous reference electrode (0.01 M AgNO_3_ in 0.1 M TBAPF_6_/acetonitrile). All electrochemical measurements were conducted with an analyte concentration of 1 mM and a supporting electrolyte (TBAPF_6_) concentration of 100 mM, and potentials were subsequently referenced to the ferrocenium/ferrocene (Fc^+^/^0^) redox couple, which was added as an internal standard. Electronic absorption spectra were recorded in anhydrous acetonitrile using an Agilent Cary 6000i UV–vis–NIR spectrophotometer in sealed 1 cm quartz cuvettes at room temperature. All elemental analysis data were obtained from the Elemental Analysis Facility at the University of Rochester.

### X-ray Crystallography

Crystals were placed onto a nylon loop and mounted on a Rigaku XtaLAB Synergy-S Dualflex diffractometer equipped with a HyPix-6000HE HPC area detector for data collection at 100.00(10) K. A preliminary set of cell constants and an orientation matrix were calculated from a small sampling of reflections.^98^ A short pre-experiment was run, with both CuKα and MoKα radiation, from which an optimal data collection strategy was determined. All data for the reported crystal structures were ultimately collected with CuKα radiation, as the results of the pre-experiments indicated that using MoKα radiation did not offer any significant improvement in structure quality, but greatly increased the collection time. After the intensity data were corrected for absorption, the final cell constants were calculated from the full data set from the xyz centroids of the strong reflections.^98^ The structures were solved using SHELXT and refined using SHELXL.^99,100^ Full-matrix least-squares/difference Fourier cycles were performed to assign the non-hydrogen atoms. All non-hydrogen atoms were first refined isotropically, followed by using anisotropic displacement parameters. All hydrogen atoms were placed in ideal positions and refined as riding atoms with relative isotropic displacement parameters.

## Results & Discussion

### Synthesis of (TBA)_3_[Ln^3+^{Mo_5_O_13_(OMe)_4_NO}_2_] Complexes

As mentioned in the introduction of this work, a report from Wei and co-workers has described the syntheses of Ln-centered polyoxoalkoxomolybdate clusters of the general formula, (TBA)_3_[Ln^3+^{Mo_5_O_13_(OMe)_4_NO}_2_] (**Ln­(Mo**_**5**_ **)**_**2**_; Ln = La, Ce, Nd, Sm, Eu, Gd, Tb, Dy, Ho, and Er).^94^ In this approach, (TBA)_4_[Mo_8_O_26_], dicyclohexylcarbodiimide (DCC), NH_2_OH, and the appropriate Ln­(NO_3_)_3_•6H_2_O salt were refluxed in methanol. The *N*,*N*′-dicyclohexylurea (DCU) and (TBA)_2_[Mo_6_O_19_] byproducts were removed by filtration and the target complexes were isolated by crystallization. Though this one-pot approach limits the number of prerequisite steps required to access **Ln­(Mo**_**5**_ **)**_**2**_ complexes, the yields are variable (22–63%) and this method requires the use of significant quantities of DCC, which is known to cause occupational allergic contact dermatitis.^101^ An alternative synthesis of a Ln centered polyoxoalkoxide sandwich-type complex was recently reported by our group; (TBA)_3_[Ce­{Mo_5_O_13_(OMe)_4_NO}_2_] (**Ce­(Mo**_**5**_ **)**_**2**_) was isolated using a modified version of Villanneau’s method, in which the addition of **NaMo**_**5**_ to half an equivalent of Ce­(OTf)_3_ in methanol yields the target complex.^88,89^

Interested in developing a single, high-yielding pathway allowing for formation of a complete series of **Ln­(Mo**_**5**_ **)**_**2**_ complexes, we postulated that it may be possible to extend the approach used to synthesize **Ce­(Mo**_**5**_ **)**_**2**_ to other Ln’s. Indeed, performing the synthesis with other LnX_3_ (X = Cl, Br, OTf) salts proved effective. The series of **Ln­(Mo**_**5**_ **)**_**2**_ complexes were synthesized via addition of **NaMo**_**5**_ to the appropriate LnX_3_ precursor in methanol (see experimental section for details). This protocol achieves high-yields of the target complexes (65–75%) in crystalline form. Characterization of the complexes by SCXRD and multinuclear NMR spectroscopy is discussed in detail below. The complexes were further characterized by cyclic voltammetry, with three one electron reduction events observed in the voltammograms of all the Ln complexes studied (Figures S46–59), matching the three reduction events previously observed in the reported CV of **Ce­(Mo**_**5**_ **)**_**2**_.^88^ The CV of **Ce­(Mo**_**5**_ **)**_**2**_ also shows a reversible Ce^IV/III^ redox couple at 0.38 V vs Fc^+/0^, however no reversible oxidation processes are observed in the CVs of the other **Ln­(Mo**_**5**_ **)**_**2**_ complexes studied.^88^ The reduction events are attributed to sequential reduction of the **Mo**_**5**_ ligands, in line with our previous work on (TBA)_2_[M^IV^{Mo_5_O_13_(OMe)_4_NO}_2_] (M^IV^ = Zr, Hf, Th, U, Np) systems.^97,102^ The **Ln­(Mo**_**5**_ **)**_**2**_ (M^3+^ centered) complexes have access to one less reduction event than the M^IV^ centered derivatives, which is attributed to the increase in negative charge of the assembly associated with replacing the central M^IV^ ion with a Ln^3+^ ion. Consistency was also found in the infrared spectra of the complexes (Figures S60–S74). The electronic absorption spectra of the **Ln­(Mo**_**5**_ **)**_**2**_ complexes all feature a low intensity absorption (ε = 100–200 mol^–1^ dm^3^ cm^–1^) at 555–570 nm, which has previously been observed in the spectra of complexes containing the [Mo_5_O_13_(OMe)_4_NO]^3–^ unit and is attributed to a transition between filled orbitals on the {Mo–NO}^4^ unit and empty orbitals of the polyoxomolybdate with primarily Mo 4d character.^88,89,103^ The energy of this transition tends to decrease as the ionic radius, and thus Lewis acidity, of the Ln increases.^102^

### Solid-State Structural Analysis of (TBA)_3_[Ln^3+^{Mo_5_O_13_(OMe)_4_NO}_2_] Complexes

Isostructural complexes spanning the entire Ln series are relatively rare, as subtle differences in ionic radius, coordination preferences, and crystal packing often drive polymorphism. Examples such as [Ln­(α-PW_11_O_39_)_2_]^11–^ and [Ln­(W_5_O_18_)_2_]^9–^ demonstrate that while the complete Ln series can be obtained for polyoxometalate sandwich-type complexes, interactions with charge-compensating alkali cations influence Ln coordination environments and overall solid-state structures.^24,104^ We hypothesized that the use of organosubstituted polyoxoalkoxide anions as low-charge ligands for Lns would eliminate the need for large equivalencies of alkali ions to compensate charge. Additionally, employment of noncoordinating TBA counter cations would suppress secondary interactions that perturb the coordination geometry at the Ln^3+^ center. Together these molecular design strategies would favor the formation of an isostructural series of Ln complexes, providing opportunities to investigate structural perturbations that result purely from the identity of the 4f-element.

To examine how Ln identity influences the structure of the **Ln­(Mo**_**5**_ **)**_**2**_ assemblies under these conditions, SCXRD data were analyzed for the entire series of complexes. We note that prior work has described the structural data for **Ln­(Mo**_**5**_ **)**_**2**_, where Ln = La, Ce, Pr, Nd, Sm, Eu, Gd, Tb, Dy, Ho, and Er.^89,93,94^ Notably all crystals possess similar unit cells, thus validating our hypothesis that the use of polyoxoalkoxide clusters and TBA^+^ ions results in isomorphic structures. The asymmetric unit cell of each structure consists of a complete (TBA)_3_[Ln­(Mo_5_O_13_(OMe)_4_NO)_2_] compound. The two polyoxoanion metalloligand **Mo**_**5**_ units ({Mo_5_O_13_(OMe)_4_NO)_2_}^3–^) are each bound to the central Ln through four oxido groups that constitute the lacunary face of the assembly. This results in an eight-coordinate geometry about the Ln^3+^ center that is best described as a distorted square antiprism (SAPR) with approximate D_4_d local symmetry.

To complete the series of sandwich-type **Ln­(Mo**_**5**_ **)**_**2**_ (and **Y­(Mo**_**5**_ **)**_**2**_) complexes, SCXRD was performed on single crystals of (TBA)_3_[M­(Mo_5_O_13_(OMe)_4_NO)_2_] (Ln = Tm, Yb, Lu, and Y) grown through slow diffusion of Et_2_O into saturated methanolic solutions of the corresponding complex. As expected, refinement of data shows that all four sandwich-type complexes are isostructural with the previously reported systems (Figure 2), crystallizing in the *P*2_1_/*c* space group. Interestingly, this level of structural consistency is not seen in the related (TBA)_3_[Ln­{Mo_5_O_13_(OMe)_4_NNC_6_H_4_NO_2_}_2_] (Ln = Tb, Dy, Ho, Er, Yb) series, where the Tb and Dy derivatives crystallize in the triclinic *P*1 space group, while the Ho, Er, and Yb derivatives crystallize in the same *P*2_1_/*c* space group observed in the **Ln­(Mo**_**5**_ **)**_**2**_ series.^105^

**Figure 2:**
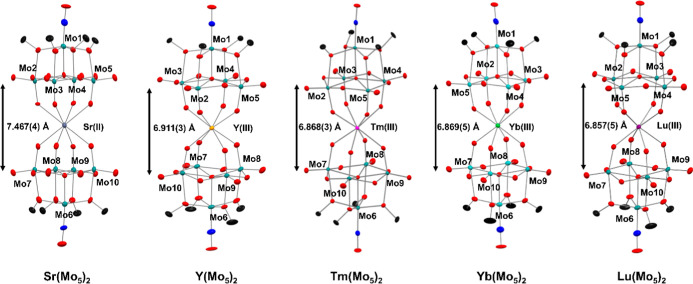
SCXRD structures of (TBA)_4_[Sr^II^{Mo_5_O_13_(OMe)_4_NO}_2_] (**Sr­(Mo**_**5**_ **)**_**2**_), (TBA)_3_[Y^III^{Mo_5_O_13_(OMe)_4_NO}_2_] (**Y­(Mo**_**5**_ **)**_**2**_), (TBA)_3_[Tm^III^{Mo_5_O_13_(OMe)_4_NO}_2_] (**Tm­(Mo**_**5**_ **)**_**2**_), (TBA)_3_[Yb^III^{Mo_5_O_13_(OMe)_4_NO}_2_] (**Yb­(Mo**_**5**_ **)**_**2**_) and (TBA)_3_[Lu^III^{Mo_5_O_13_(OMe)_4_NO}_2_] (**Lu­(Mo**_**5**_ **)**_**2**_) with probability of ellipsoids set at 50%. The tetrabutylammonium cations, solvent molecules and some disorders have been masked for clarity. Key: C, black; N, blue; O, red; Mo, teal.

With the complete series of **Ln­(Mo**_**5**_ **)**_**2**_ complexes in hand, in-depth analysis of structural data could be performed to assess periodic trends that dictate perturbations in molecular geometry across the Ln series. To rule out contributions from deviations of the metalloligand scaffold, we first evaluated the bond metrics of the individual poloxoalkoxomolybdate moieties (e.g., Mo–O, Mo–OCH_3_, Mo–O–Mo, geometry around μ_5_-O; see Table S7). Only small changes are observed, suggesting that the metalloligand is a rigid entity across the series. This observation is also supported by infrared spectroscopy (Figures S60–S74); Mo = O, NO, and O–CH_3_ stretching frequencies are invariant in the Ln-centered sandwich-type complexes.

With this information obtained, our focus shifted to the immediate coordination environment of the Ln. Across the Ln series, a monotonic decrease in average Ln-O distances is observed (Figure 3A), from 2.53 Å (La) to 2.33 Å (Lu). The observed decrease in Ln–O bond distances correlates linearly with reduction in ionic radii. This mirrors trends previously reported in Weakley-type [Ln­(W_5_O_18_)_2_]^9–^ and [Ln­{Mo_5_O_13_(OMe)_4_NNC_6_H_4_NO_2_}_2_]^3–^ complexes.^19,104–106^ The reduction in Ln–O_8_ bond distances propagates to a reduced separation between metalloligands, as is quantified through the measured distance between the two μ_5_-O moieties (d­(μ_5_-O···μ_5_-O; Figure 3B).

**Figure 3:**
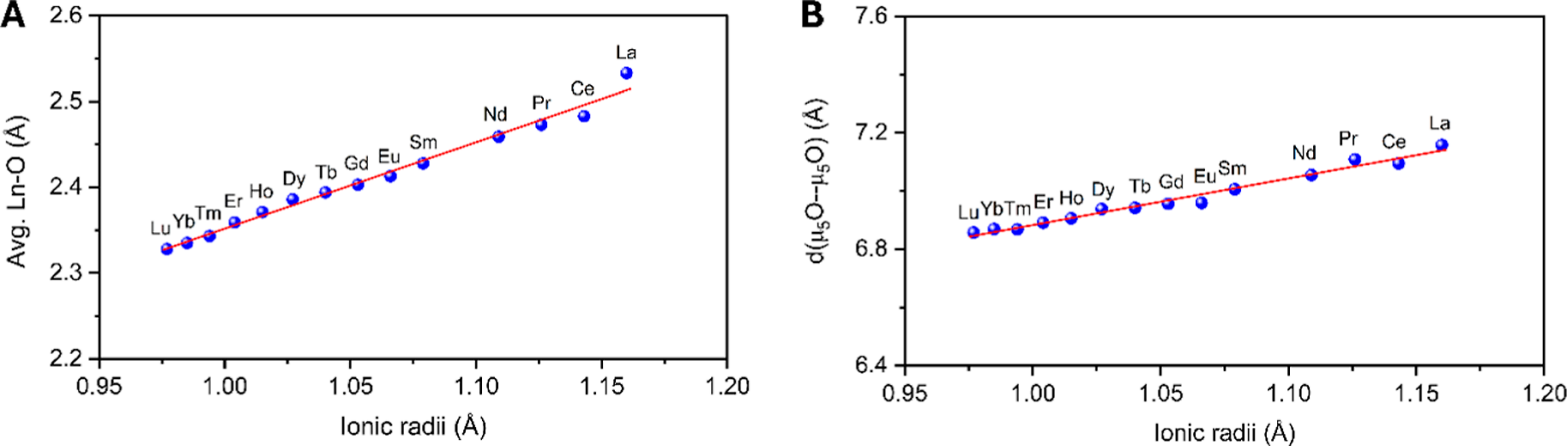
Structural parameters of **Ln­(Mo**_**5**_ **)**_**2**_ vs ionic radii. (A) Average Ln–O bond length; (B) separation between the two μ_5_-O atoms of the opposing Mo_5_ units.

Similar contraction-driven compression has been observed in other f-block POM sandwich-type systems. For example, Deblonde and co-workers reported systematic d­(P–P) reduction across the Cs_11_[Ln­(PW_11_O_39_)_2_] Keggin series.^24^ Interestingly, actinide and Ln Keggin^24^ and Wells-Dawson^107–111^ salts exhibit a key structural feature we do not observe; the O_3_PO–Ln–OPO_3_ bond angle often deviates from linearity. In contrast, the μ_5_-*O*-Ln-μ_5_-O angle in the sandwich-type polyoxoalkoxide complexes reported here remains linear across the entire series (176.8°–178.9°). We attribute this structural rigidity to the use of bulky, noncoordinating TBA^+^ counterions (approximate ionic radius of 4.9 Å).^112^ The reported Keggin and Wells-Dawson structures typically crystallize with alkali metal cations (e.g., Na^+^, K^+^, or Cs^+^, ionic radii range from 1.0 to 1.8 Å)^113^ that form direct or water-mediated bridges to the POM exterior. These coordinating alkali ions often perturb the immediate coordination environment of the Ln. By utilizing TBA^+^, we effectively eliminate these second-sphere electrostatic interactions. Similar μ_5_-*O*-Ln-μ_5_-O angles (176.6°–177.6°) are observed in isostructural (TBA)_3_[Ln­{Mo_5_O_13_(OMe)_4_NNC_6_H_4_NO_2_}_2_] (Ln = Ho, Er, and Yb) complexes, with values even closer to 180° observed in the Tb/Dy (*P*1) derivatives (Table S9).^105^

Next, we evaluated several geometric distortion parameters that report on the deviations of primary coordination spheres from ideal geometries (Figures 4 and 5), in line with previous reports analyzing the structures of LnPOM systems.^19,105,114^ Continuous shape measures (CShM) analysis was performed for the series.^88,115–117^ CShM analysis provides an overall distortion index by comparing the eight-coordinate Ln–O_8_ geometry to an ideal D_4_d square antiprism (CShM = 0 for perfect geometry, higher values indicate greater deviation from the ideal polyhedron; Figure 5A). Calculated CShM values range from 0.430 (Tm) to 0.930 (Ce) and show an overall decrease across the series (Figure 5A, Table S10), reflecting a general tendency for the Ln coordination environment to become more square antiprismatic as the Ln^3+^ ionic radius shrinks. This continuity is not observed in (TBA)_3_[Ln­{Mo_5_O_13_(OMe)_4_NNC_6_H_4_NO_2_}_2_] (Ln = Tb, Dy, Ho, Er, Yb), where the change in crystal packing between the Tb/Dy (*P*1) and Ho/Er/Yb (*P*2_1_/*c*) drives an increase in the CShM value for the later Ln’s (0.408–0.520) when compared to the earlier Ln’s (0.287–0.293) (Table S9).^105^ This illustrates the impact that intermolecular interactions within the lattice can have on the local Ln coordination environment. However, as Carter and co-workers showed for the analogous Na_9_[Ln­(W_5_O_18_)_2_]·*x*H_2_O (Ln = La­(III)–Lu­(III), except *Pm*(III)), no smooth trend (apart from Ln–O bond distances) runs through the entire Ln series. Instead, distortion parameters only track the ionic radius in a meaningful way when Ln^3+^ ions are grouped by their structural subsets (e.g., early-vs late-Ln’s). In our series, the same picture emerges, the early, lighter Ln’s (La–Eu) show consistently higher CShM values (0.633–0.930). As the ionic radius shrinks across the heavy Ln’s (Gd–Lu), the two **Mo**_**5**_ units progressively twist away from a cubic arrangement toward an ideal square antiprismatic geometry, reflected in the lower, tightly clustered CShM values (0.430–0.611). This local distortion maximum at the half-filled f^7^ configuration mirrors known electronic-structure effects in other Ln­(POM)_2_ series, where subtle changes in d- and f-orbital mixing or crystal-field splitting perturb coordination geometry, even within a single space group.^24^ Importantly, the Eu/Gd deviation from the trend is not accompanied by changes in Ln–O or d­(μ_5_-O···μ_5_-O), nor does it correspond to a structural phase transition unlike the parallel-to-perpendicular packing break seen in Cs_11_[Ln­(PW_11_O_39_)_2_].^24^ These values are comparable to or smaller than those reported for the Na_9_[Ln­(W_5_O_18_)_2_] Lindqvist series (CShM ∼0.5–1.2), where multiple polymorphs with different Na^+^/H_2_O packing arrangements led to larger and more variable distortions.^104^

**Figure 4:**
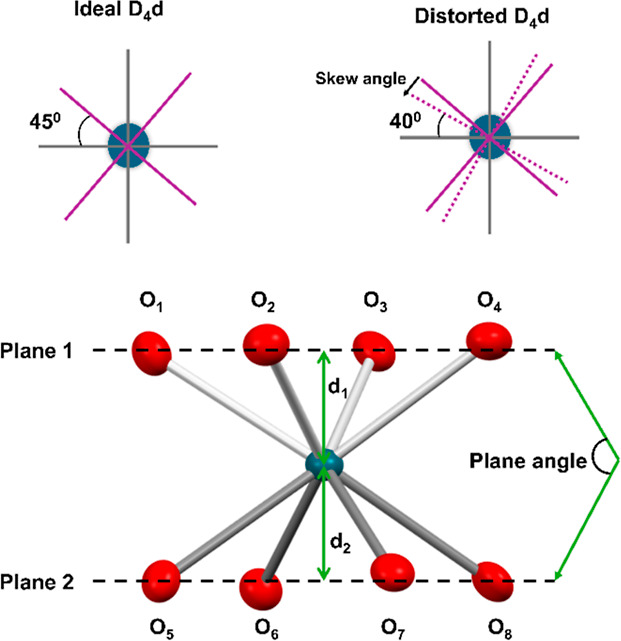
Structural parameters in **Ln­(Mo**_**5**_ **)**_**2**_ complexes. (Top) Top-down projection highlighting Ln–O_8_ cage with Ln^3+^ cation as a circle with its eight Ln-O bonds and its distortion from the ideal D_4_d symmetry. (Bottom) Side view of Ln–O_8_ cage with Ln^3+^ cation in cyan and oxygens in red color. Top and bottom O_4_ planes in black.

**Figure 5:**
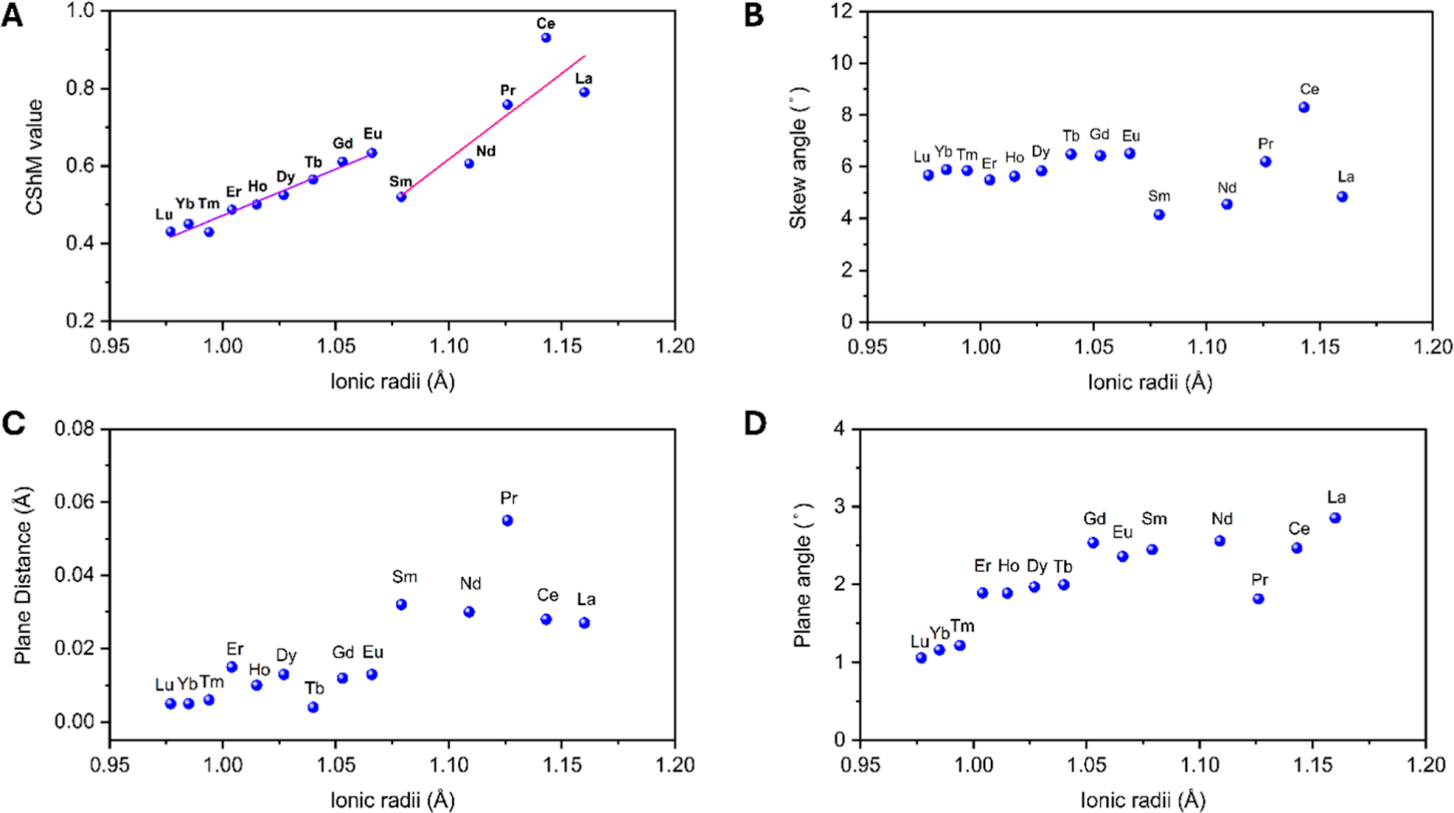
Structural distortion parameters of **Ln­(Mo**_**5**_ **)**_**2**_ vs ionic radii. (A) Continuous Shape Measurement (B) Skew angle (C) Plane distance (D) Plane angle.

We also examined the skew angle between the two **Mo**_**5**_ oxygen planes (Table S8), which tracks the rotational offset between the POM caps around the O_4_–Ln–O_4_ axis. A value of zero gives the ideal SAPR geometry, while higher values mean the O_4_ squares are twisting out of alignment toward a cubic geometry. Our calculated skew angles (Figure 5B) range from 8.30° (Ce) to 4.14° (Sm). The trends in skew angles are in line with the observed trends in CShM values, with an apparent break between the early Ln’s (La–Sm) and the late Ln’s (Eu–Lu). The later Ln’s show consistent, slightly decreasing, skew angles as the ionic radius of the Ln decreases (Eu = 6.51° → Lu = 5.67°). This is consistent with the local Ln-O_8_ geometry tending toward a more ideal SAPR as ionic radius decreases. Conversely, the skew angles of the early Ln’s are more sporadic, though there is a decrease in skew angle from Ce → Sm. The skew angles observed in the **Ln­(Mo**_**5**_ **)**_**2**_ series are similar to the isostructural (TBA)_3_[Ln­{Mo_5_O_13_(OMe)_4_NNC_6_H_4_NO_2_}_2_] (Ln = Ho, Er, Yb) complexes (skew angles = 5.01–5.86°), though the Tb and Dy derivatives (*P*1) feature significantly lower skew angles (0.91° and 0.84° respectively).^105^ This again illustrates the importance of crystal packing in dictating the local Ln coordination environment. Other Ln­(POM)_2_ sandwiches like [Ln­(W_5_O_18_)_2_]^9–^ generally also display a decreasing skew angle as the Ln ionic radius decreases, with late Ln’s approaching a more perfect D_4_d geometry.^104^

We also examined additional parameters, plane distance and plane angle, following Carter and co-workers’ analysis of the [Ln­(W_5_O_18_)_2_]^9–^ series, where they found systematic correlations between distortion metrics and low-energy vibrational modes relevant to spin–phonon coupling. The plane distance (PD = |d_1_ – d_2_|) measures how far off-center the Ln sits between the two O_4_ square faces (Figures 4 and 5C). We fit least–squares planes through each Ln–O_4_ set and calculated perpendicular Ln-to-plane distances (PD = 0 means perfectly centered). Our PD values are quite small (0.004–0.055 Å) with no clear trend, suggesting minimal axial displacement is observed across the entire Ln series. This is consistent with the values obtained for [Ln­{Mo_5_O_13_(OMe)_4_NNC_6_H_4_NO_2_}_2_]^3–^ complexes (0.009–0.015 Å, Table S9).^105^ However, the plane angle (the dihedral between the two Ln–O_4_ planes) ranges from 1.16° (Yb) to 2.86° (La), decreasing slightly as the cavity contracts (Figure 5D). This observation indicates that the two square faces defining the coordination of the two **Mo**_**5**_ metalloligands become more parallel as the ionic radii decreases. We note that these values are in line with the range of plane angles reported for [Ln­(W_5_O_18_)_2_]^9–^ (1°–5°) and [Ln­{Mo_5_O_13_(OMe)_4_NNC_6_H_4_NO_2_}_2_]^3–^ (1°–4°) complexes.^104,105^ However, the Lindqvist systems stand in direct contrast to the Cs_11_[Ln­(PW_11_O_39_)_2_] Keggin series, where bending distortions (nonlinear P–Ln–P) dominate the structural response to Ln contraction when coordinating Cs^+^ ions constrain the framework.

What distinguishes **Ln­(Mo**_**5**_ **)**_**2**_ series from other Ln­(POM)_2_ systems is the geometric consistency of all 14 complexes, where Ln contraction drives compression of the Ln–O_8_ cavity rather than angular distortions. Indeed, this observation is confirmed through the shortening of μ_5_-O···μ_5_-O and Ln–O distances, separation shortens, the plane angles adjust, and the trans μ_5_-O–Ln−μ_5_-O angles straighten. Despite these changes, no significant bending distortions, off-centering, or rotational misalignments of the two **Mo**_**5**_ units are observed. Even the Eu/Gd f^7^ anomaly visible as a deviation in the CShM trend does not disrupt the isostructural integrity of the series.

Broadening the comparison beyond Ln^3+^ to aliovalent metals (e.g., M^2+^, M^4+^) reveals that what primarily controls the degree of compression and staggering of the **Mo**_**5**_ ligands in **M­(Mo**_**5**_ **)**_**2**_ systems is ionic charge density. Ba^2+^ and Sr^2+^ centered derivatives (the structure of **Sr­(Mo**_**5**_ **)**_**2**_ is reported in this work and given in Figure 2), which feature cations with both a large radius and a low charge (ionic radii of 142 and 126 pm, respectively), give the longest separations (μ_5_-O···μ_5_-O = 7.72 Å and 7.46 Å respectively) and nearly perfect cubic Ln–O_8_ geometries (Cubic CShM = 0.39 and 0.15 respectively).^89^ Therefore, in these examples the interactions between the two **Mo**_**5**_ units of the sandwich-type complex are small and there is no driving force for staggering. When moving to the Ln^3+^ centered complexes presented in this work, a compression in the μ_5_-O···μ_5_-O distance (7.16–6.87 Å) is observed, driven by the decrease in ionic radius and increase in electrostatic attraction between the central cation and **Mo**_**5**_ ligands. This presumably leads to destabilization of the eclipsed (cubic) geometry and instead a twisting toward a staggered SAPR geometry is observed (SAPR CShM = 0.43–0.93). The tetravalent metals, both f-block (Th^4+^, U^4+^, Ce^4+^, Np^4+^, 6.94–6.81 Å) and d-block (Zr^4+^, Hf^4+^, 6.65 Å, CShM ≈0.11–0.12) maximize this effect, compressing the distance between the two **Mo**_**5**_ ligands even further, reaching near-perfect staggered (SAPR) geometry.^88,97,102^

### ^1^H and ^17^O NMR Characterization of (TBA)_3_[Ln^3+^{Mo_5_O_13_(OMe)_4_NO}_2_] Complexes

The minimal variation in structural metrics across the **Ln­(Mo**_**5**_ **)**_**2**_ series, the absence of strong cation–anion interactions, and the use of nonaqueous polar aprotic solvents (thereby avoiding protic solvents capable of strong hydrogen bonding to the POM framework) minimize the likelihood of significant changes in coordination environment in solution. This provides a controlled platform for examining the influence of Ln identity on the NMR properties of the complexes. While many of the **Ln­(Mo**_**5**_ **)**_**2**_ complexes discussed in our structural analysis have been previously reported, characterization of these complexes by NMR spectroscopy remains absent from the literature.^89,93,94^ Consequently, very little is known about how both the Ln electronic configuration and minor structural differences influence the NMR properties of the complexes. We therefore acquired both ^1^H and ^17^O NMR spectra on the full series of complexes synthesized in this work. ^17^O NMR were acquired using ^17^O enriched analogues of **Ln­(Mo**_**5**_ **)**_**2**_.^88,97^

The diamagnetic complexes **Y­(Mo**_**5**_ **)**_**2**_, **La­(Mo**_**5**_ **)**_**2**_, and **Lu­(Mo**_**5**_ **)**_**2**_ possess very similar ^1^H and ^17^O NMR spectra; the spectra of **Y­(Mo**_**5**_ **)**_**2**_ is shown in Figure 6. (Figures S1, S15 and S13, S26 for **La­(Mo**_**5**_ **)**_**2**_ and **Lu­(Mo**_**5**_ **)**_**2**_ respectively). The ^1^H NMR spectra of all three complexes possess a resonance at 4.57 ppm, assigned to the -OMe groups of the polyoxoalkoxide ligand, indicating that variation of the diamagnetic M^3+^ ion at the center of the sandwich type complex has no influence on the chemical environment of these protons. Four additional signals are observed between 1 and 4 ppm, attributed to the protons of the TBA cations, which provide charge balance to the anionic complex. The ^17^O NMR spectra of the diamagnetic complexes feature four peaks in a 4:4:4:1 ratio, assigned to the various oxo groups of the sandwich-type complexes (Figures 6, S15, and S26). The oxygen nuclei of Ln-***O***-Mo/Y–***O***–Mo bridges are the most sensitive to the nature of the M^3+^ ion present. The chemical shift of this resonance moves from 718.2 ppm in the spectrum of **Lu­(Mo**_**5**_ **)**_**2**_ to 745.6 ppm in the spectrum of **La­(Mo**_**5**_ **)**_**2**_, with the peak in the spectrum of **Y­(Mo**_**5**_ **)**_**2**_ (Figure 6, red peak) lying in between. Interestingly, the chemical shift of this peak varies linearly with the ionic radius of the M^3+^ ion present in the complex (Figure S28). This can be rationalized in terms of slight changes in the Mo–O bond order of the Mo–O-Ln/Y bridges. Increasing the size of the M^3+^ ion present results in a reduction in Lewis acidity and an increase in the M–O bond length. Together, these points contribute to a weaker interaction between Ln/Y and the oxygen nuclei of the Mo–***O***-Ln/Y bridges. This reduces competition for nonbonding electron density on oxygen (i.e., oxygen lone pairs) and thus the extent of O­(2p) → Mo­(4d) π-bonding slightly increases, leading to increased chemical shifts in ^17^O NMR spectra.^97,103,118,119^ The resonances assigned to the other oxygen nuclei in the complex (i.e., Mo = ***O***, Mo–***O***–Mo’s, and μ_5_-***O***’s) remain essentially constant across the series, with only minor variations of 2–5 ppm observed in the spectra of **Y­(Mo**_**5**_ **)**_**2**_, **La­(Mo**_**5**_ **)**_**2**_, and **Lu­(Mo**_**5**_ **)**_**2**_.

**Figure 6:**
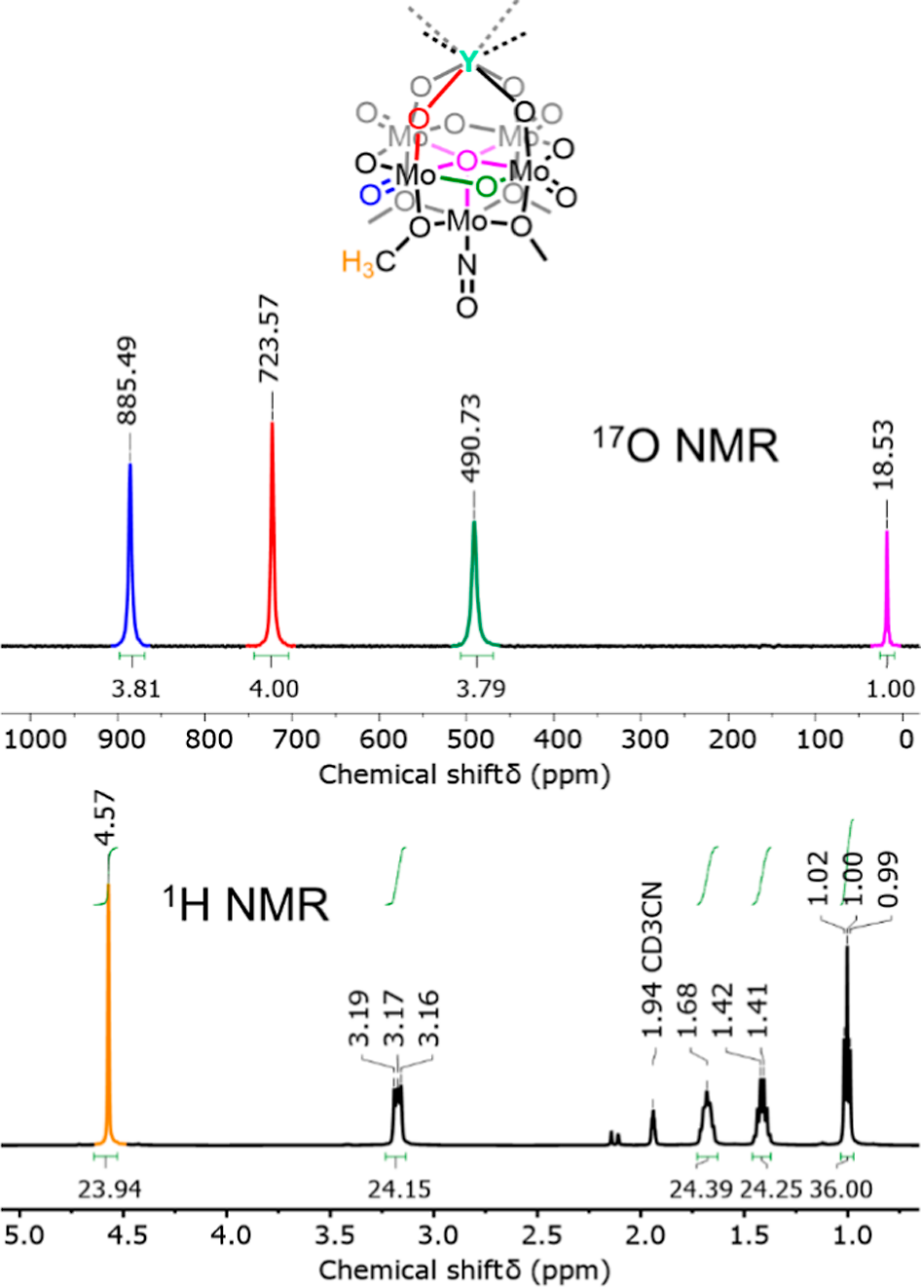
^17^O NMR (top) and ^1^H NMR (bottom) spectra of **Y­(Mo**_**5**_ **)**_**2**_. Peaks in the NMR spectra are color matched to the appropriate nuclei in the representative structure above to show assignments. Spectra were recorded at 20.8 °C in CD_3_CN.

The chemical shifts of resonances in the NMR spectra of the diamagnetic complexes discussed above can be rationalized in terms of changes in bonding and chemical environment. However, the rest of the molecules in the series of **Ln­(Mo**_**5**_ **)**_**2**_ described in this work all possess a paramagnetic central metal ion. The presence of unpaired electrons on these metal ions leads to deviations in both the chemical shift and width of the peaks present in the NMR spectra of these complexes. In these systems, the observed chemical shift (δ_*ij*_^exp^) can be described as the sum of the orbital shift (δ_*i*_^orb^), the Fermi contact shift (δ_*ij*_^FC^), and the pseudocontact shift (δ_*ij*_^PCS^), according to eq 1

1 $$\delta_{ij}^{\exp}=\delta_{i}^{orb}+\delta_{ij}^{FC}+\delta_{ij}^{PCS}$$

2 $$\delta_{ij}^{para}=\delta_{ij}^{FC}+\delta_{ij}^{PCS}=\delta_{ij}^{\exp}-\delta_{i}^{orb}$$

The orbital shift (δ_*i*_^orb^) can be approximated as the chemical shift of the corresponding peak in a diamagnetic complex and is approximately temperature independent.^88,120,121^ The deviation between this value and the observed experimental chemical shift of a nucleus *i* induced by Ln^3+^ ion *j* is often referred to as the LIS or δ_*ij*_^para^ in eq 2. The total LIS includes contributions from Fermi contact shifts (δ_*ij*_^FC^) and pseudocontact shifts (δ_*ij*_^PCS^). Fermi contact shifts originate from spin-density transfer through bonding pathways between the Ln *j* and nucleus *i*. The contact shift is given by eq 3,

3 $$\delta_{ij}^{FC}=\frac{2\pi \beta }{3kT\gamma }\frac{A}{h}\left\langle S_{z}\right\rangle$$

in which β is the Bohr magneton, *k* is the Boltzmann constant, *T* is temperature, γ is the gyromagnetic ratio of nucleus *i*, A/h is the hyperfine coupling constant in frequency units and ⟨S_*z*_⟩ is the electron-spin expectation value, which has been tabulated for each Ln^3+^ ion.^122–124^ Importantly, because Fermi contact shifting is a through bond effect, it is often considered a minor contribution toward total LIS in the paramagnetic Ln NMR literature as (1) Ln unpaired electrons lie in contracted 4f orbitals which are typically are not directly involved in bonding and, (2) NMR active nuclei are normally spatially removed from the Ln center (i.e., not directly bound).^125^ Instead, LIS is often dominated by pseudocontact shifts (δ_*ij*_^PCS^) which arise from through-space dipolar interactions between the magnetic moment of the Ln^3+^ ion *j* and the observed nucleus *i*. In the principle magnetic axis system, pseudocontact shifts are described by eq 4:^58,126,127^

4 $$\delta_{ij}^{PCS}=\frac{1}{12\pi r^{3}}\left[\chi_{ax}\left(3\cos^{2}\theta -1\right)+3\chi_{rh}\sin^{2}\theta \;\cos2\varphi \right]$$

In this equation, *r* is the distance between the Ln and the nuclear spin, χ_ax_ and χ_rh_ are the axial and rhombic components of the anisotropy component of the magnetic susceptibility tensor χ of the Ln^3+^ ion and θ/φ describe the position of the nuclear spin with respect to the principal axis.^58,126,127^ Equation 4 shows that pseudocontact shifts are dependent on the distance and orientation of the observed nucleus from the Ln^3+^ center and the nature of the Ln present. Indeed, these factors dictate the values of χ_ax_ and χ_rh_, along with crystal field parameters, according to Bleaney’s theory.^127,128^ It should be noted that this expression is derived within the point-dipole approximation, in which the paramagnetic center is treated as a localized magnetic dipole.^129^ This approximation is generally valid for nuclei sufficiently distant from the metal center but is expected to break down at short metal–nucleus distances, such as for nuclei directly coordinated to the Ln.^53^ Consequently, for these sites the equation should instead be regarded as providing a qualitative description of trends.

A summary of the observed paramagnetic shifts (δ_*ij*_^para^) of the **Ln­(Mo**_**5**_ **)**_**2**_ complexes studied in this work obtained from eq 2, where δ_*ij*_^exp^ is the experimentally observed chemical shift for a specific nucleus in the **Ln­(Mo**_**5**_ **)**_**2**_ complex being studied and δ_*i*_^orb^ is taken as the chemical shift of the same nucleus in **Y­(Mo**_**5**_ **)**_**2**_, are given in Figure 7. **Y­(Mo**_**5**_ **)**_**2**_ was selected as the diamagnetic reference complex as its “intermediate” ionic radius presents as the best “like for like” comparison to the full series of **Ln­(Mo**_**5**_ **)**_**2**_ complexes studied. Studying the obtained values shows many of the expected trends. First, the more remote nuclei, i.e. -OC ***H***_3_ and Mo = ***O***, display the smallest paramagnetic shifts across the series, suggesting Fermi contact shifts are negligible and pseudocontact shifts are relatively small at these positions (due to the 1/*r* ^3^ dependency). In general, there is a slight increase in the magnitude of the observed paramagnetic shifts when moving to the Mo–***O***–Mo bridges, consistent with the increase in pseudocontact shifting as r decreases. The two positions closest to the Ln center, i.e. μ_5_-***O*** and Ln-***O***-Mo, display much larger paramagnetic shifts. This is consistent with the strong distance dependence of pseudocontact shifting, however we cannot rule out additional Fermi contact contributions. For nuclei directly bound to the Ln, it is likely that a non-negligible Fermi contact contribution describes the paramagnetic shifts of the signals associated with these atoms. We note that similar trends have been previously observed in the ^17^O NMR spectra of [Ln­(W_5_O_18_)_2_]^9–^ systems.^85,86^

**Figure 7:**
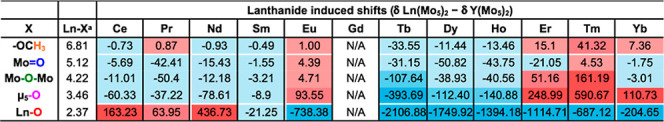
Summary of the LIS’s obtained for **Ln­(Mo**_**5**_ **)**_**2**_ complexes with respect to the diamagnetic reference complex **Y­(Mo**_**5**_ **)**_**2**_. Downfield shifts are highlighted in red, while upfield shifts are highlighted in blue. ^a^Average distances (in Å) obtained from SCXRD structure of **Y­(Mo**_**5**_ **)**_**2**_ are given as a representative example. Ln to –OCH_3_ distances approximated by the Ln → C distance.

There are clear trends in both sign and magnitude of the observed paramagnetic shifts across the Ln series. The magnitude of paramagnetic shifting is greater for the late Ln’s than the early Ln’s, with complexes containing Tb–Tm displaying LIS that are orders of magnitude higher than the corresponding shifts in in Ce–Sm complexes. These effects can be rationalized by considering the intrinsic properties of the Ln^3+^ ions. In Ln systems, the magnetic anisotropy and associated pseudocontact shifts are largely governed by the spin–orbit coupled ground state of the free ion. As a result, the sign and magnitude of the susceptibility anisotropy, and thus the pseudocontact contribution to the paramagnetic shift, is often linked to the underlying 4f electron density distribution of the ion.^130^ These factors are captured by Bleaney’s theory, in which the size and sign of χ_ax_ and χ_rh_ are dependent on Ln identity, and are found to be larger for the late Ln’s.^128,131^ A distinction between the early and late Ln’s was found in the proton relaxation behavior of the -OC ***H***_3_ groups of the **Ln­(Mo**_**5**_ **)**_**2**_ complexes studied, with the obtained longitudinal relaxation (spin–lattice, T_1_) and transverse relaxation (spin–spin, T_2_) times summarized in Table S1.

A change in the sign of the LIS for the oxygen nuclei of the Ln-O bridges of Ce, Pr, Nd, Eu, Er, and Tm is noted when compared to the other nuclei in the complex. This could be caused by the change in the sign of δ_*ij*_^PCS^, with both the sign and magnitude of δ_*ij*_^PCS^ varying with the position of the nucleus of interest with respect to the principle magnetic axis (in eq 4, 3cos^2^θ-1 is positive when θ < 54.7° and negative when θ > 54.7°).^132^ Alternatively, it may be caused by a change in the paramagnetic shift being dominated by pseudocontact shifting for more peripheral nuclei to Fermi contact shifting for Ln-O nuclei.

A key feature of the paramagnetically shifted NMR spectra of Ln complexes is that the LIS is highly sensitive to temperature.^131,133,134^ Bleaney’s theory indicates that χ_ax_ and χ_rh_ show a T^–2^ dependency according to eq 5A and 5B

5 $$A)\chi_{ax}=-\frac{\mu_{0}{\mu_{B}^{2}C}_{J}B_{0}^{2}}{10{\left(kT\right)}^{2}},;,B)\chi_{rh}=-\frac{\mu_{0}{\mu_{B}^{2}C}_{J}B_{2}^{2}}{30{\left(kT\right)}^{2}}$$

In these equations, μ_0_ is the vacuum permeability, μ_*B*_ is the Bohr magneton, *C*_*J*_ are Ln dependent Bleaney constants, *B*_0_/*B*_2_ are crystal field parameters, and *k* is the Boltzmann constant.^58^ This accounts for the temperature dependence of pseudocontact shifting, while examining eq 3 reveals a T^–1^ dependence for Fermi contact shifting. These effects combine to drive a reduction in the LIS as temperature increases, with peaks tending toward the position of the corresponding peaks in the diamagnetic reference complex (i.e., δ_*ij*_^exp^ tends toward δ_*i*_^orb^ as δ_*ij*_^para^ tends toward zero). The **Ln­(Mo**_**5**_ **)**_**2**_ complexes discussed above possess multiple NMR handles that differ in position and orientation with respect to the Ln center, and thus the relative contributions of δ_*ij*_^FC^ and δ_*ij*_^PCS^ toward δ_*ij*_^exp^ are also different. This means that we can expect the magnitude of chemical shift variation as a function of temperature will be highly dependent on the position of the NMR active nucleus within the **Ln­(Mo**_**5**_ **)**_**2**_ framework.

To investigate this phenomenon, we performed VT ^17^O NMR spectroscopy on **Tb­(Mo**_**5**_ **)**_**2**_ and **Er­(Mo**_**5**_ **)**_**2**_. These complexes were selected due to the large, position dependent, paramagnetic shifts observed in room temperature experiments. We anticipated this would lead to large differences in the temperature dependent behavior between the individual NMR active nuclei present. Furthermore, we chose to study two systems in which the direction of paramagnetic shifting differed (i.e., upfield vs downfield shifting).

The VT ^17^O NMR spectra obtained on **Tb­(Mo**_**5**_ **)**_**2**_ between −40 and 80 °C are shown in Figure 7, with the VT ^1^H NMR and corresponding data for **Er­(Mo**_**5**_ **)**_**2**_ shown in Figures S29–S31. As expected, the four ^17^O environments of **Tb­(Mo**_**5**_ **)**_**2**_ respond to temperature in different ways. The Tb–***O***–Mo bridging oxygens are the most responsive, with the resonance associated with these nuclei moving from −1897.9 ppm at −40 °C to −1034.0 ppm at 80 °C. This represents an overall shift of 863.9 ppm over a 120 °C window, equivalent to a change of ∼7 ppm/K. The temperature sensitivity observed is consistent with strong paramagnetic contributions resulting from pseudocontract shifts at this position. An additional Fermi contact contribution may be present for nuclei directly coordinated to the Ln; however, the temperature dependence alone does not allow these contributions to be disentangled. In line with the observed trends in room temperature LIS, the next most temperature sensitive position is the central μ_5_-***O*** (highlighted in magenta in Figure 8). This peak moves from −639.0 ppm to −225.1 ppm over the temperature range studied, equivalent to 3.4 ppm/K. This peak is therefore roughly half as responsive to changes in temperature when compared to the peak associated with the oxygen nuclei of the Tb–***O***–Mo bridges, clearly illustrating how even minor changes in position drive changes in δ_*ij*_^FC^ and δ_*ij*_^PCS^, and in-turn the temperature sensitivity. The peripheral atoms of the **Tb­(Mo**_**5**_ **)**_**2**_ complex, i.e. Mo–***O***–Mo, Mo = ***O***, and -OC ***H***_3_, are much less sensitive to temperature, showing shifts of 0.82, 0.23, and 0.25 ppm/K, respectively, over the investigated temperature range.

**Figure 8:**
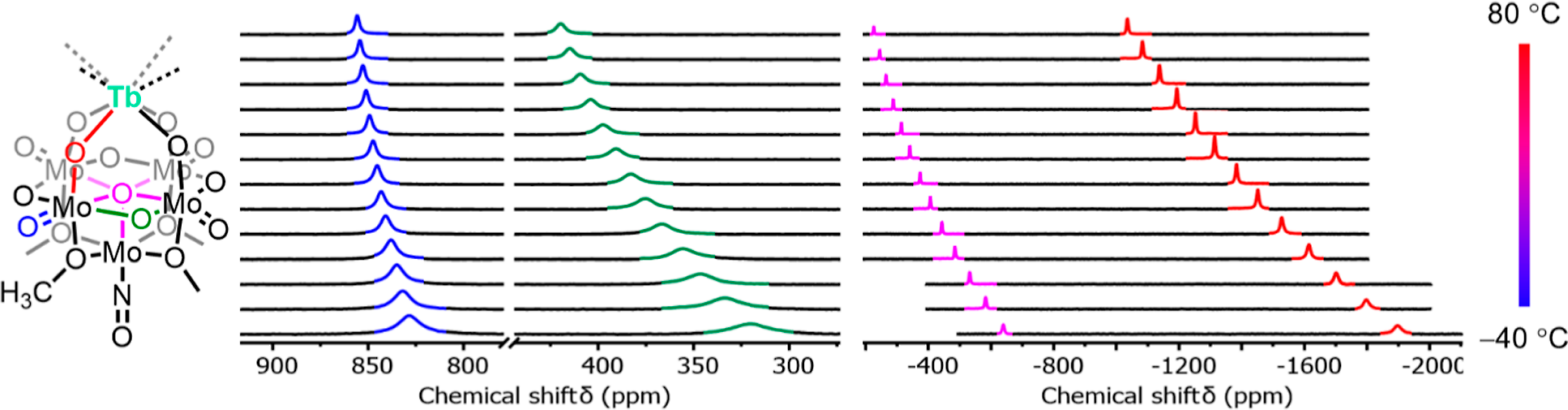
Variable Temperature ^17^O NMR spectra (54.3 MHz) of (TBA)_3_[Tb­{Mo_5_O_13_(OMe)_4_NO}_2_] (**Tb­(Mo**_**5**_ **)**_**2**_). Spectra obtained in CD_3_CN at −40 to 80 °C.

This data, along with that of **Er­(Mo**_**5**_ **)**_**2**_, is conveniently summarized in the chemical shift (ppm) vs temperature (K) plots shown in Figure 9. The same trends in the magnitude of chemical shift change per Kelvin are observed for **Er­(Mo**_**5**_ **)**_**2**_ as in **Tb­(Mo**_**5**_ **)**_**2**_, with the Ln-***O*** and μ_5_-***O*** sites being more sensitive (3.9 ppm/K and −1.75 ppm/K respectively) than the peripheral Mo–***O***–Mo Mo = ***O***, and -OC ***H***_3_ sites (−0.39 ppm/K, 0.021 ppm/K, and 0.119 ppm/K respectively). We note, however, that the temperature sensitivity of **Er­(Mo**_**5**_ **)**_**2**_ to is lower. A key difference in the data obtained for **Er­(Mo**_**5**_ **)**_**2**_ is that the peaks corresponding to the μ_5_-***O***’s and Mo–***O***–Mo’s move upfield as temperature increases, while all other peaks in the spectra of the two complexes move downfield. This reflects a change in the sign of the LIS for these resonances which means that as temperature increases, and δ_*ij*_^FC^/δ_*ij*_^PCS^ diminish, these resonances migrate upfield moving toward the corresponding peaks in **Y­(Mo**_**5**_ **)**_**2**_ (which reflects δ_*i*_^orb^). The presence of two inequivalent ^17^O nuclei which move in opposing directions as a function of temperature provides the opportunity to use the difference in chemical shift between these nuclei to provide a more sensitive temperature response than provided by any single site alone.^60^ Applying this approach to **Er­(Mo**_**5**_ **)**_**2**_ provides an enhanced sensitivity of ca. 5.7 ppm/K for δ­(μ_5_-***O***) – δ­(Ln-***O***). Though this is value is still not as high as the chemical shift change per Kelvin observed in the case of **Tb­(Mo**_**5**_ **)**_**2**_, this result demonstrates the utility of designing NMR temperature sensors which possess multiple inequivalent NMR active nuclei which display opposing responses to the presence of a paramagnetic metal center to enhance temperature sensitivity.

**Figure 9:**
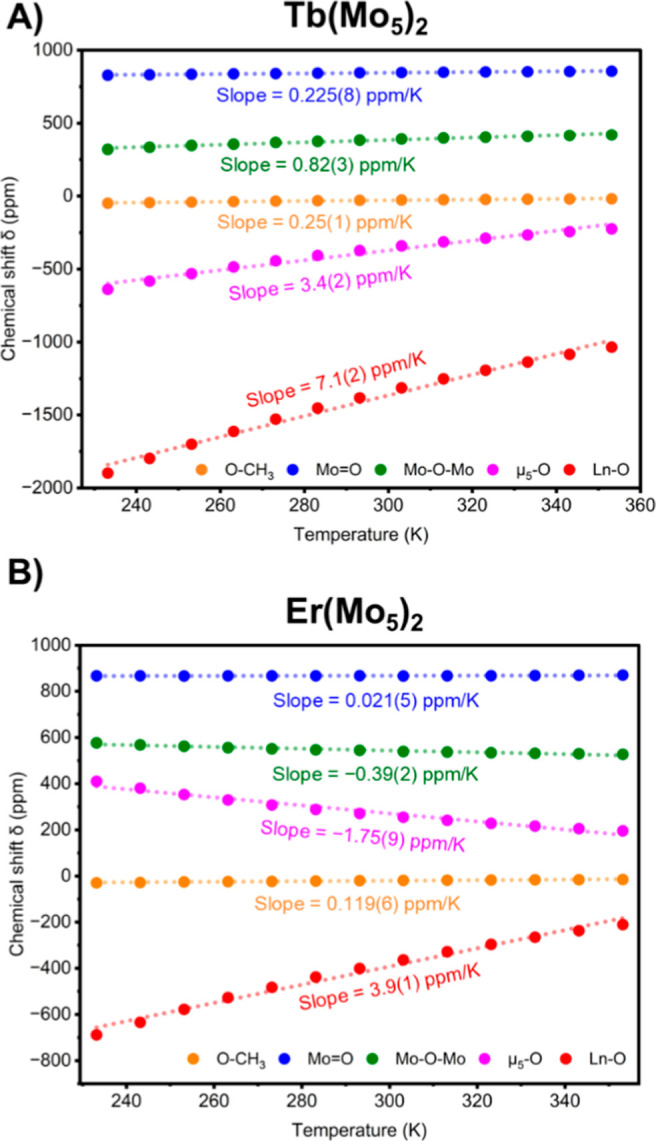
Plots of chemical shift (ppm) vs temperature (K) produced from VT ^1^H/^17^O NMR spectra of **Tb­(Mo**_**5**_ **)**_**2**_ (A) and **Er­(Mo**_**5**_ **)**_**2**_ (B). Spectra obtained in CD_3_CN at −40 to 80 °C.

The changes in chemical shift with temperature for each position are fit linearly to give insights into the approximate change in chemical shift per Kelvin (ppm/K), in line with literature precedent.^60,135–138^ However, It is worth noting that for the more peripheral nuclei (i.e., Mo = ***O***, Mo–***O***–Mo, and -OC ***H***_3_), the LIS is suspected to be dominated by pseudocontact shifting and thus the observed T^–1^ dependency is not expected based on Bleaney’s theory. Instead, T^–2^ dependency is expected based on eqs 4 and 5, a deviation that is well cited in the literature.^60,135–138^ A central assumption in Bleaney’s theory is that crystal-field splitting energies created by parameters *B*_0_^2^ and *B*_2_^2^ are much smaller than *k*T.^121^ This has been shown to be incorrect in many cases and thus experimental temperature dependent behavior is often more accurately modeled by an expansion of Bleaney’s equations to include higher order contributions (i.e., δ = α_1_ *T* ^–1^ + α_2_ *T* ^–2^ + α_3_ *T* ^–3^ + ...).^139,140^ The chemical shifts of the nuclei closest to the Ln center, i.e. μ_5_-***O***’s and Ln-***O***’s, do not vary linearly with temperature over the temperature range studied, and thus the fittings in Figure 9 only serve as a guideline for temperature sensitivity. The difference in temperature dependent behavior between the peripheral nuclei and those close to the Ln could be caused by additional contributions of Fermi contact shifting to the overall LIS, which further complicates the overall temperature dependence. A practical result of this is that the most sensitive nuclei in our systems (i.e., the Tb–***O***–Mo of **Tb­(Mo**_**5**_ **)**_**2**_) display enhanced temperature dependence at lower temperatures (ca. 9.5 ppm/K at −40 °C to −10 °C) at the cost of a diminished response at higher temperatures (ca. 5.3 ppm/K at 50 to 80 °C). In principle, separation of Fermi contact and pseudocontact contributions may be attempted using linearized Bleaney-type treatments (e.g., δ_*ij*_^para^ *T* vs 1/*T*).^141^ However, application of this approach to our systems does not yield consistent linear behavior, particularly for nuclei closest to the Ln center. This reflects the breakdown of the simplifying assumptions of the Bleaney model in systems with large crystal field splitting, where higher-order temperature dependencies become significant.^142^

To put our results in context, the most widely employed Ln-based complexes used in NMR thermometry are derivatives of Ln-DOTA (2,2′,2″,2‴-(1,4,7,10-tetraazacyclododecane-1,4,7,10-tetrayl)­tetraacetic acid) complexes.^60,64,137,138,143–147^ It is important to state that the temperature dependent NMR properties of complexes using a variety of Ln and ligand combinations have been widely studied in aqueous media and in vivo. These complexes tend to rely on ^1^H NMR thermometry, with typical temperature dependences of peripheral protons of 0.004–2.18 ppm/K. These values are smaller than the values obtained for the ^17^O nuclei in our systems and thus the complexes reported here appear competitive from the perspective of sensitivity. However, these previously reported complexes have been developed, in part, for their compatibility with biological systems, not just their temperature dependence. They also show pH dependent chemical shifts and therefore can be considered multifunctional probes. Neither of these considerations is directly relevant to the Ln-based systems developed in this work.

There are also several first–row transition metal complexes reported for their temperature dependent NMR properties.^135,136,148–153^ These complexes are typically prepared, and studied, in nonaqueous solvents and rely on multinuclear NMR handles (^13^C, ^19^F, ^31^P, ^59^Co) and therefore are the most appropriate comparisons to the complexes studied here. The most sensitive NMR thermometers reported are cobalt complexes.^135,136,148–150^ Commercially available Co­(acac)_3_ displays a ^59^Co chemical shift temperature sensitivity of 2.83 ppm/K,^136^ while Co­[CpCo­{PO­(OR)_2_}_3_]_2_ (R = Me, Et, ^i^Pr, and ^*t*^Bu) display record sensitivities of 100–150 ppm/K, dwarfing the values reported here.^154^ This example aside, the ^17^O NMR temperature sensitivity we report for **Ln­(Mo**_**5**_ **)**_**2**_ complexes are competitive with the majority of transition metal base NMR thermometers, while possessing far more independent NMR handles opening the door to self-referencing ratiometric thermometry, where the shift difference between two sites within the same complex encodes absolute temperature without any external calibration standard.^143^ Furthermore, though ^17^O NMR spectroscopy has been applied extensively to paramagnetic systems,^85,86,155–158^ with notable examples examining relaxation rate enhancements^159–161^ and solvent co-ordination,^162^ there are few that examine variable temperature NMR spectroscopy.^162–164^

While the **Tb­(Mo**_**5**_ **)**_**2**_ complex presented here is highly temperature sensitive, the limited solubility of the complex in aqueous solution limits application to biological systems. Instead, future studies focused on other POM systems that are soluble and stable in water at near neutral pH (e.g., K_9_[Ln­(W_5_O_18_)_2_].*n*H_2_O)^86^ could bring the promising ^17^O NMR thermometry behavior presented here into biological systems.

## Conclusions

Overall, we have presented a unified, robust, synthetic approach for the synthesis of a complete series of Ln polyoxoalkoxide sandwich-type complexes **Ln­(Mo**_**5**_ **)**_**2**_ (except *Pm*), with our approach being both high yielding and user-friendly. Extensive characterization reveals that the vibrational and redox properties of the complexes are essentially unchanged when changing Ln. Conversely, UV–vis spectroscopy, NMR spectroscopy, and SCXRD experiments reveal that optical, magnetic, and structural properties are highly Ln dependent. Specifically, presence of large, noncoordinating TBA cations leads to a complete series of 14 isostructural **Ln­(Mo**_**5**_ **)**_**2**_ complexes where the only major structural changes are driven by changes in the ionic radius of the central Ln^3+^ ion. Smaller Ln’s possess shorter Ln–O bond distances which drive a smaller separation between the two {Mo_5_} ligands. This leads to more ideal square antiprismatic geometries for late Ln’s when compared to early Ln’s. Drastically larger cations (Sr^II^ or Ba^II^) have larger {Mo_5_}-to-{Mo_5_} separations and instead adopt a cubic geometry at the central metal ion. The isostructural nature of the series of **Ln­(Mo**_**5**_ **)**_**2**_ complexes provides a persistent coordination environment for interrogating the Ln dependent NMR properties of the series. ^1^H and ^17^O NMR experiments reveal chemical shifts are heavily dependent on both the position of the NMR active nucleus with respect to the Ln center and the nature of the Ln present, in line with previous reports. Investigations into the variable temperature behavior show that nuclei close to the Ln center display highly temperature sensitive chemical shifts, with the specific direction and magnitude of the observed LIS varying with the Ln. The temperature sensitivities of specific nuclei in the ^17^O NMR spectra of **Tb­(Mo**_**5**_ **)**_**2**_ and **Er­(Mo**_**5**_ **)**_**2**_ surpass many literature complexes, opening the door to the future studies investigating the use of LnPOM species in magnetic resonance thermometry.

## Supplementary Material



## Notes

Additional spectroscopic, crystallographic, and voltametric data (PDF). Crystallographic data for **Sr­(Mo**_**5**_ **)**_**2**_, **Y­(Mo**_**5**_ **)**_**2**_, **Tm­(Mo**_**5**_ **)**_**2**_, **Yb­(Mo**_**5**_ **)**_**2**_, and **Lu­(Mo**_**5**_ **)**_**2**_ have been deposited at the CCDC under 2538977–2538981 (PDF)
