# Supplementary material for: Lanthanide-Centered Polyoxoalkoxide Complexes Provide a Platform for Systematic Room- and Variable-Temperature Nuclear Magnetic Resonance Studies
Source: Inorg Chem. 2026 May 21;65(22):12557–73. doi: 10.1021/acs.inorgchem.6c01439 (PMC13250993; doi:10.1021/acs.inorgchem.6c01439)
Supplement: Supplementary file 1 [file ic6c01439_si_001.pdf]

Electronic Supporting Information

Lanthanide-centered Polyoxoalkoxide Complexes Provide a Platform for Systematic Room- and Variable-Temperature Nuclear Magnetic Resonance Studies

Dominic Shiels‡*, Nadeeshan Gunarathna‡, William W. Brennessel, and Ellen M. Matson*

‡Authors contributed equally to this work.

Department of Chemistry, University of Rochester, Rochester, NY 14627, USA

Corresponding Author E-mail

Dominic Shiels: dshiels@ur.rochester.edu

Ellen M. Matson: matson@chem.rochester.edu

Content

S1. ^{1}H NMR Spectra S3
S2. ^{17}O NMR Spectra S10
S3. Relaxation Time Measurements S17
S4. Variable Temperature NMR S18
S5. UV-Vis-NIR Spectra S20
S6. Electrochemistry S27
S7. Single Crystal X-ray Diffraction S34
S8. IR Spectra S52

S1. ^{1}H NMR Spectra

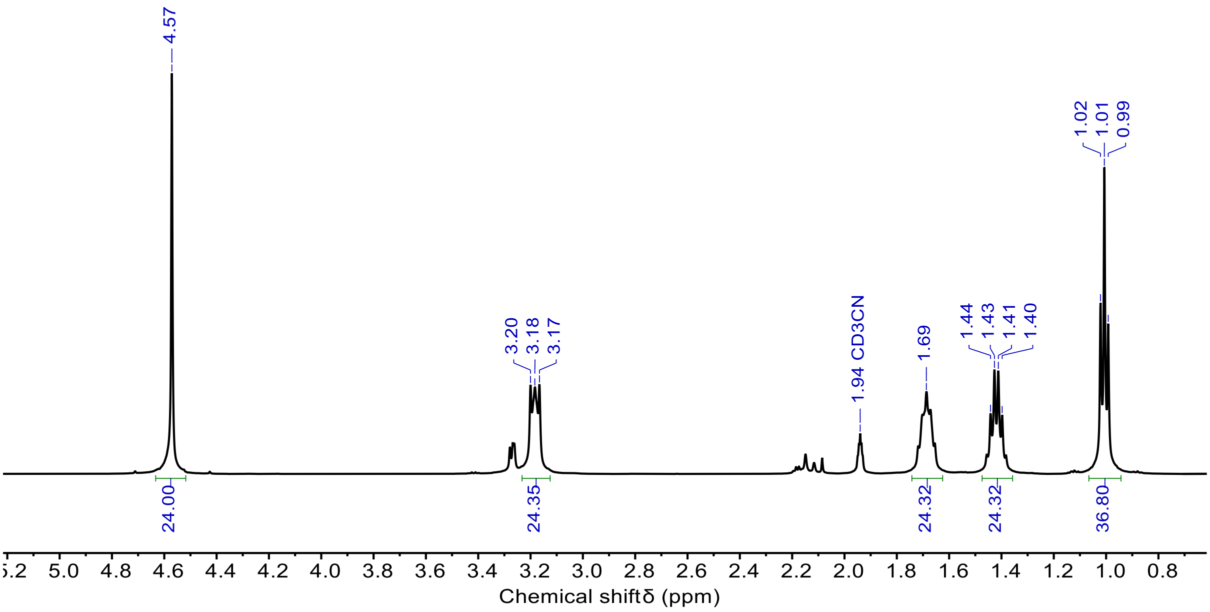

Figure S1.^{1}H NMR spectrum (500 MHz) of $(TBA)_3[La\{Mo_5O_{13}(OMe)_4NO\}_2]$ (**La(Mo$_5$)$_2$**) in CD_3CN.

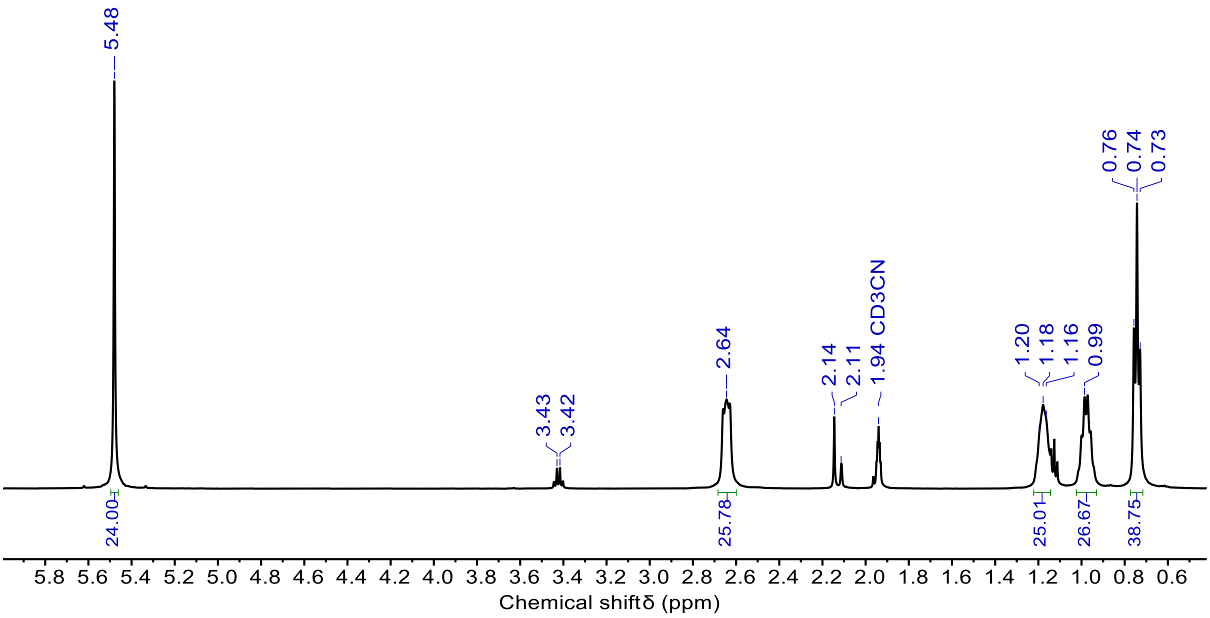

Figure S2.^{1}H NMR spectrum (500 MHz) of $(TBA)_3[Pr\{Mo_5O_{13}(OMe)_4NO\}_2]$ (**Pr(Mo$_5$)$_2$**) in CD_3CN. Residual solvent signals at 3.4 ppm and 2.1 ppm identified as Et_2O and H_2O.

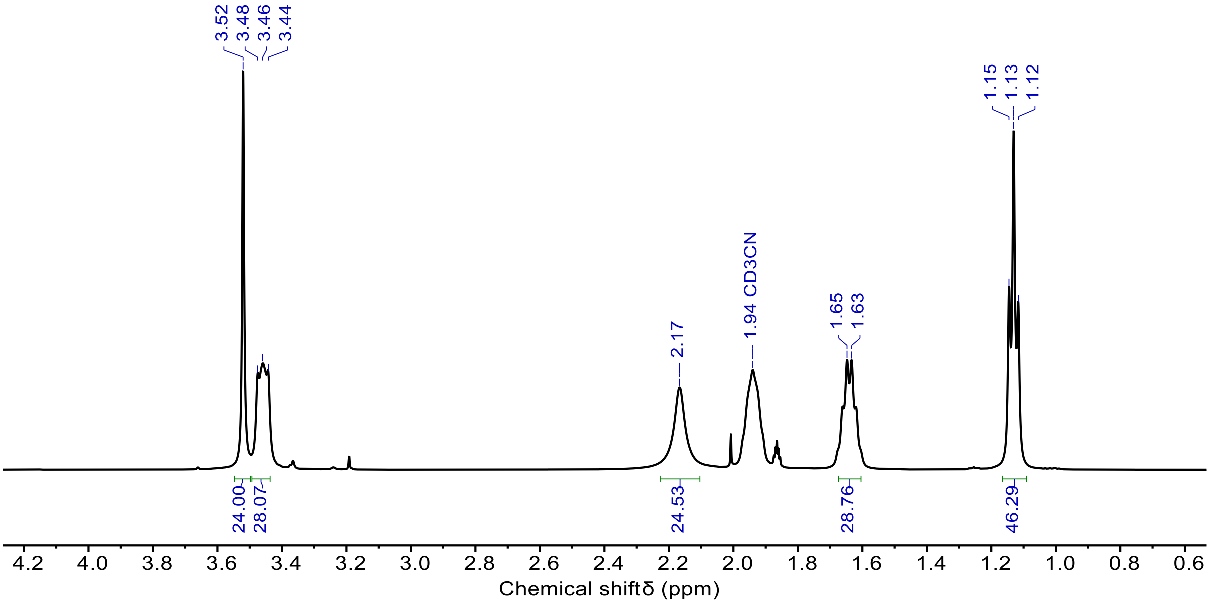

Figure S3. ^{1}H NMR spectrum (500 MHz) of $(TBA)_3[Nd\{Mo_5O_{13}(OMe)_4NO\}_2]$ (**$Nd(Mo_5)_2$**) in CD_3CN.

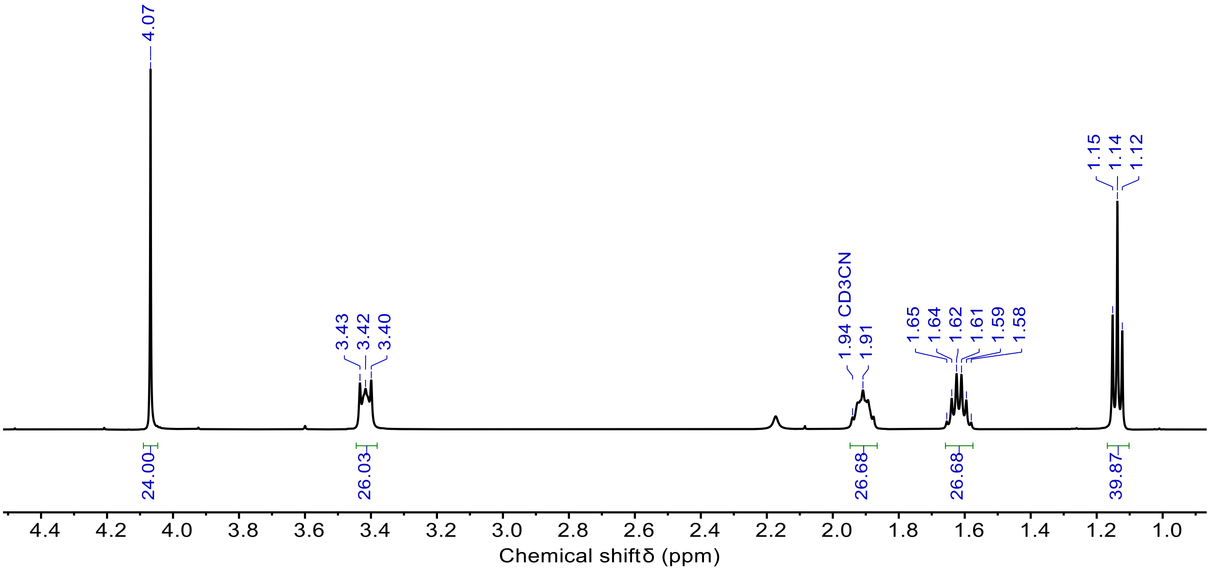

Figure S4. ^{1}H NMR spectrum (500 MHz) of $(TBA)_3[Sm\{Mo_5O_{13}(OMe)_4NO\}_2]$ (**$Sm(Mo_5)_2$**) in CD_3CN.

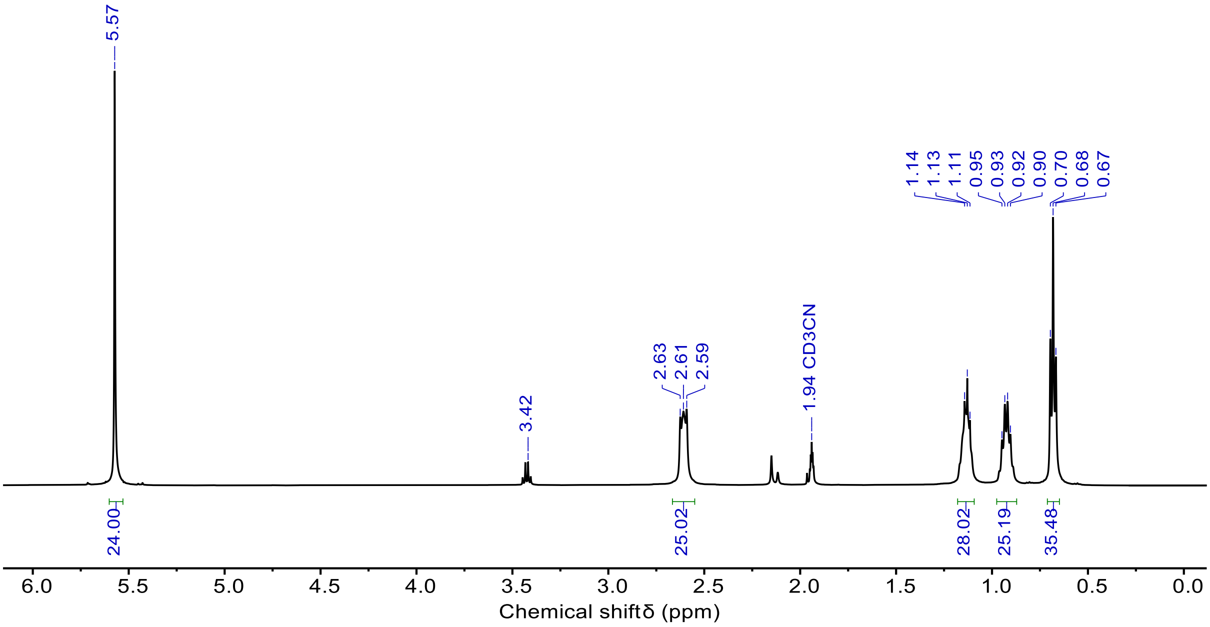

Figure S5.^{1}H NMR spectrum (500 MHz) of $(TBA)_3[Eu\{Mo_5O_{13}(OMe)_4NO\}_2]$ (**Eu(Mo$_5$)$_2$**) in CD_3CN. Residual solvent signals at 3.4 ppm identified as Et_2O.

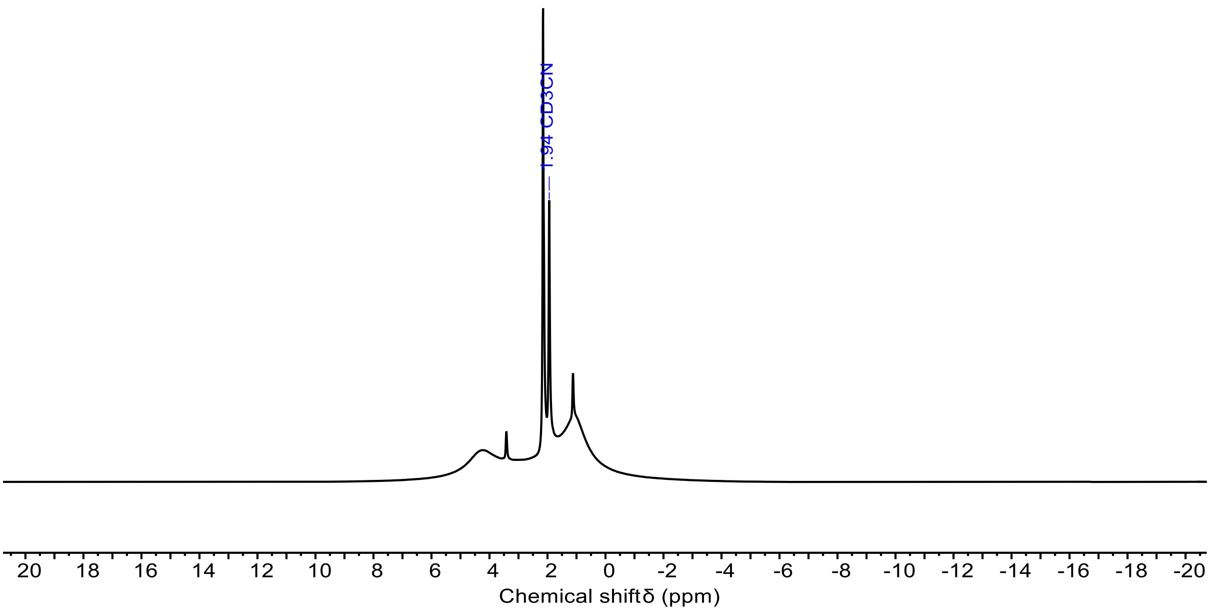

Figure S6.^{1}H NMR spectrum (500 MHz) of $(TBA)_3[Gd\{Mo_5O_{13}(OMe)_4NO\}_2]$ (**Gd(Mo$_5$)$_2$**) in CD_3CN.

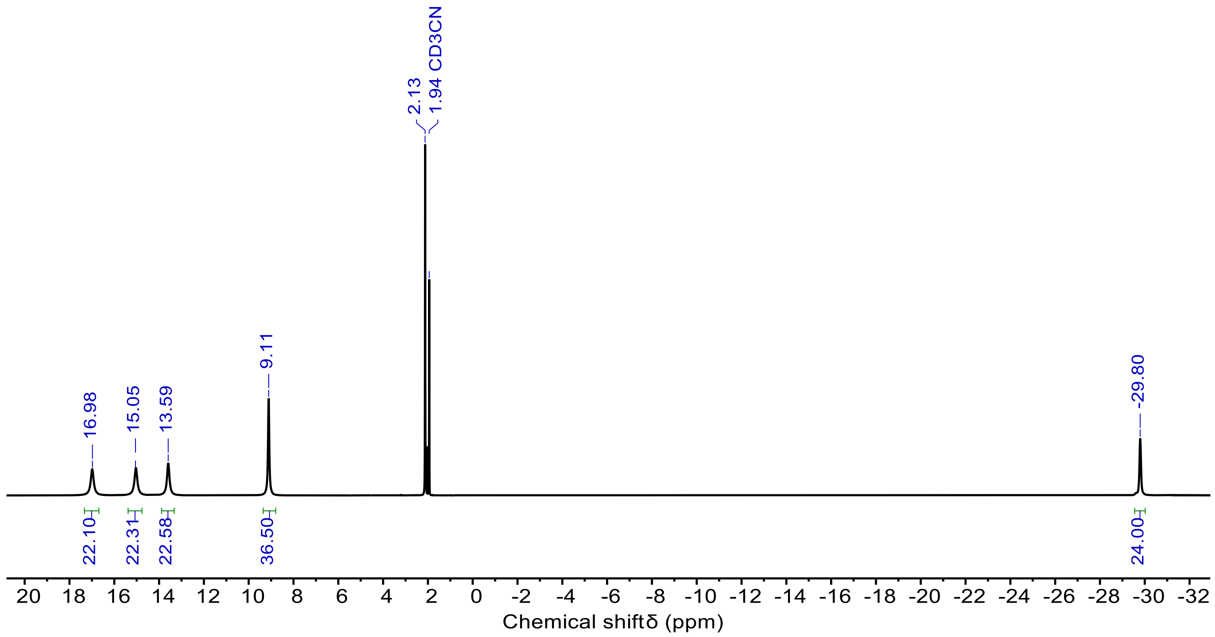

Figure S7.^{1}H NMR spectrum (500 MHz) of $(TBA)_3[Tb\{Mo_5O_{13}(OMe)_4NO\}_2]$ (**Tb(Mo$_5$)$_2$**) in CD_3CN.

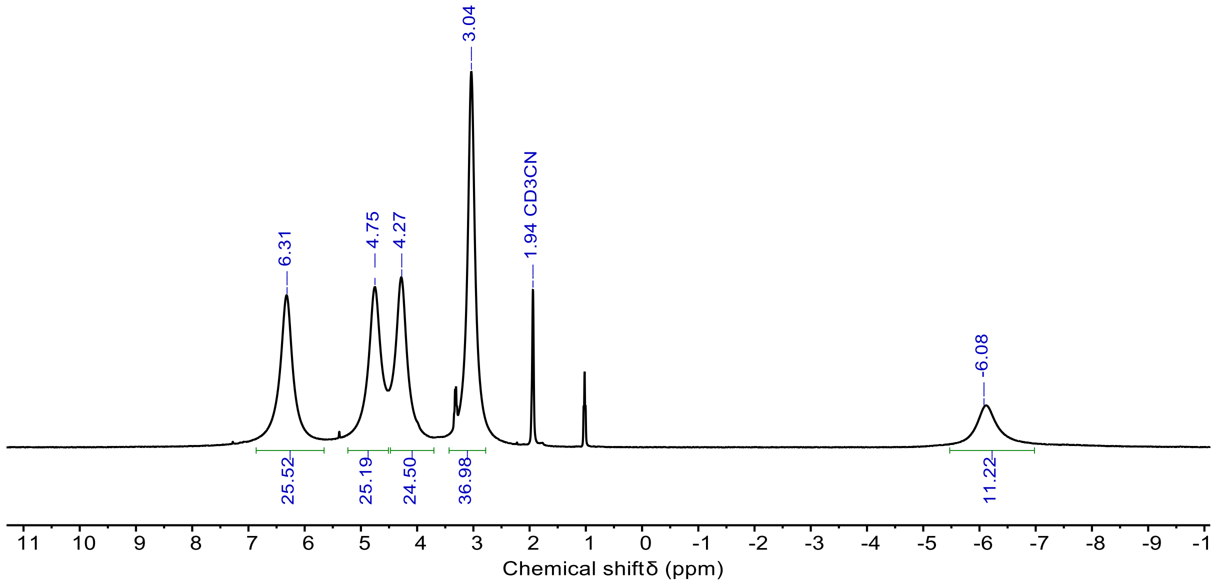

Figure S8.^{1}H NMR spectrum (500 MHz) of $(TBA)_3[Dy\{Mo_5O_{13}(OMe)_4NO\}_2]$ (**Dy(Mo$_5$)$_2$**) in CD_3CN. Residual solvent signal at 1.12 ppm identified as DCM.

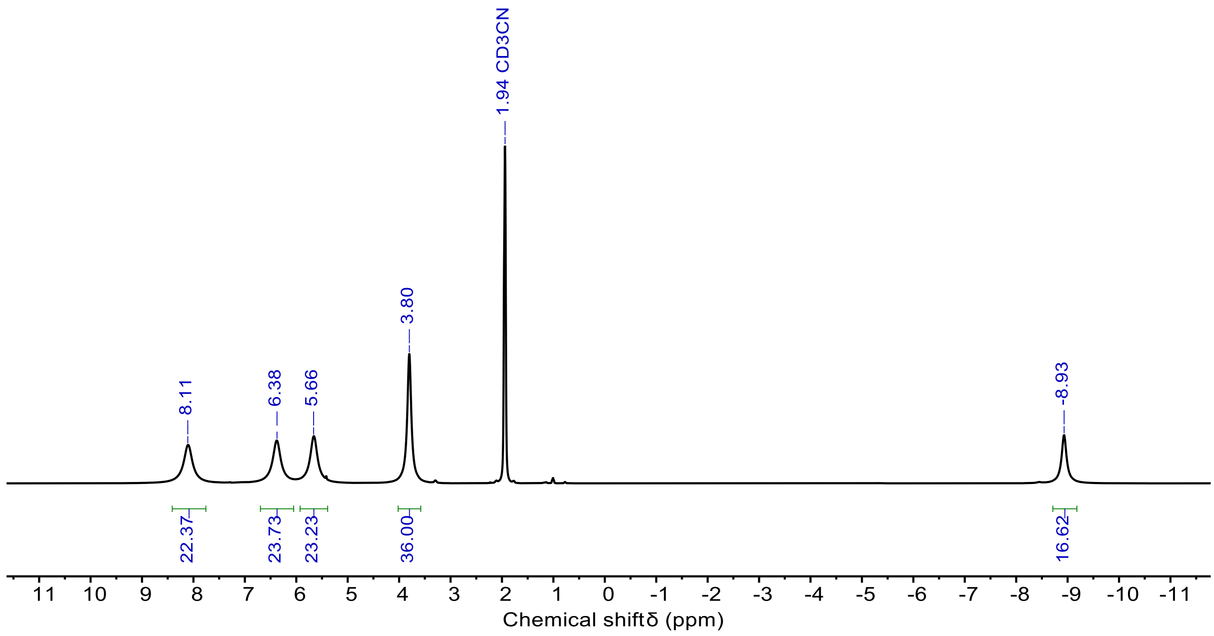

Figure S9.^{1}H NMR spectrum (500 MHz) of $(TBA)_3[Ho\{Mo_5O_{13}(OMe)_4NO\}_2]$ (**$Ho(Mo_5)_2$**) in CD_3CN.

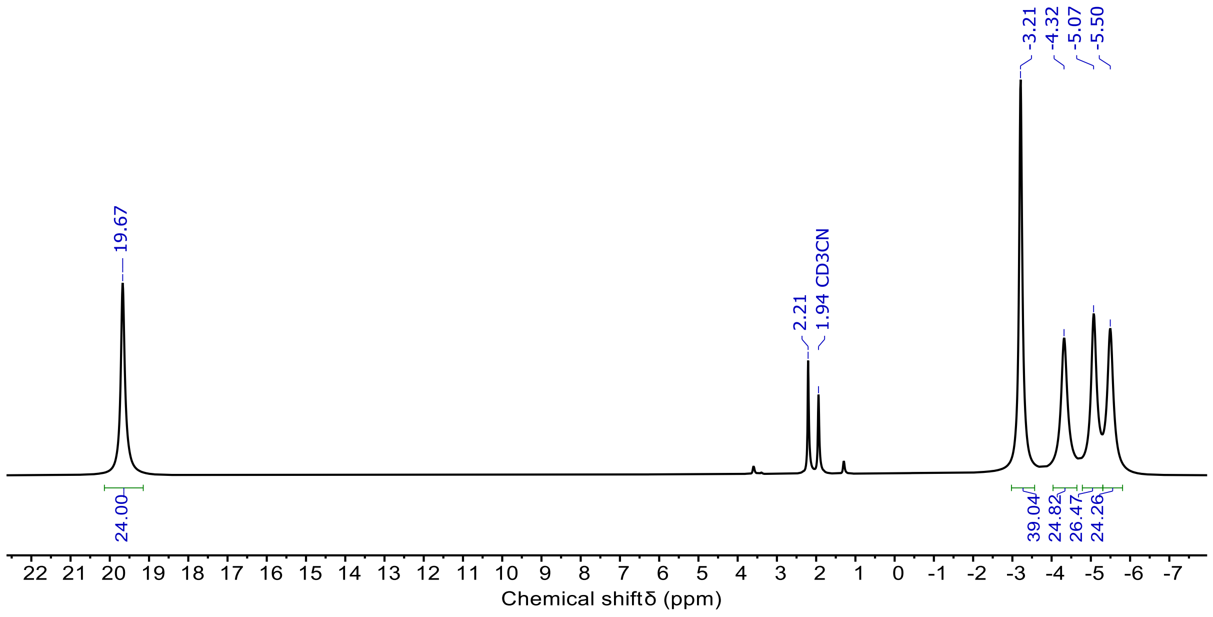

Figure S10.^{1}H NMR spectrum (500 MHz) of $(TBA)_3[Er\{Mo_5O_{13}(OMe)_4NO\}_2]$ (**$Er(Mo_5)_2$**) in CD_3CN.

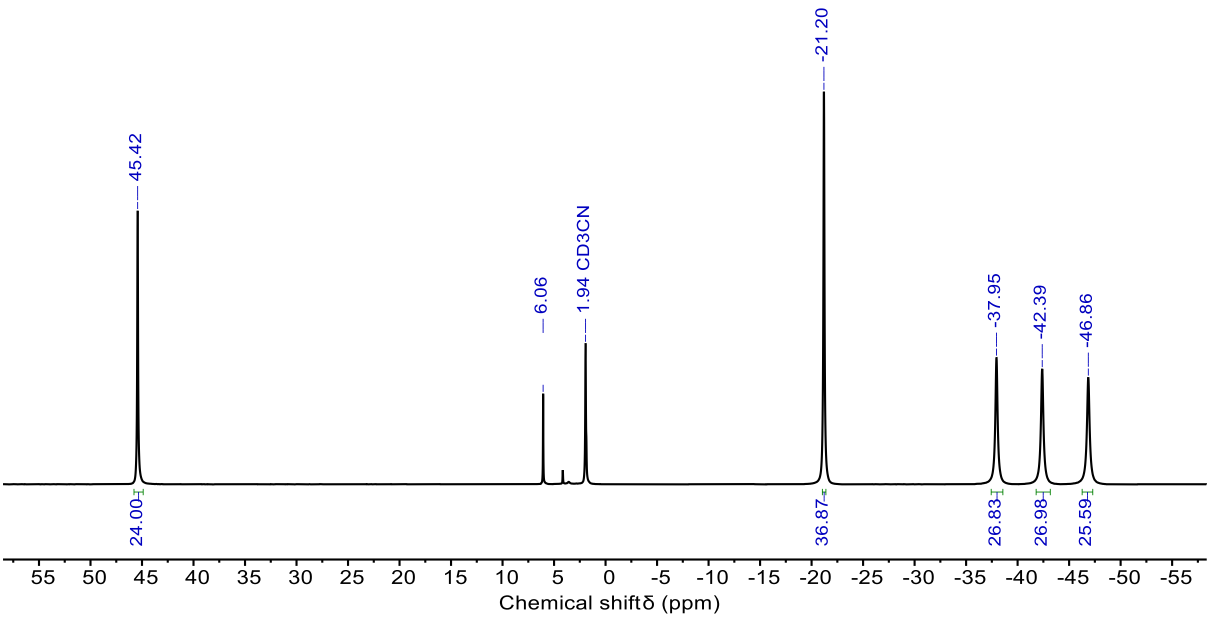

Figure S11.^{1}H NMR spectrum (500 MHz) of $(TBA)_3[Tm\{Mo_5O_{13}(OMe)_4NO\}_2]$ (**Tm(Mo$_5$)$_2$**) in CD_3CN. Residual signal at 6.06 ppm was unidentified.

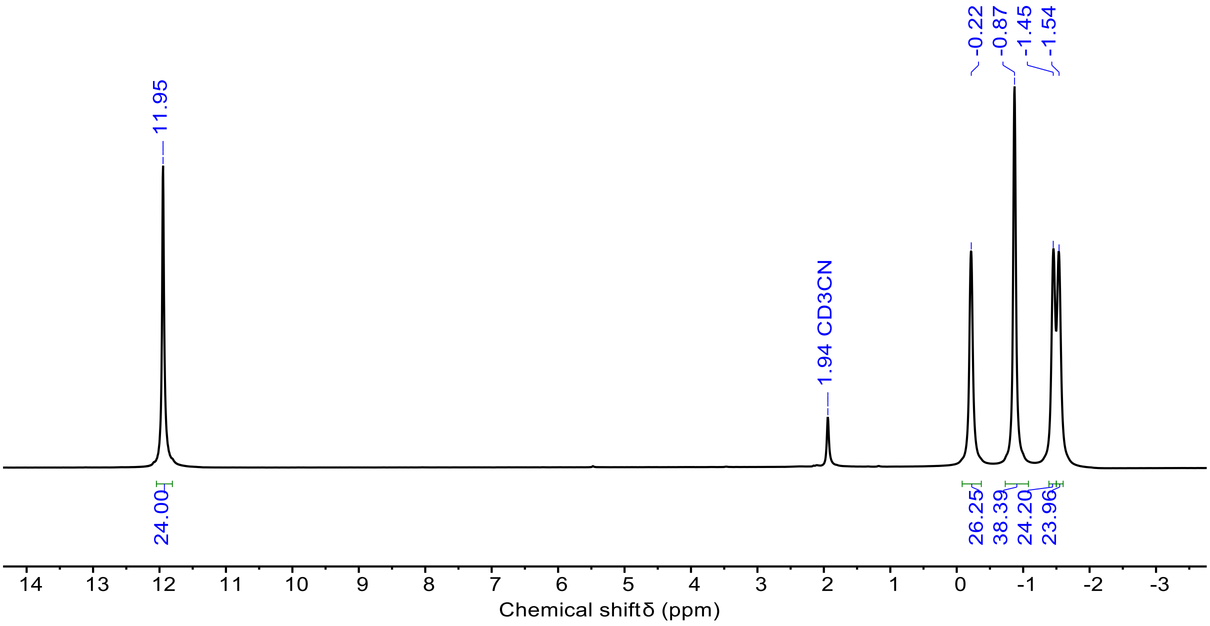

Figure S12.^{1}H NMR spectrum (500 MHz) of $(TBA)_3[Yb\{Mo_5O_{13}(OMe)_4NO\}_2]$ (**Yb(Mo$_5$)$_2$**) in CD_3CN.

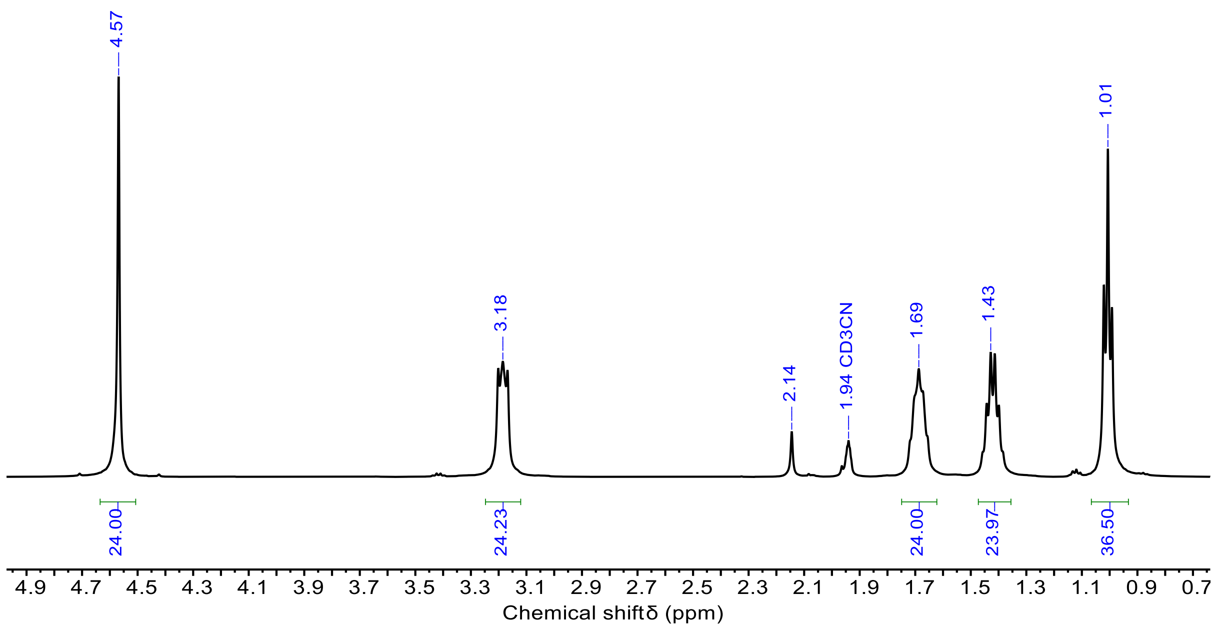

Figure S13.^{1}H NMR spectrum (500 MHz) of $(TBA)_3[Lu\{Mo_5O_{13}(OMe)_4NO\}_2]$ (**$Lu(Mo_5)_2$**) in CD_3CN. Residual solvent signals at 2.14 ppm identified as H_2O.

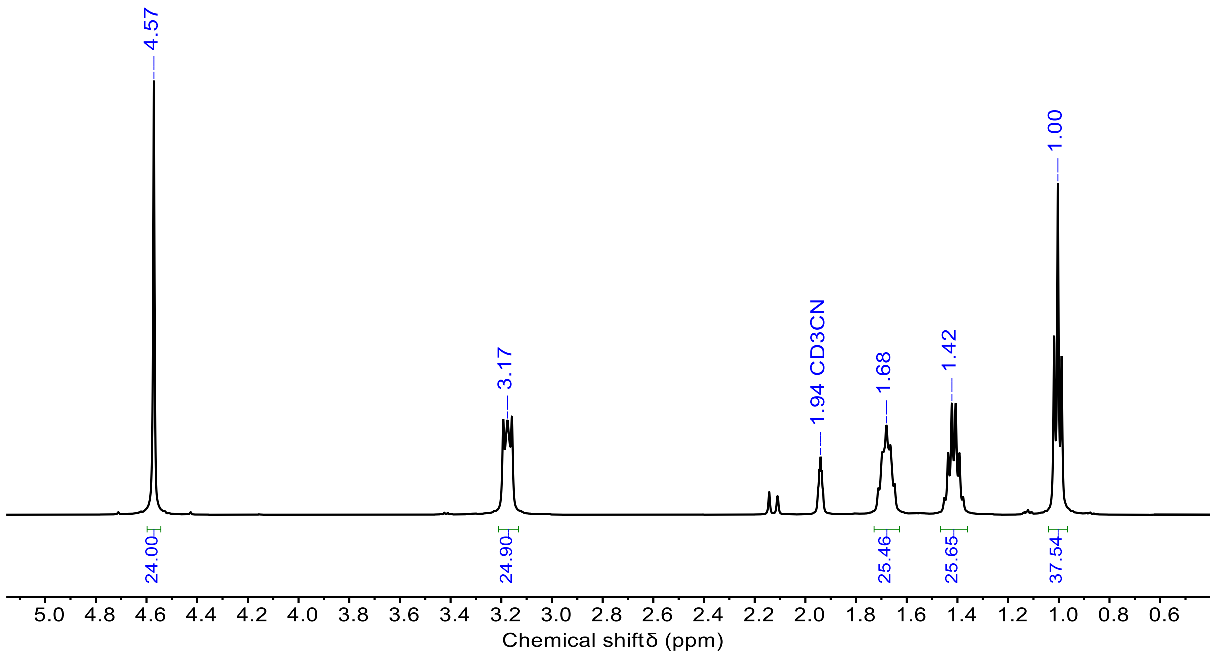

Figure S14.^{1}H NMR spectrum (500 MHz) of $(TBA)_3[Y\{Mo_5O_{13}(OMe)_4NO\}_2]$ (**$Y(Mo_5)_2$**) in CD_3CN.

S2. ^{17}O NMR Spectra

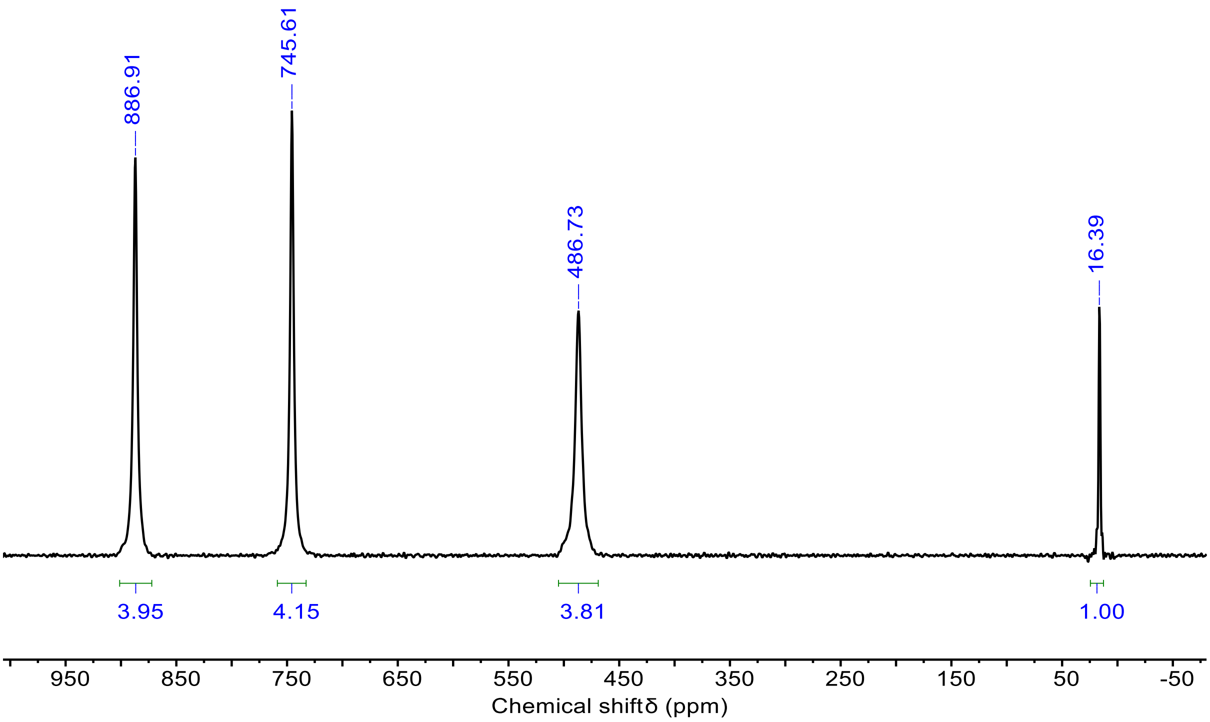

Figure S15.^{17}O NMR spectrum (54.3 MHz) of $(TBA)_3[La\{Mo_5O_{13}(OMe)_4NO\}_2]$ (**La(Mo$_5$)$_2$**) in CD_3CN.

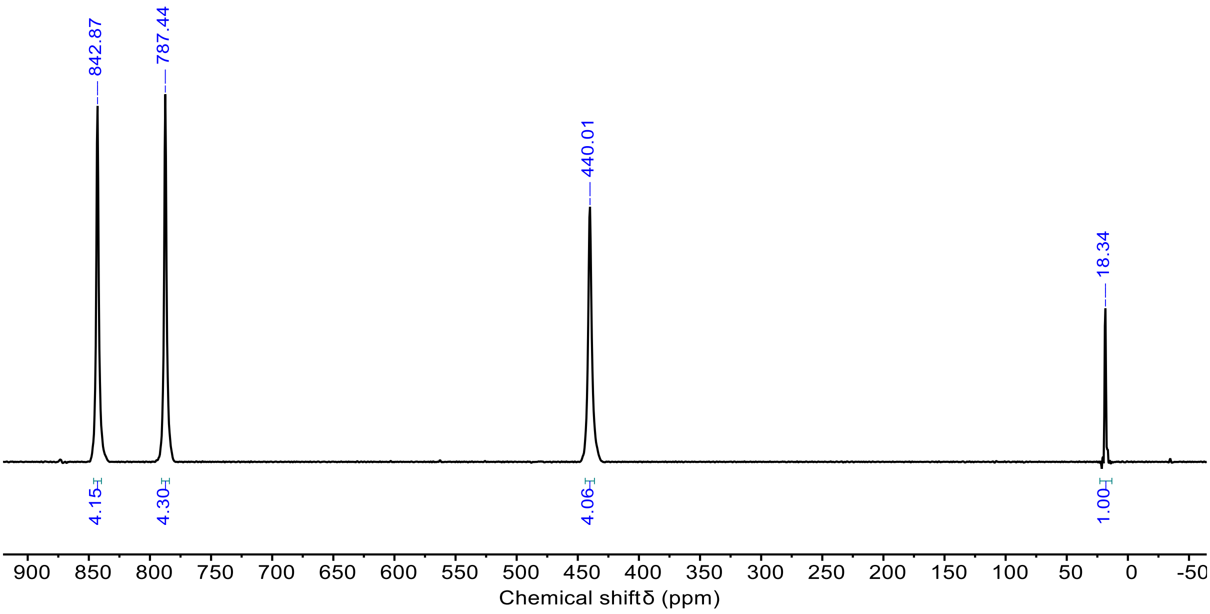

Figure S16.^{17}O NMR spectrum (54.3 MHz) of $(TBA)_3[Pr\{Mo_5O_{13}(OMe)_4NO\}_2]$ (**Pr(Mo$_5$)$_2$**) in CD_3CN.

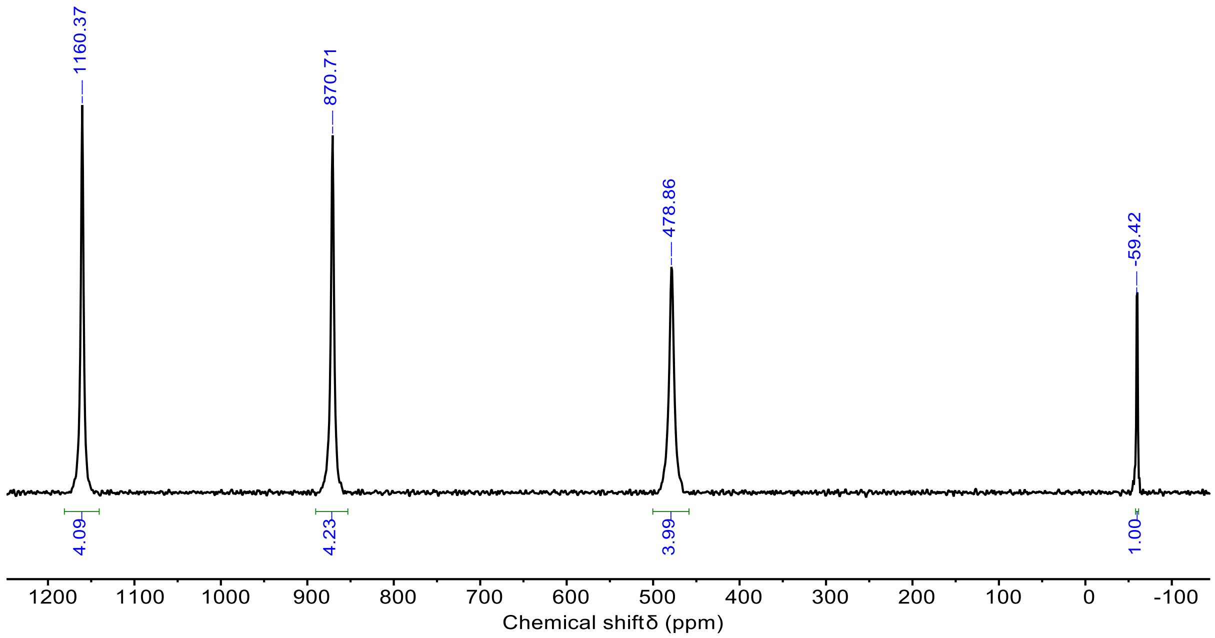

Figure S17.^{17}O NMR spectrum (54.3 MHz) of $(TBA)_3[Nd\{Mo_5O_{13}(OMe)_4NO\}_2]$ (**Nd(Mo5)2**) in CD_3CN.

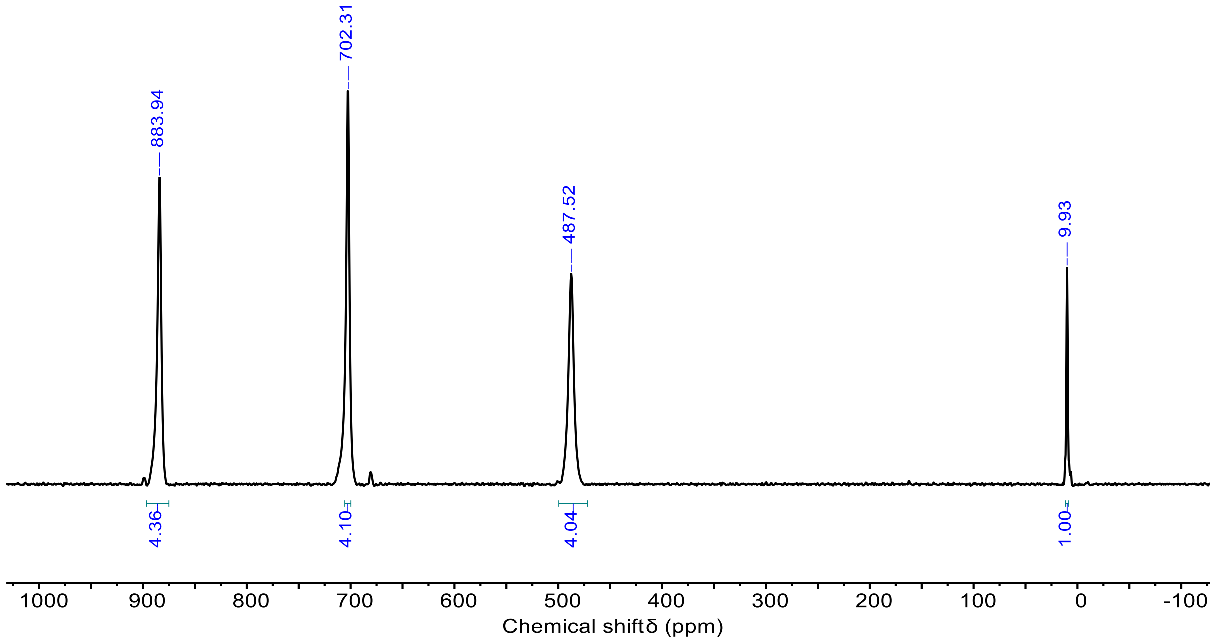

Figure S18.^{17}O NMR spectrum (54.3 MHz) of $(TBA)_3[Sm\{Mo_5O_{13}(OMe)_4NO\}_2]$ (**Sm(Mo5)2**) in CD_3CN.

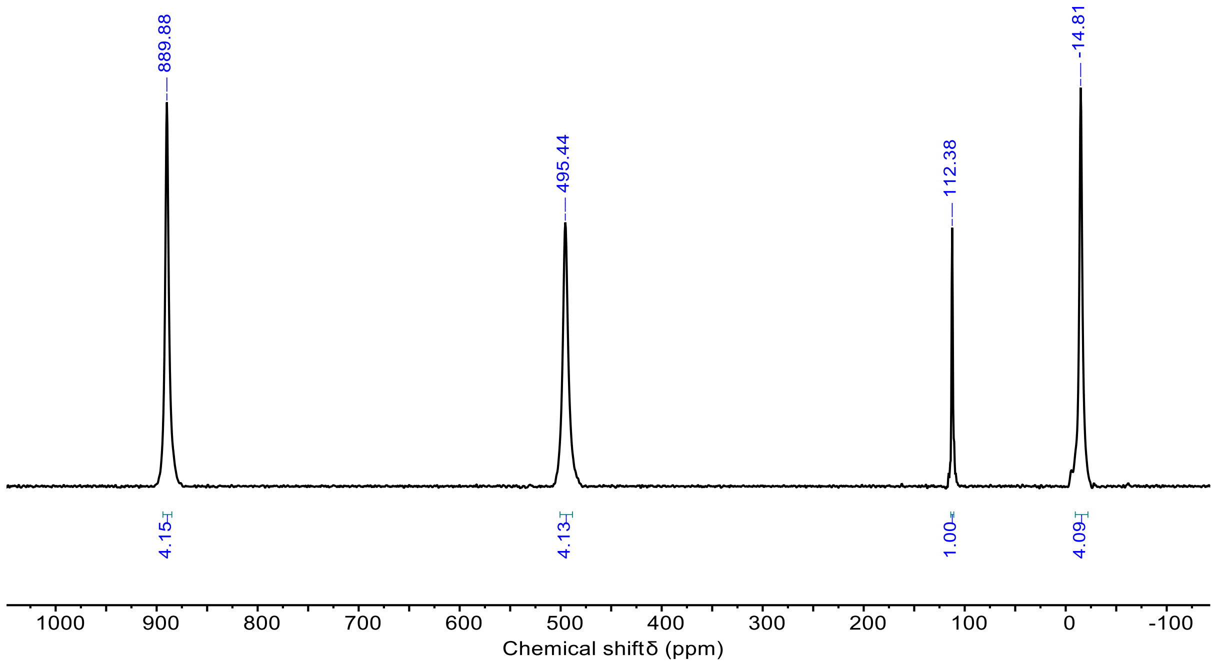

Figure S19.^{17}O NMR spectrum (54.3 MHz) of $(TBA)_3[Eu\{Mo_5O_{13}(OMe)_4NO\}_2]$ (**Eu(Mo$_5$)$_2$**) in CD_3CN.

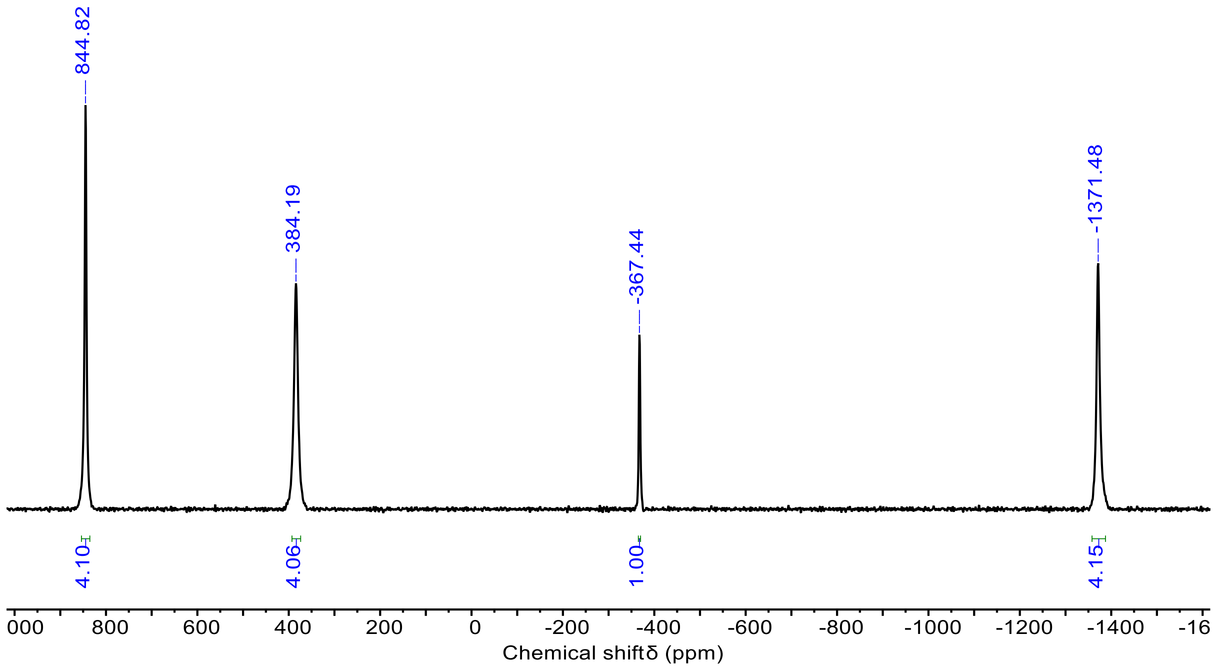

Figure S20.^{17}O NMR spectrum (54.3 MHz) of $(TBA)_3[Tb\{Mo_5O_{13}(OMe)_4NO\}_2]$ (**Tb(Mo$_5$)$_2$**) in CD_3CN.

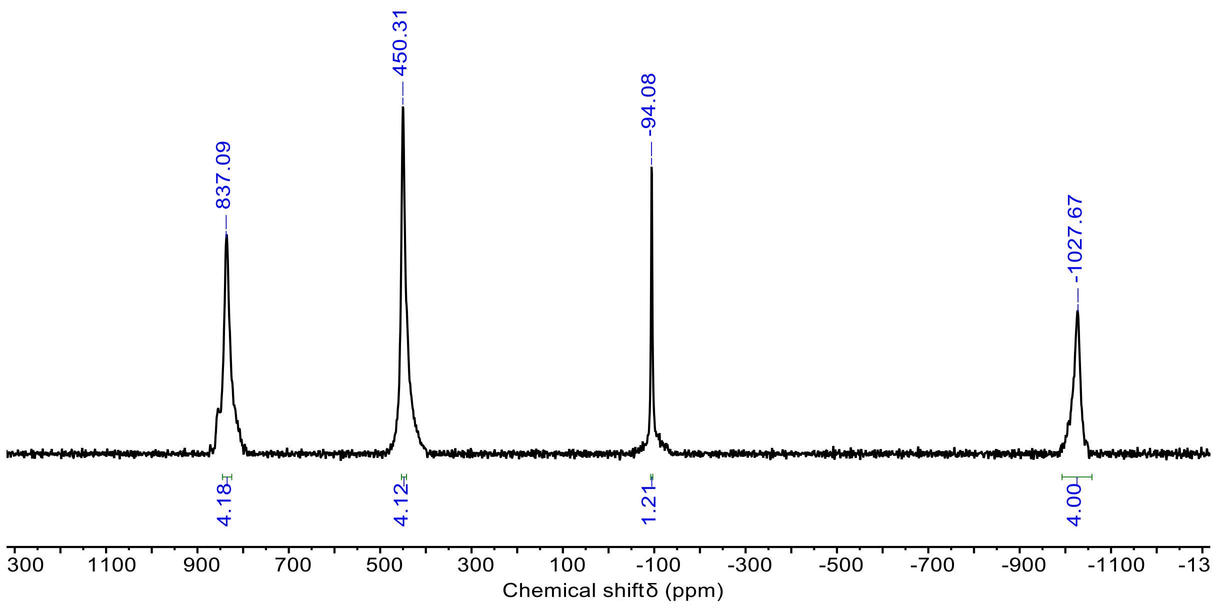

Figure S21.^{17}O NMR spectrum (54.3 MHz) of $(TBA)_3[Dy\{Mo_5O_{13}(OMe)_4NO\}_2]$ (**Dy(Mo$_5$)$_2$**) in CD_3CN.

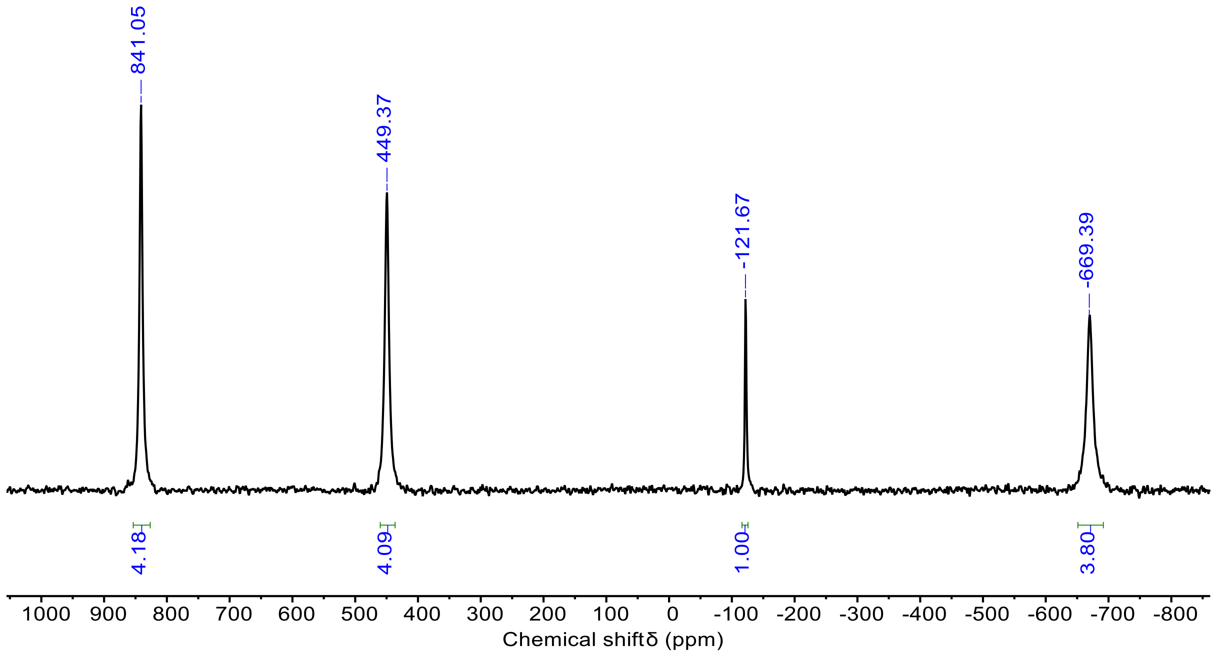

Figure S22.^{17}O NMR spectrum (54.3 MHz) of $(TBA)_3[Ho\{Mo_5O_{13}(OMe)_4NO\}_2]$ (**Ho(Mo$_5$)$_2$**) in CD_3CN.

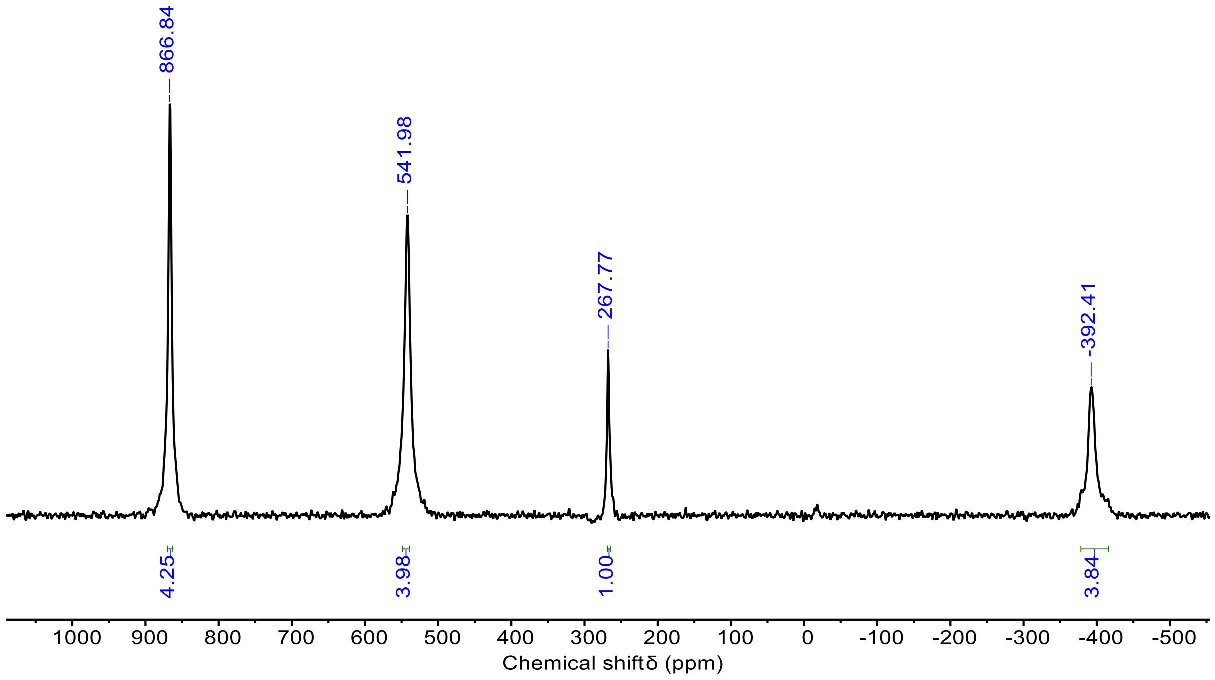

Figure S23.^{17}O NMR spectrum (54.3 MHz) of $(TBA)_3[Er\{Mo_5O_{13}(OMe)_4NO\}_2]$ (**Er(Mo$_5$)$_2$**) in CD_3CN.

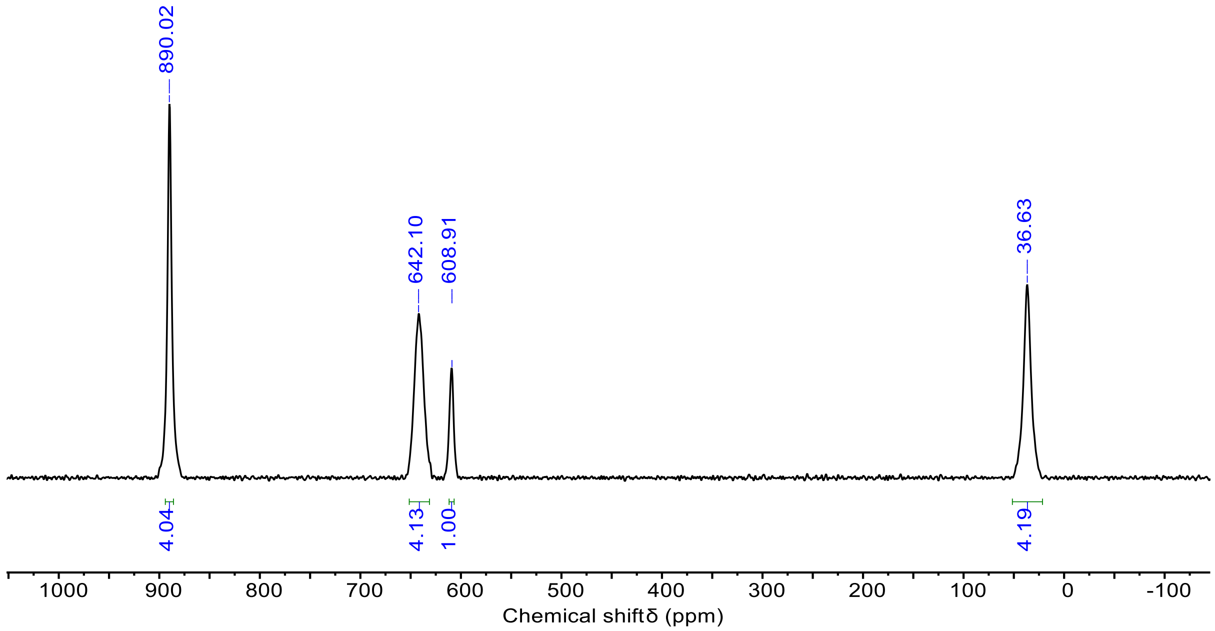

Figure S24.^{17}O NMR spectrum (54.3 MHz) of $(TBA)_3[Tm\{Mo_5O_{13}(OMe)_4NO\}_2]$ (**Tm(Mo$_5$)$_2$**) in CD_3CN.

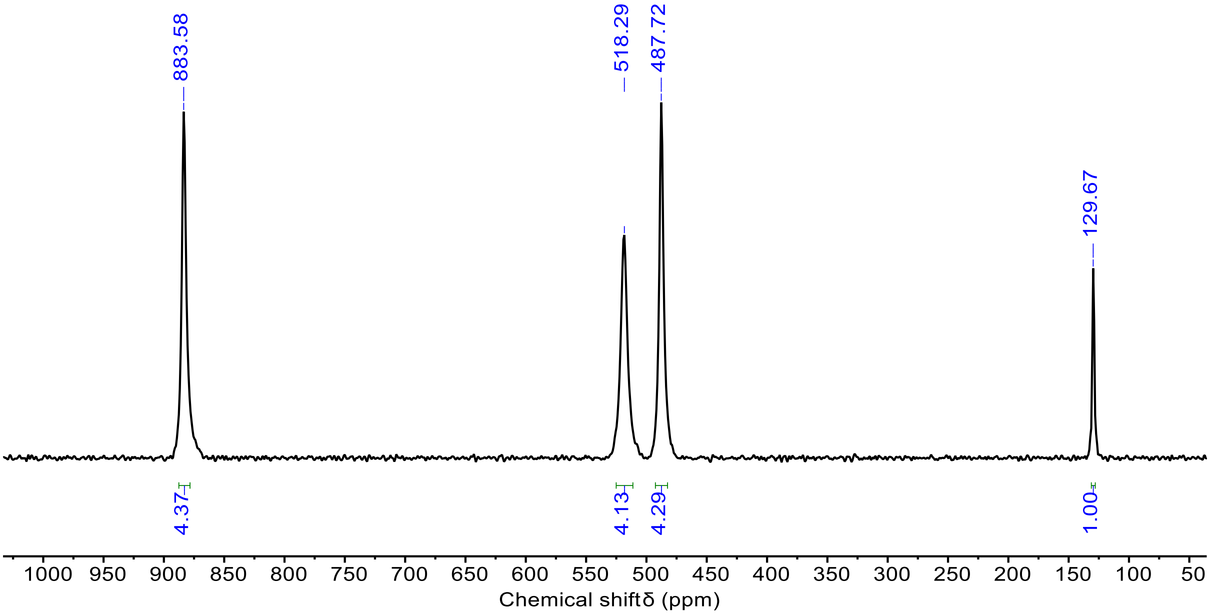

Figure S25. ^{17}O NMR spectrum (54.3 MHz) of $(TBA)_3[Yb\{Mo_5O_{13}(OMe)_4NO\}_2]$ (**Yb(Mo$_5$)$_2$**) in CD_3CN.

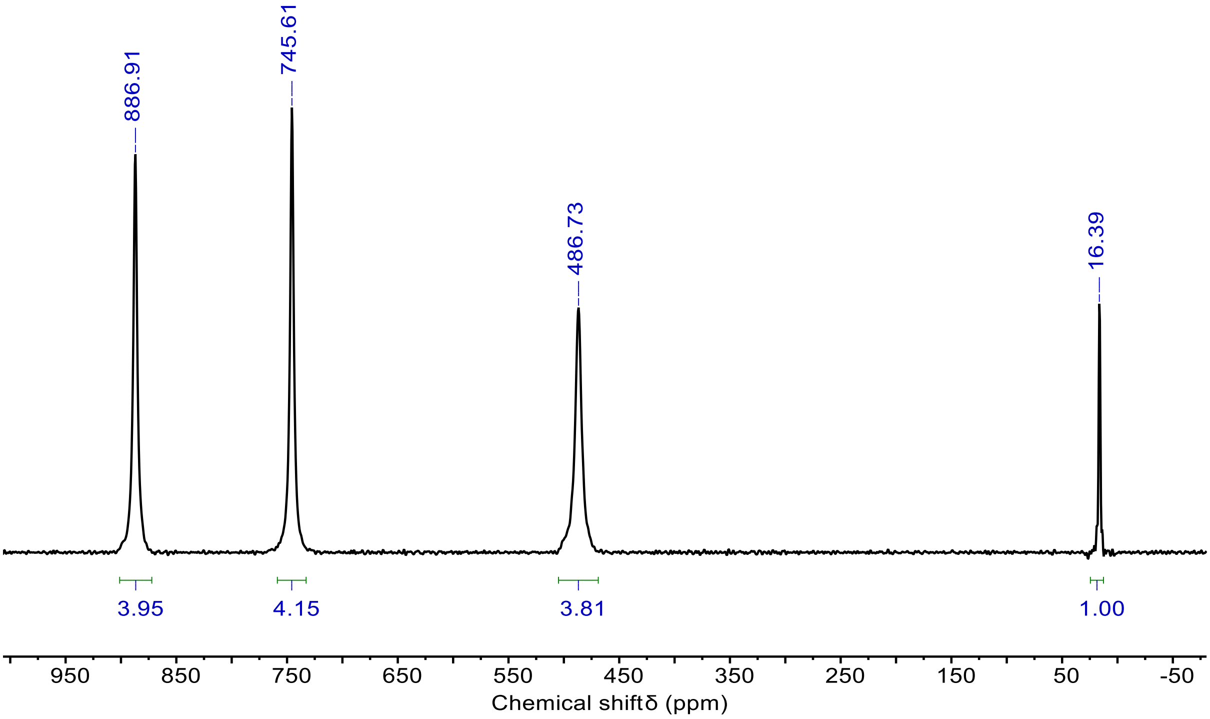

Figure S26. ^{17}O NMR spectrum (54.3 MHz) of $(TBA)_3[Lu\{Mo_5O_{13}(OMe)_4NO\}_2]$ (**Lu(Mo$_5$)$_2$**) in CD_3CN.

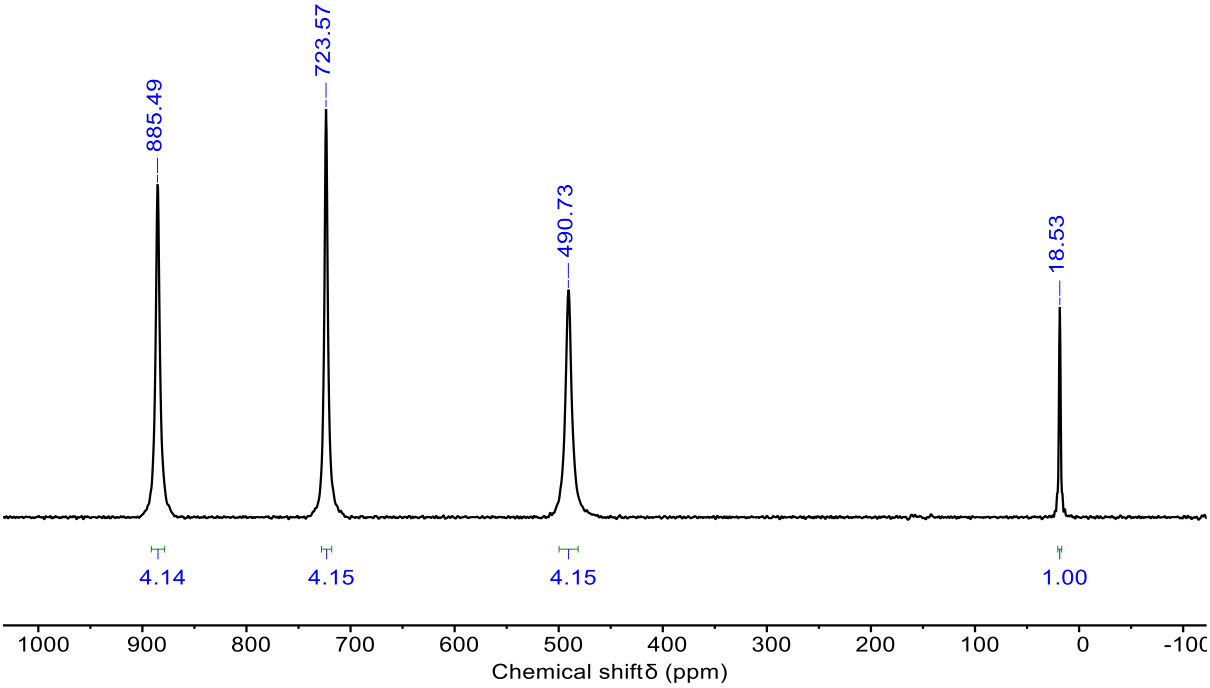

Figure S27.^{17}O NMR spectrum (54.3 MHz) of $(TBA)_3[Y\{Mo_5O_{13}(OMe)_4NO\}_2]$ (**Y(Mo$_5$)$_2$**) in CD_3CN.

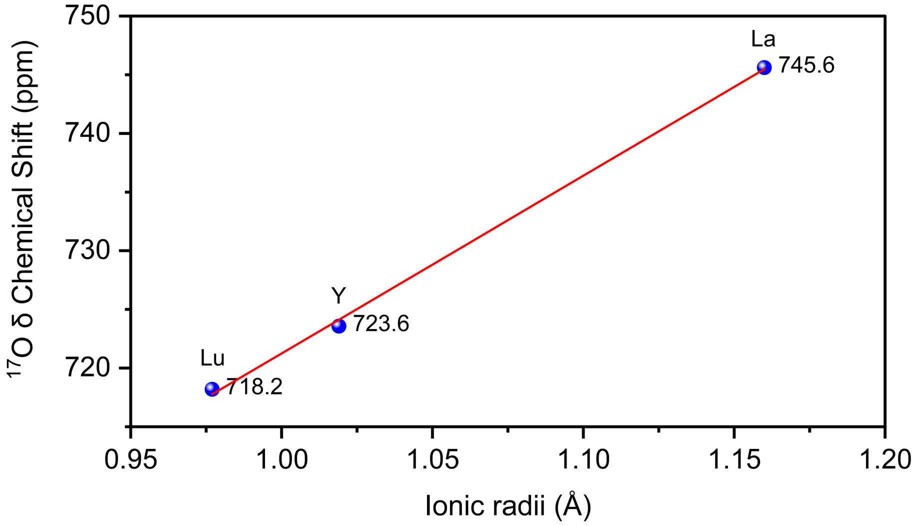

Figure S28.[17]O chemical shift of M-***O***-Mo bridges vs. ionic radii of **La(Mo$_5$)$_2$**, **Y(Mo$_5$)$_2$**, and **Lu(Mo$_5$)$_2$**.

S3. Relaxation Time Measurements

Table S1: Longitudinal relaxation (T_1) time and Transverse relaxation (T_2) times of **$Ln(Mo_5)_2$** compounds recorded in CD_3CN and CD_2Cl_2 at room temperature.

Ln	Electron configuration	T_1 (s) in CD_3CN	T_2 (ms) in CD_3CN	T_1 (s) in CD_2Cl_2	T_2 (ms) in CD_2Cl_2
**La^{3+}**	$4f^0$	1.119	1003	1.108	853
**Ce^{3+}**	$4f^1$	0.784	0.543	0.751	0.543
**Pr^{3+}**	$4f^2$	0.423	0.256	0.418	0.247
**Nd^{3+}**	$4f^3$	0.314	0.154	0.292	0.217
**Sm^{3+}**	$4f^5$	1.035	0.819	1.061	0.833
**Eu^{3+}**	$4f^6$	0.690	0.441	0.653	0.294
**Tb^{3+}**	$4f^8$	0.015	0.021	0.015	0.031
**Dy^{3+}**	$4f^9$	0.012	1.035	0.011	1.029
**Ho^{3+}**	$4f^{10}$	0.009	2.235	0.008	2.149
**Er^{3+}**	$4f^{11}$	0.010	3.551	0.008	3.527
**Tm^{3+}**	$4f^{12}$	0.039	0.257	0.014	0.086
**Yb^{3+}**	$4f^{13}$	0.111	26.002	0.123	84.689
**Lu^{3+}**	$4f^{14}$	1.150	1044	1.140	849

S4. Variable Temperature NMR

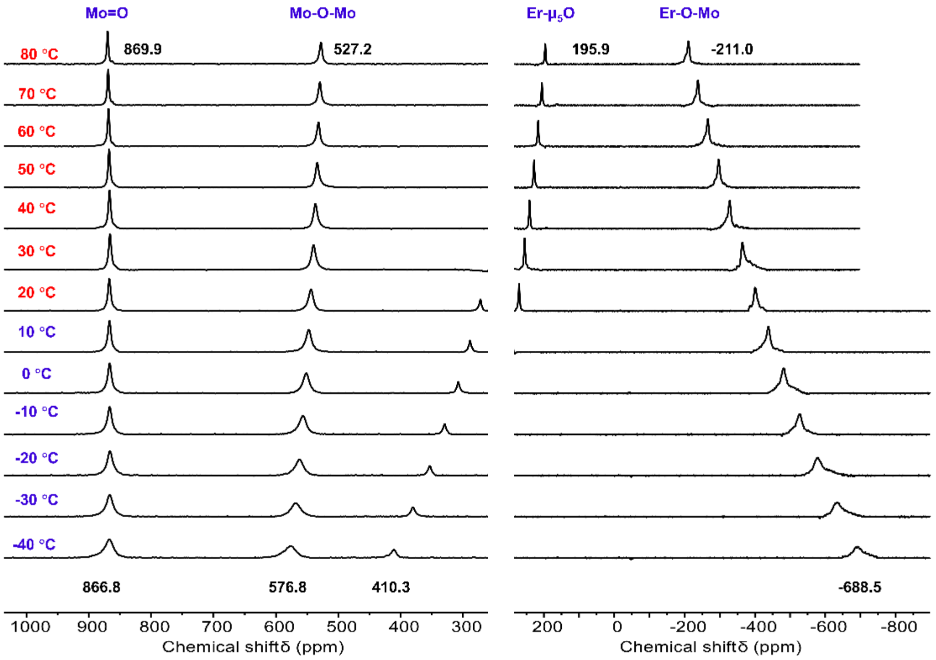

Figure S29. Variable Temperature ^{17}O NMR spectra (54.3 MHz) of $(TBA)_3[Er\{Mo_5O_{13}(OMe)_4NO\}_2]$ (**Er(Mo5)2**). Spectra obtained in CD_3CN at -40 °C to 80 °C.

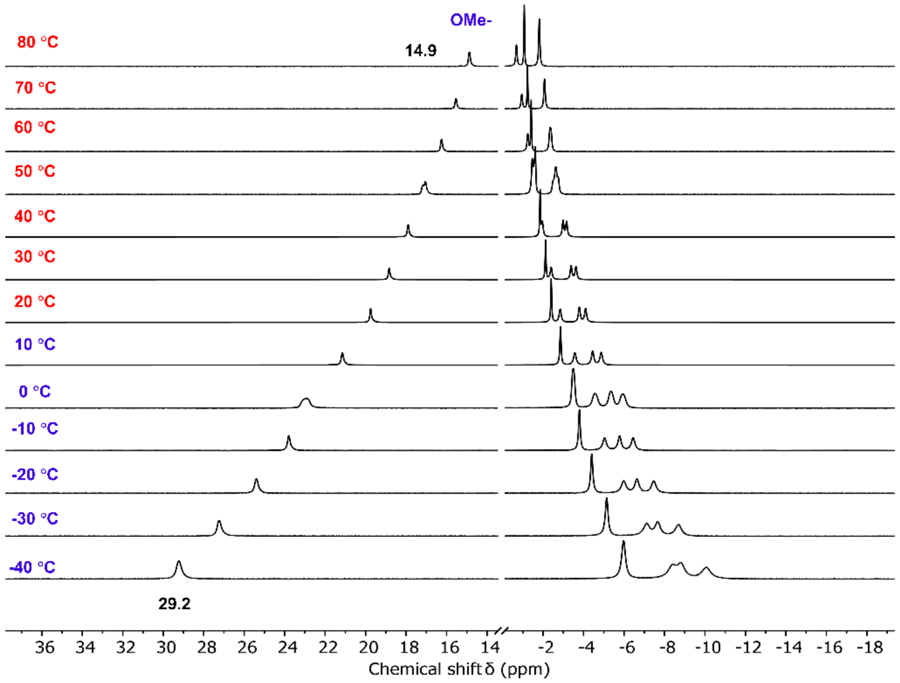

Figure S30. Variable Temperature [17]H NMR spectra (54.3 MHz) of $(TBA)_3[Er\{Mo_5O_{13}(OMe)_4NO\}_2]$ (**Er(Mo5)2**). Spectra obtained in CD_3CN at -40 °C to 80 °C.

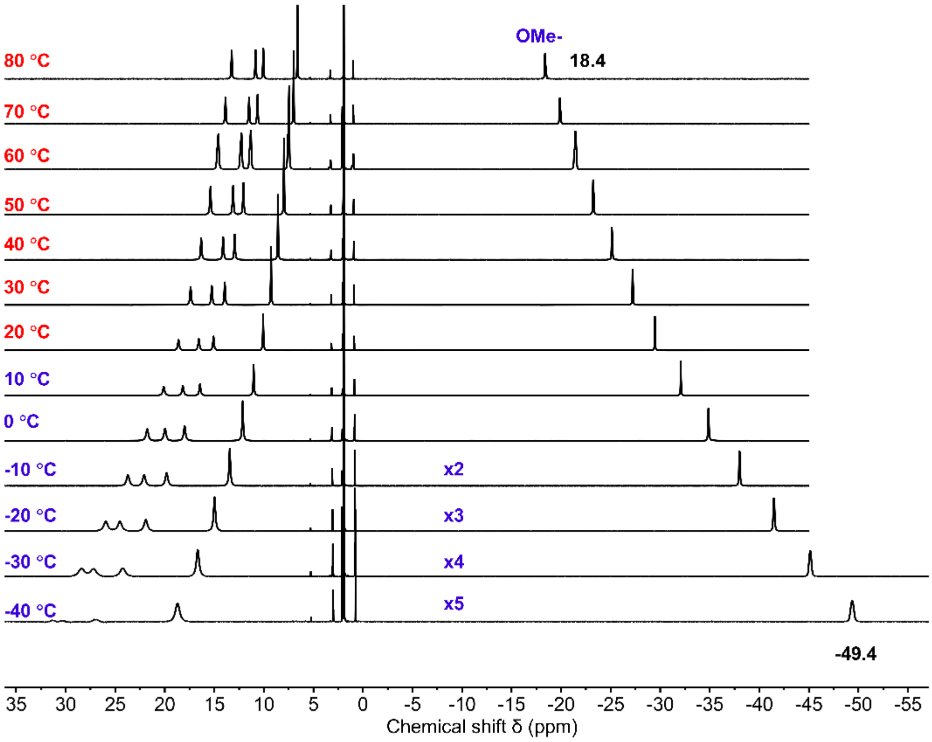

Figure S31. Variable Temperature ^{17}O NMR spectra (54.3 MHz) of $(TBA)_3[Tb\{Mo_5O_{13}(OMe)_4NO\}_2]$ (**$Tb(Mo_5)_2$**). Spectra obtained in CD_3CN at -40 °C to 80 °C.

S5. UV-Vis-NIR Spectra

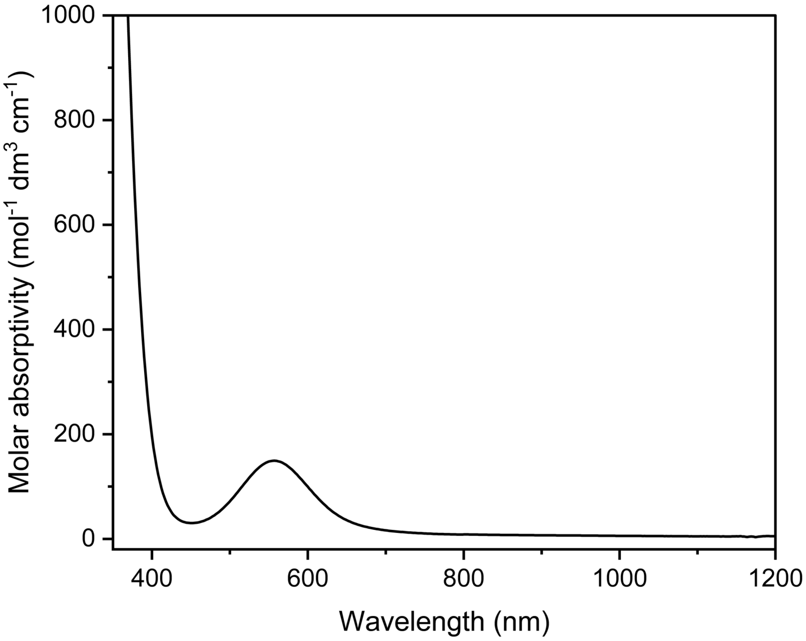

Figure S32. UV-Vis-NIR spectrum of a 1 mM solution $(TBA)_3[La\{Mo_5O_{13}(OMe)_4NO\}_2]$ (**La(Mo$_5$)$_2$**). The spectrum was acquired at room temperature in MeCN.

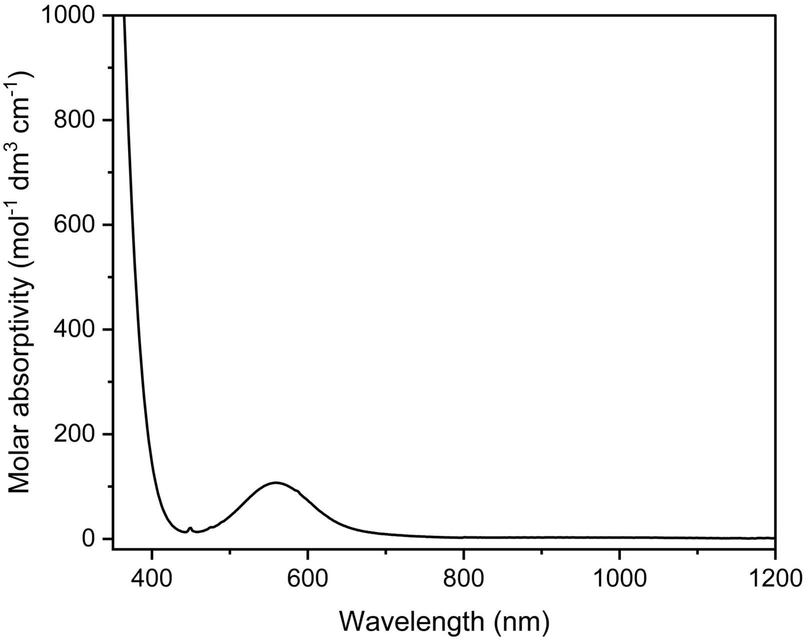

Figure S33. UV-Vis-NIR spectrum of a 1 mM solution $(TBA)_3[Pr\{Mo_5O_{13}(OMe)_4NO\}_2]$ (**Pr(Mo$_5$)$_2$**). The spectrum was acquired at room temperature in MeCN.

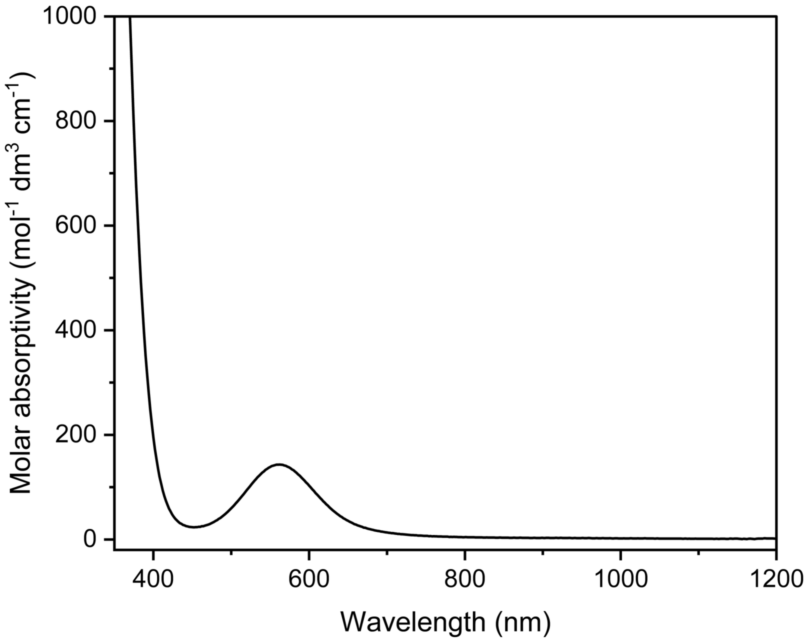

Figure S34. UV-Vis-NIR spectrum of a 1 mM solution $(TBA)_3[Nd\{Mo_5O_{13}(OMe)_4NO\}_2]$ (**Nd(Mo$_5$)$_2$**). The spectrum was acquired at room temperature in MeCN.

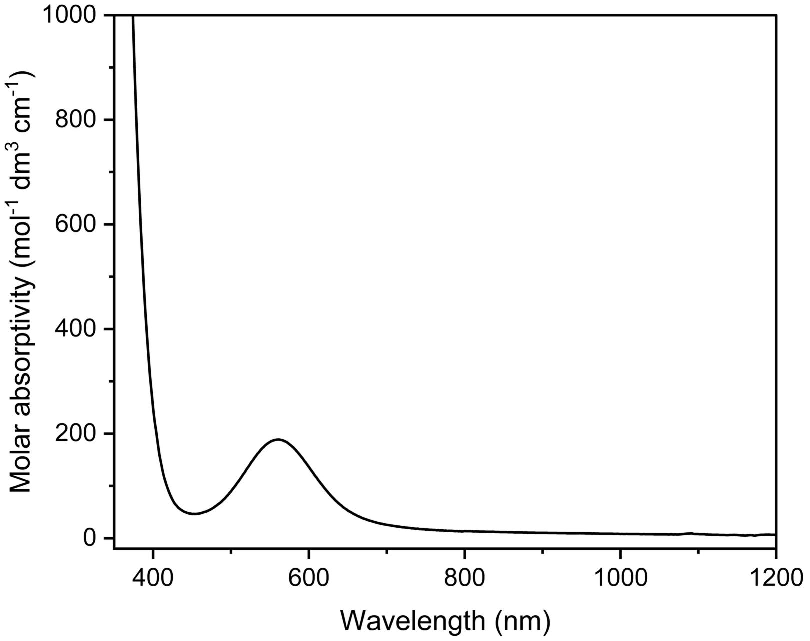

Figure S35. UV-Vis-NIR spectrum of a 1 mM solution $(TBA)_3[Sm\{Mo_5O_{13}(OMe)_4NO\}_2]$ (**Sm(Mo$_5$)$_2$**). The spectrum was acquired at room temperature in MeCN.

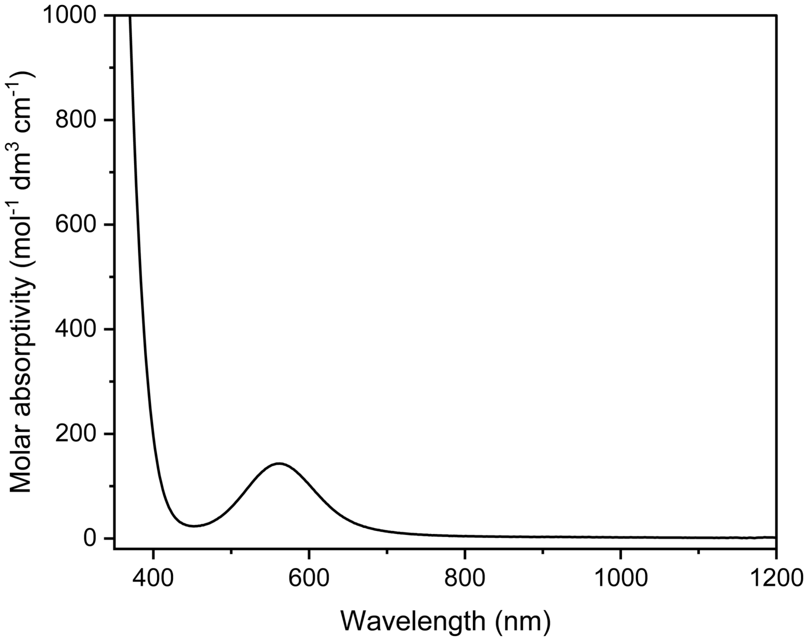

Figure S36. UV-Vis-NIR spectrum of a 1 mM solution $(TBA)_3[Eu\{Mo_5O_{13}(OMe)_4NO\}_2]$ (**Eu(Mo$_5$)$_2$**). The spectrum was acquired at room temperature in in MeCN.

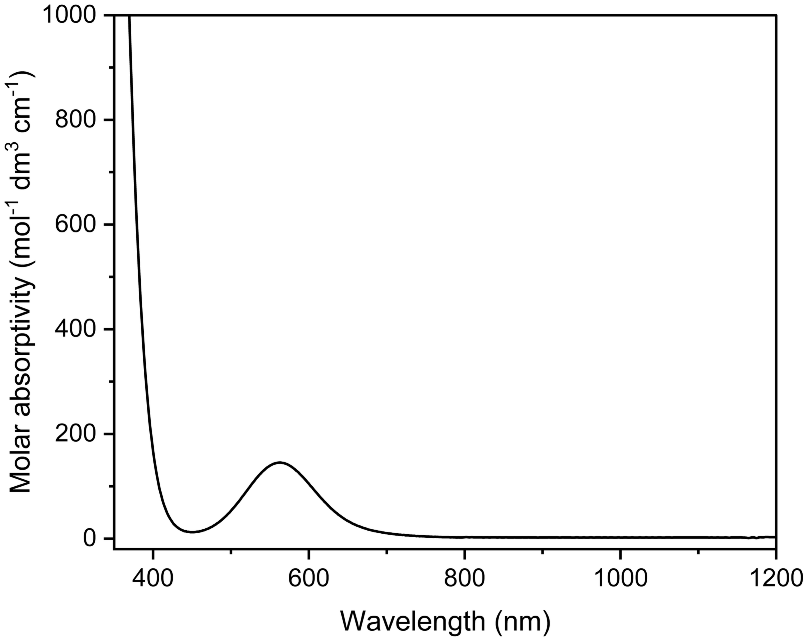

Figure S37. UV-Vis-NIR spectrum of a 1 mM solution $(TBA)_3[Gd\{Mo_5O_{13}(OMe)_4NO\}_2]$ (**Gd(Mo$_5$)$_2$**). The spectrum was acquired at room temperature in MeCN.

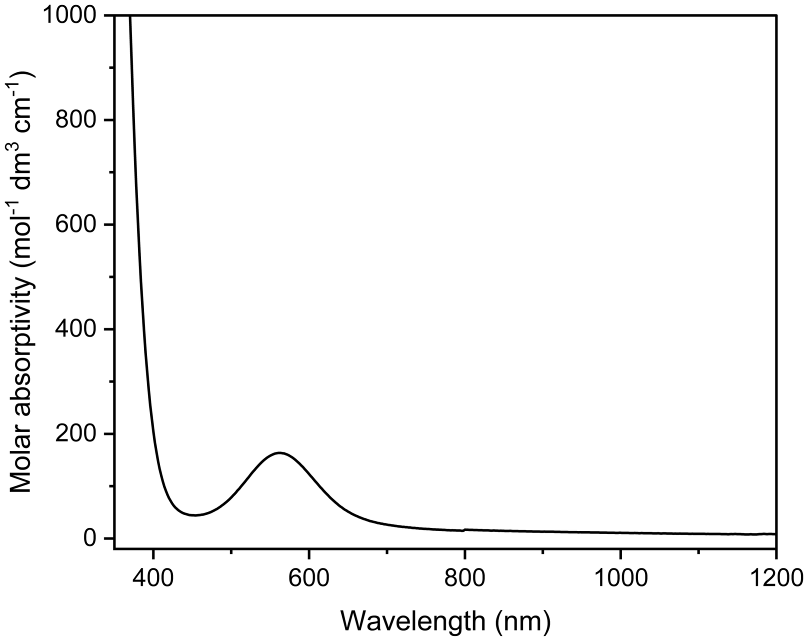

Figure S38. UV-Vis-NIR spectrum of a 1 mM solution $(TBA)_3[Tb\{Mo_5O_{13}(OMe)_4NO\}_2]$ (**Tb(Mo$_5$)$_2$**). The spectrum was acquired at room temperature in in MeCN.

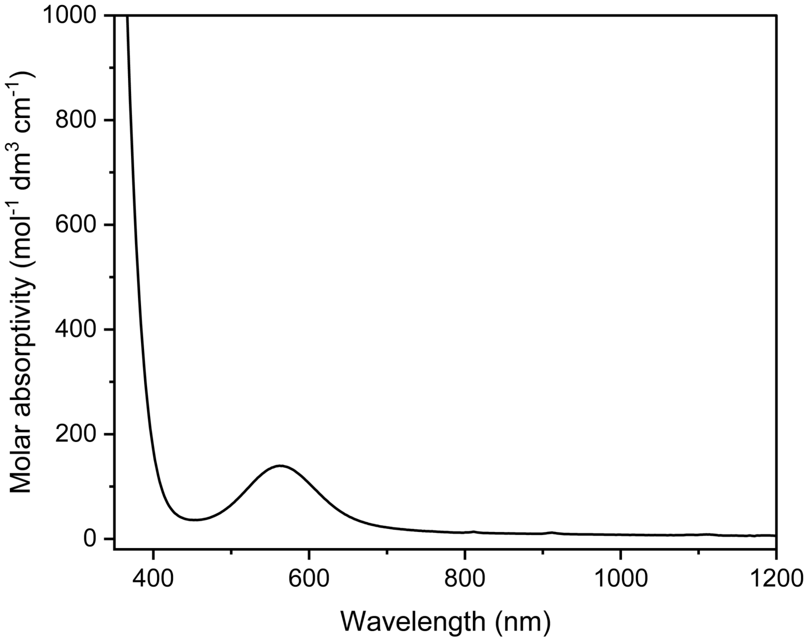

Figure S39. UV-Vis-NIR spectrum of a 1 mM solution $(TBA)_3[Dy\{Mo_5O_{13}(OMe)_4NO\}_2]$ (**Dy(Mo$_5$)$_2$**). The spectrum was acquired at room temperature in MeCN.

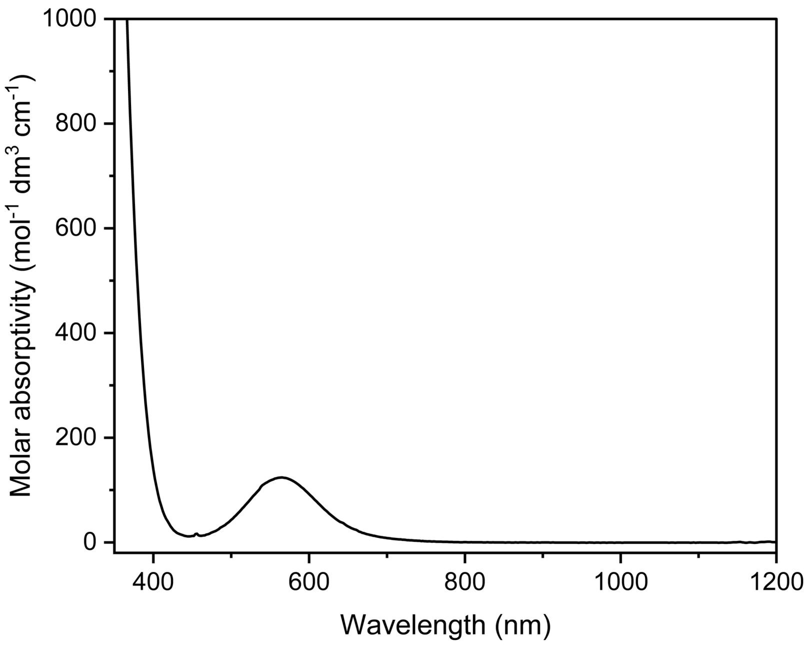

Figure S40. UV-Vis-NIR spectrum of a 1 mM solution $(TBA)_3[Ho\{Mo_5O_{13}(OMe)_4NO\}_2]$ (**Ho(Mo$_5$)$_2$**). The spectrum was acquired at room temperature in MeCN.

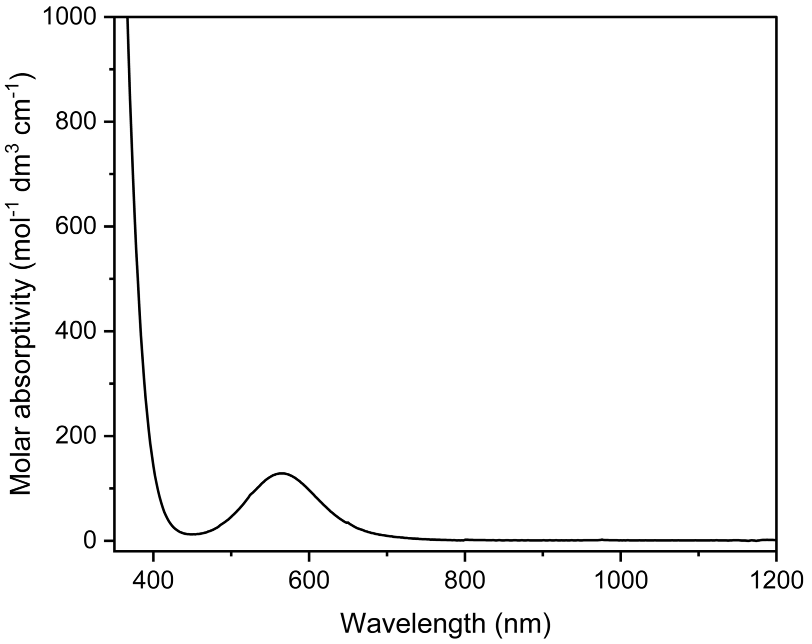

Figure S41. UV-Vis-NIR spectrum of a 1 mM solution $(TBA)_3[Er\{Mo_5O_{13}(OMe)_4NO\}_2]$ (**Er(Mo$_5$)$_2$**). The spectrum was acquired at room temperature in MeCN.

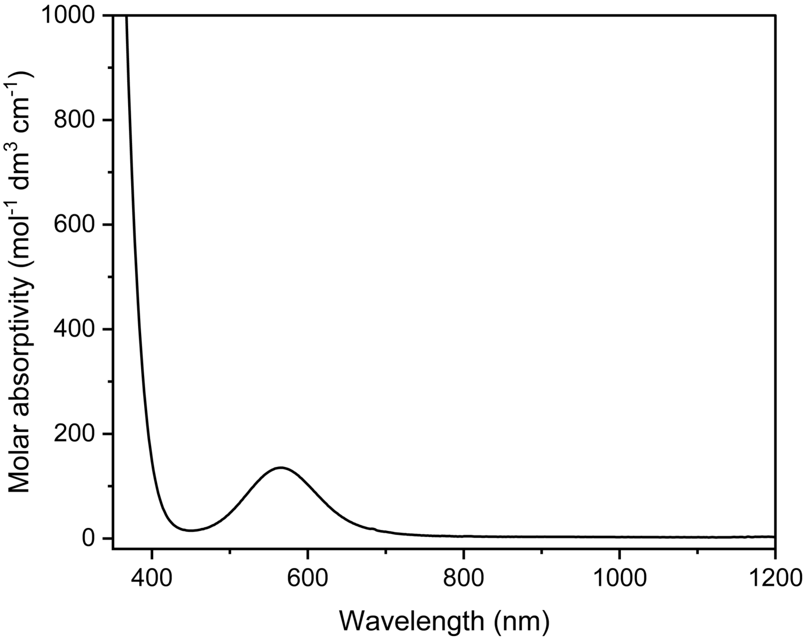

Figure S42. UV-Vis-NIR spectrum of a 1 mM solution $(TBA)_3[Tm\{Mo_5O_{13}(OMe)_4NO\}_2]$ (**Tm(Mo$_5$)$_2$**). The spectrum was acquired at room temperature in MeCN.

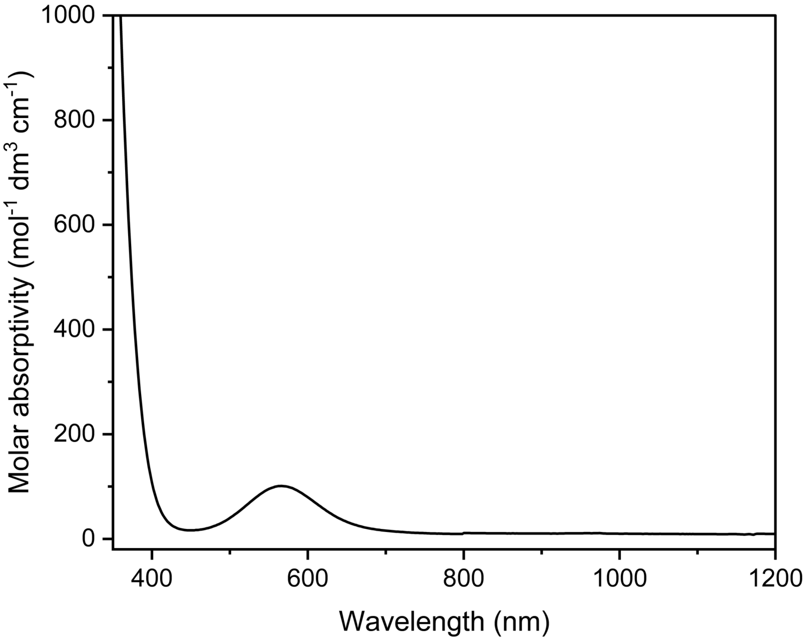

Figure S43. UV-Vis-NIR spectrum of a 1 mM solution $(TBA)_3[Yb\{Mo_5O_{13}(OMe)_4NO\}_2]$ (**Yb(Mo$_5$)$_2$**). The spectrum was acquired at room temperature in MeCN.

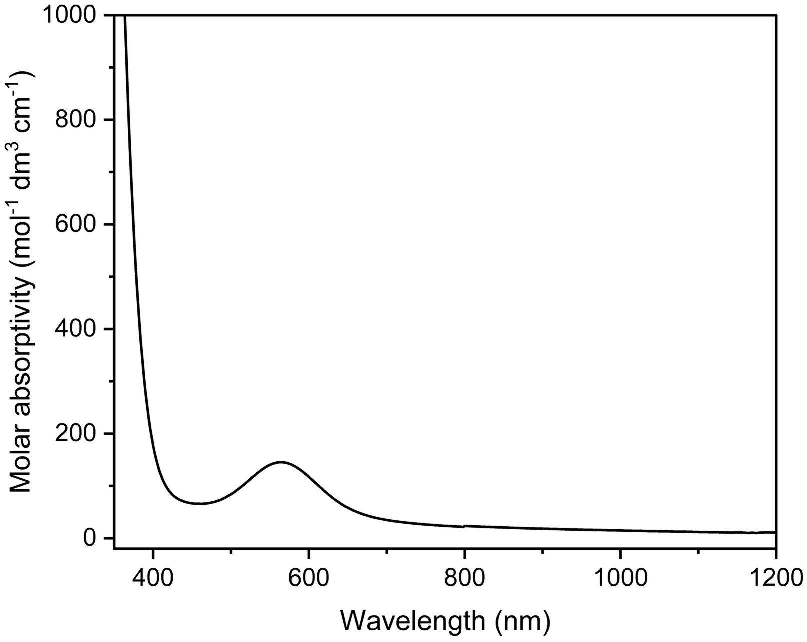

Figure S44. UV-Vis-NIR spectrum of a 1 mM solution $(TBA)_3[Lu\{Mo_5O_{13}(OMe)_4NO\}_2]$ (**Lu(Mo$_5$)$_2$**). The spectrum was acquired at room temperature in MeCN.

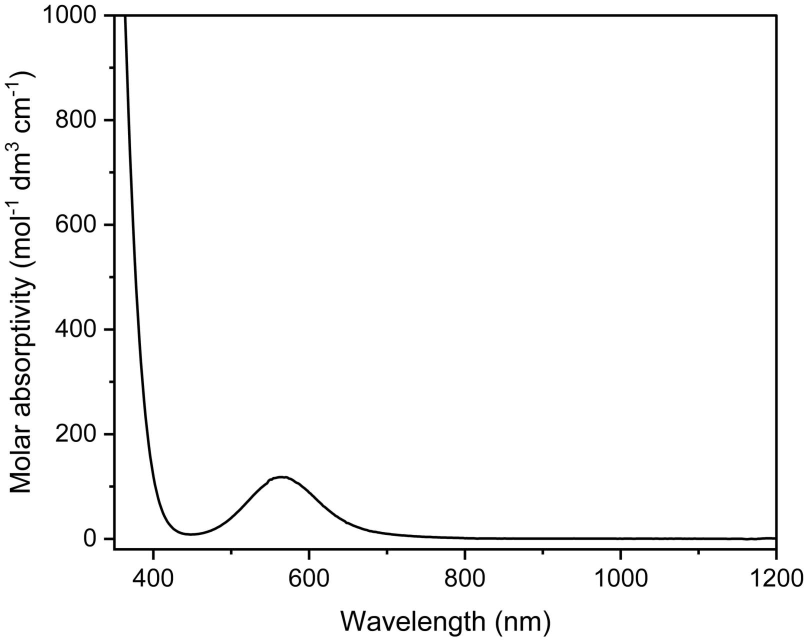

Figure S45. UV-Vis-NIR spectrum of a 1 mM solution $(TBA)_3[Y\{Mo_5O_{13}(OMe)_4NO\}_2]$ (**Y(Mo$_5$)$_2$**). The spectrum was acquired at room temperature in MeCN.

S6. Electrochemistry

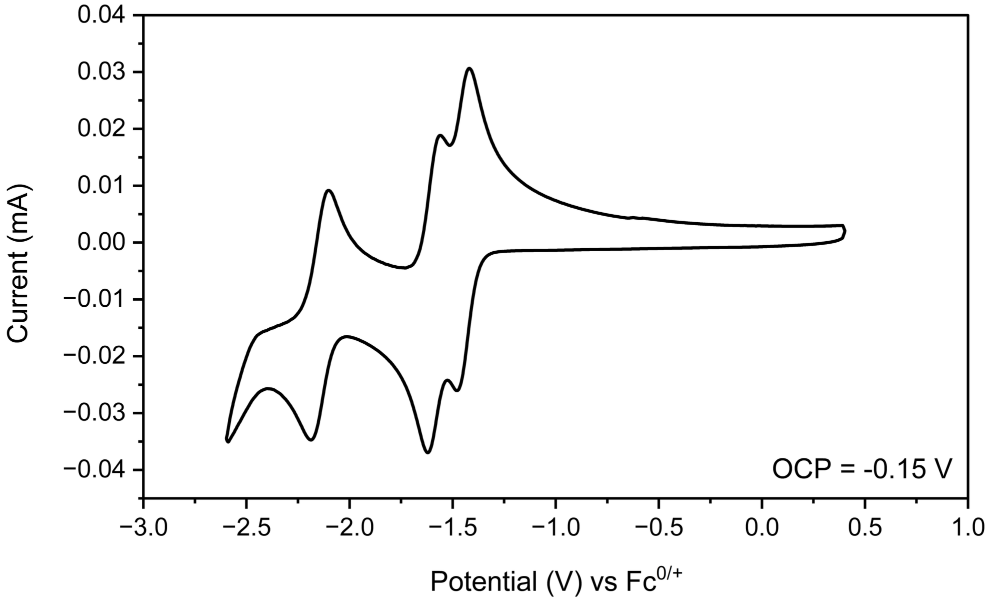

Figure S46. Cyclic voltammogram of a 1 mM solution $(TBA)_3[La\{Mo_5O_{13}(OMe)_4NO\}_2]$ (**La(Mo$_5$)$_2$**). The voltammogram was acquired in a 0.1 M solution of TBA(PF_6) in MeCN at room temperature.

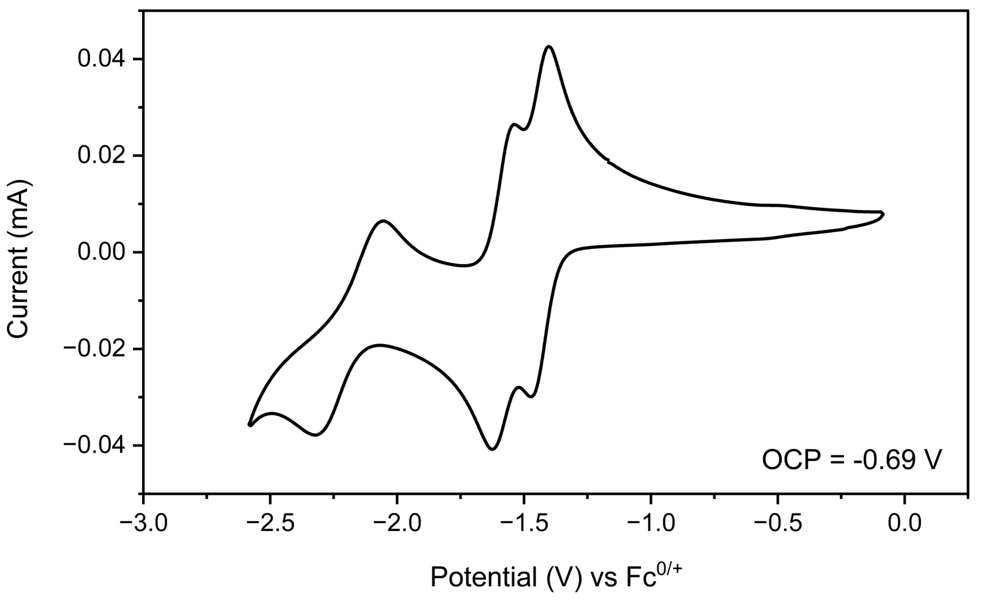

Figure S47. Cyclic voltammogram of a 1 mM solution $(TBA)_3[Pr\{Mo_5O_{13}(OMe)_4NO\}_2]$ (**Pr(Mo$_5$)$_2$**). The voltammogram was acquired in a 0.1 M solution of TBA(PF_6) in MeCN at room temperature.

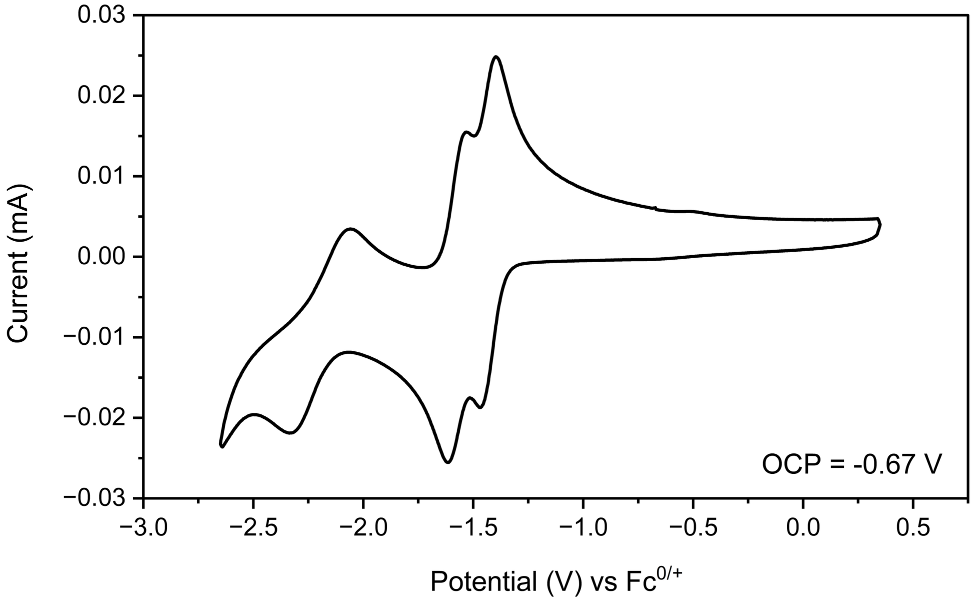

Figure S48. Cyclic voltammogram of a 1 mM solution $(TBA)_3[Nd\{Mo_5O_{13}(OMe)_4NO\}_2]$ (**Nd(Mo$_5$)$_2$**). The voltammogram was acquired in a 0.1 M solution of TBA(PF_6) in MeCN at room temperature.

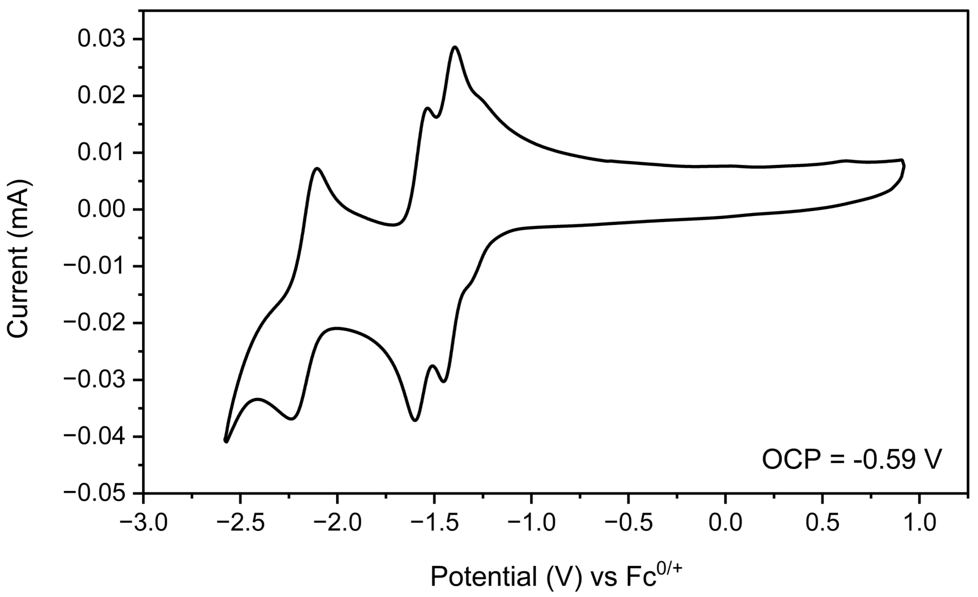

Figure S49. Cyclic voltammogram of a 1 mM solution $(TBA)_3[Sm\{Mo_5O_{13}(OMe)_4NO\}_2]$ (**Sm(Mo$_5$)$_2$**). The voltammogram was acquired in a 0.1 M solution of TBA(PF_6) in MeCN at room temperature. The shoulder feature observed at *ca.* −1.25 V is attributed to an unidentified impurity.

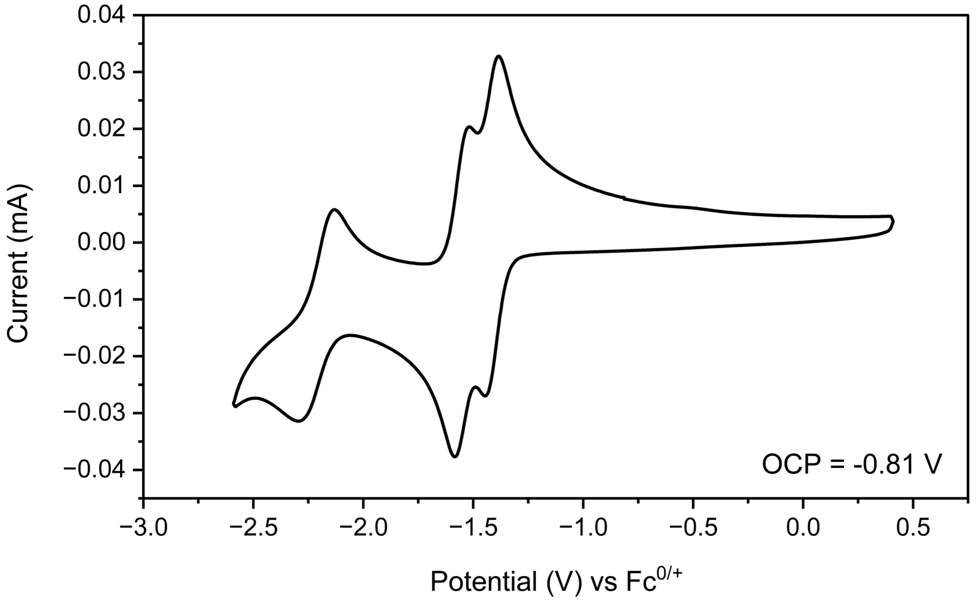

Figure S50. Cyclic voltammogram of a 1 mM solution $(TBA)_3[Eu\{Mo_5O_{13}(OMe)_4NO\}_2]$ (**$Eu(Mo_5)_2$**). The voltammogram was acquired in a 0.1 M solution of $TBA(PF_6)$ in MeCN at room temperature.

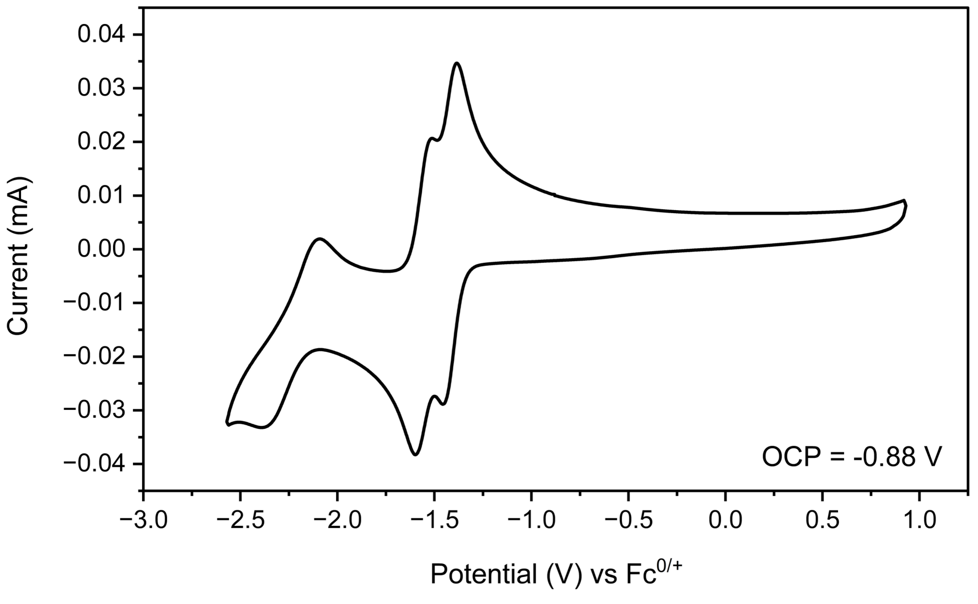

Figure S51. Cyclic voltammogram of a 1 mM solution $(TBA)_3[Gd\{Mo_5O_{13}(OMe)_4NO\}_2]$ (**$Gd(Mo_5)_2$**). The voltammogram was acquired in a 0.1 M solution of $TBA(PF_6)$ in MeCN at room temperature.

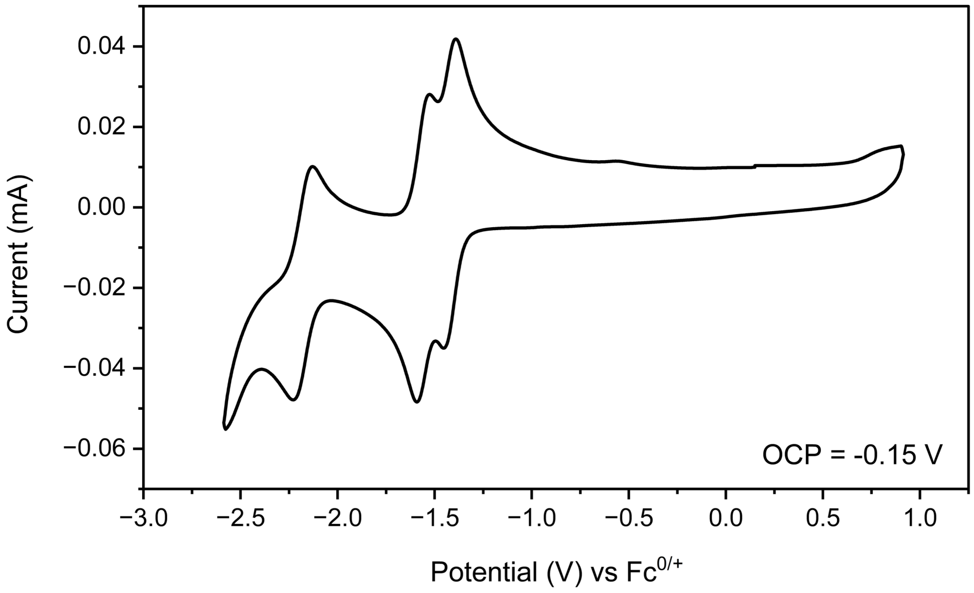

Figure S52. Cyclic voltammogram of a 1 mM solution $(TBA)_3[Tb\{Mo_5O_{13}(OMe)_4NO\}_2]$ (**Tb(Mo$_5$)$_2$**). The voltammogram was acquired in a 0.1 M solution of $TBA(PF_6)$ in MeCN at room temperature.

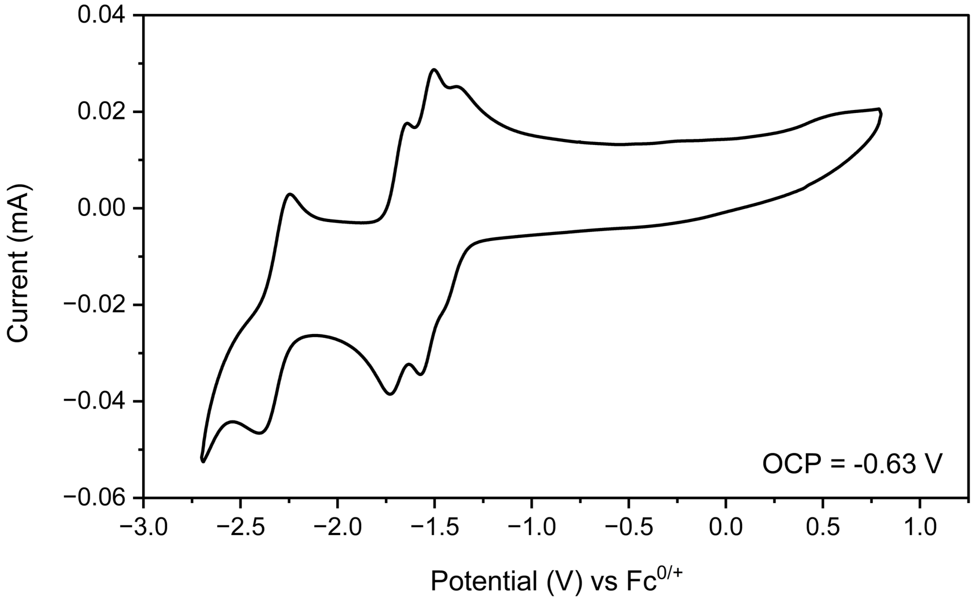

Figure S53. Cyclic voltammogram of a 1 mM solution $(TBA)_3[Dy\{Mo_5O_{13}(OMe)_4NO\}_2]$ (**Dy(Mo$_5$)$_2$**). The voltammogram was acquired in a 0.1 M solution of $TBA(PF_6)$ in MeCN at room temperature. The feature observed at ca. -1.37 V (reduction curve) and -1.45 V (oxidation curve) is attributed to an unidentified impurity.

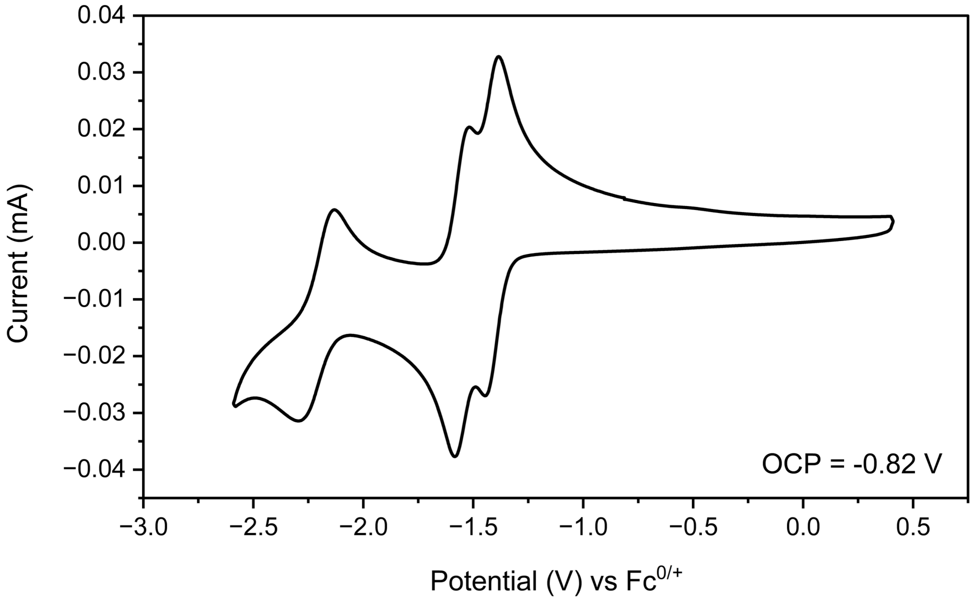

Figure S54. Cyclic voltammogram of a 1 mM solution $(TBA)_3[Ho\{Mo_5O_{13}(OMe)_4NO\}_2]$ (**Ho(Mo$_5$)$_2$**). The voltammogram was acquired in a 0.1 M solution of $TBA(PF_6)$ in MeCN at room temperature.

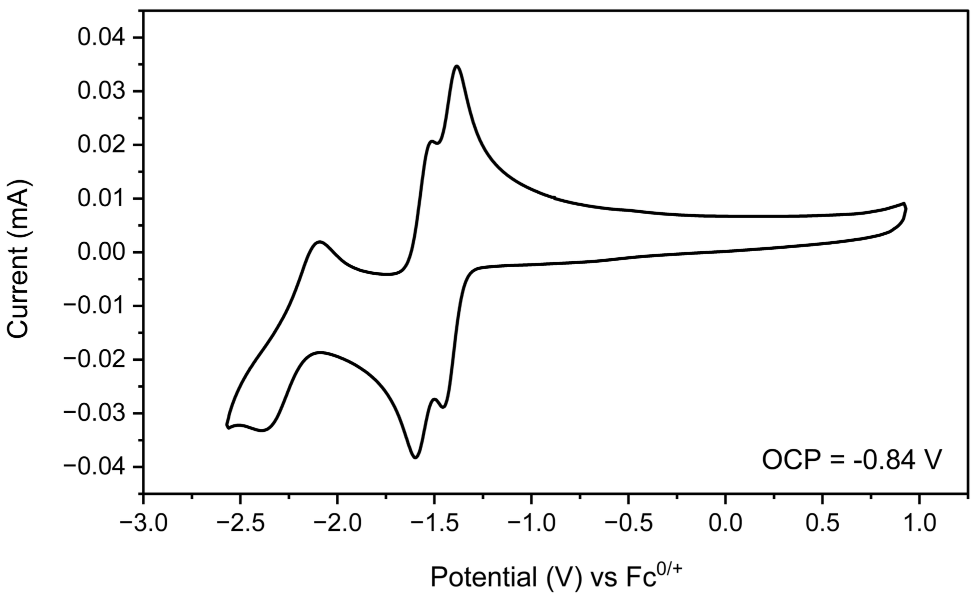

Figure S55. Cyclic voltammogram of a 1 mM solution $(TBA)_3[Er\{Mo_5O_{13}(OMe)_4NO\}_2]$ (**Er(Mo$_5$)$_2$**). The voltammogram was acquired in a 0.1 M solution of $TBA(PF_6)$ in MeCN at room temperature.

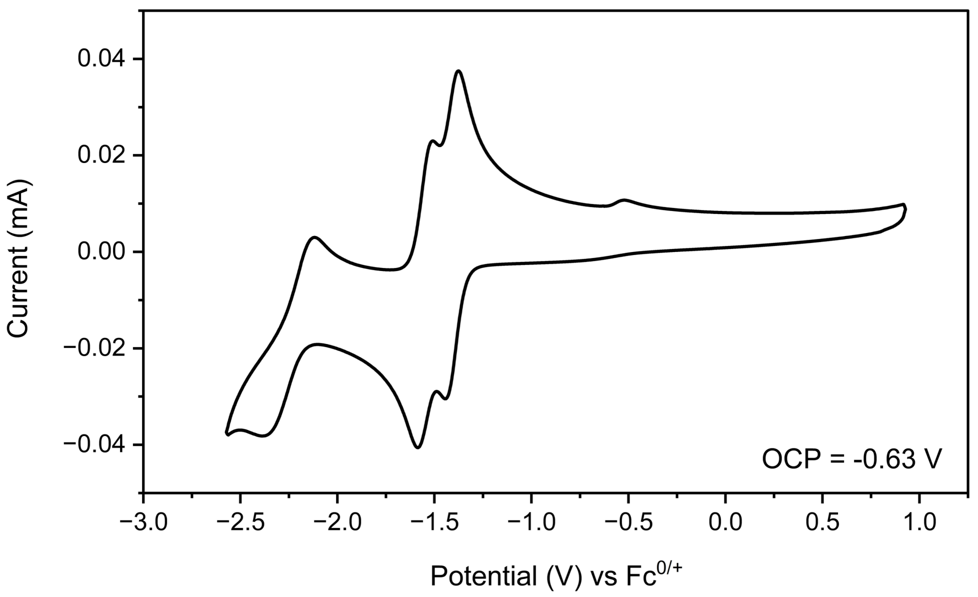

Figure S56. Cyclic voltammogram of a 1 mM solution $(TBA)_3[Tm\{Mo_5O_{13}(OMe)_4NO\}_2]$ (**Tm(Mo$_5$)$_2$**). The voltammogram was acquired in a 0.1 M solution of TBA(PF_6) in MeCN at room temperature.

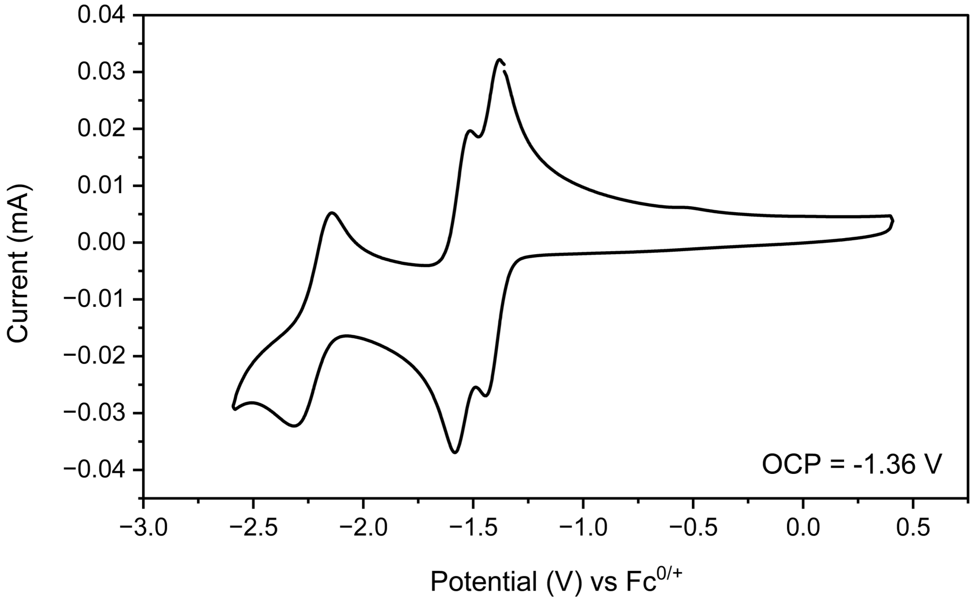

Figure S57. Cyclic voltammogram of a 1 mM solution $(TBA)_3[Yb\{Mo_5O_{13}(OMe)_4NO\}_2]$ (**Yb(Mo$_5$)$_2$**). The voltammogram was acquired in a 0.1 M solution of TBA(PF_6) in MeCN at room temperature.

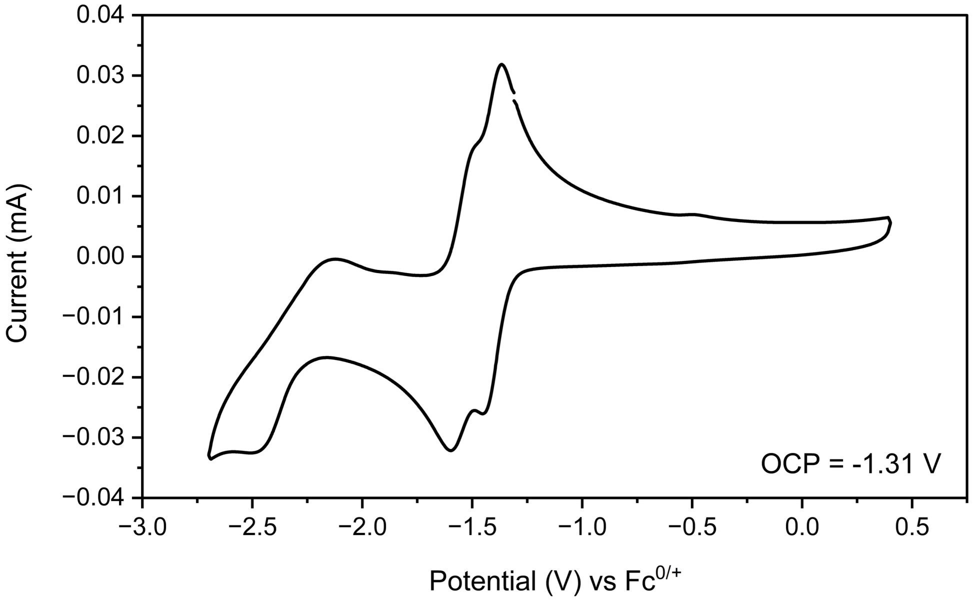

Figure S58. Cyclic voltammogram of a 1 mM solution $(TBA)_3[Lu\{Mo_5O_{13}(OMe)_4NO\}_2]$ (**Lu(Mo$_5$)$_2$**). The voltammogram was acquired in a 0.1 M solution of $TBA(PF_6)$ in MeCN at room temperature.

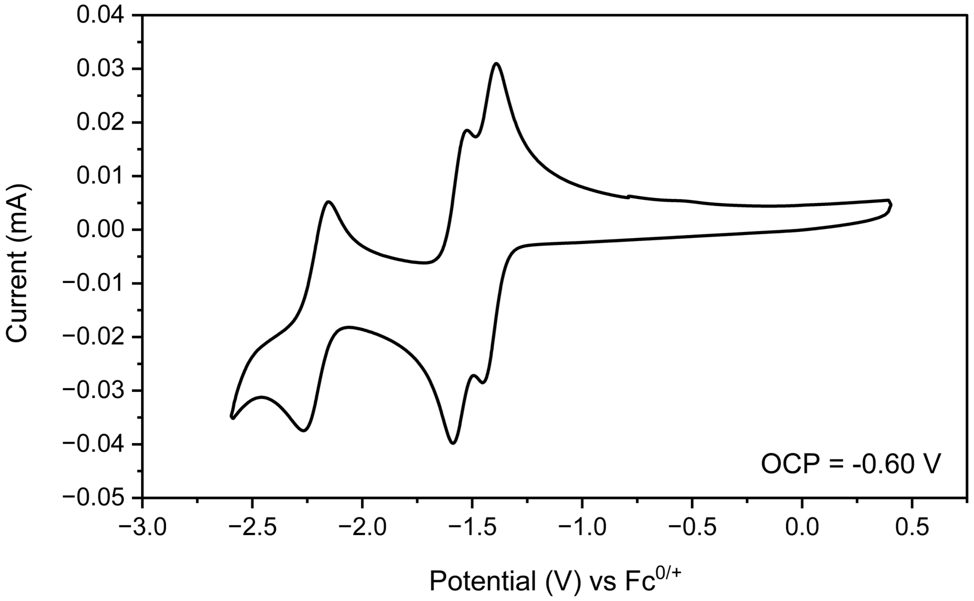

Figure S59. Cyclic voltammogram of a 1 mM solution $(TBA)_3[Y\{Mo_5O_{13}(OMe)_4NO\}_2]$ (**Y(Mo$_5$)$_2$**). The voltammogram was acquired in a 0.1 M solution of $TBA(PF_6)$ in MeCN at room temperature.

S7. Single Crystal X-ray Diffraction

Additional refinement details

For **Sr(Mo$_5$)$_2$**, the asymmetric unit contains one tetraanionic SrMo$_{10}$ cluster and six tetrabutylammonium cations in general positions and one-half of a tetraanionic SrMo$_{10}$ cluster on a crystallographic inversion center that coincides with Sr atom. The Sr1…O35, Sr1…O36, and Sr…O54 distances are 3.733(3), 3.729(3), and 3.709(3) Å, respectively.

For **Y(Mo$_5$)$_2$**, the asymmetric unit contains one trianionic YMo$_{10}$ cluster, three tetrabutylammonium cations, and one diethyl ether solvent molecule, all in general positions. The Y1…O35 and Yu1…O36 distances are both 3.456(2) Å.

For **Tm(Mo$_5$)$_2$**, the asymmetric unit contains one trianionic TmMo$_{10}$ cluster, three tetrabutylammonium cations, and one diethyl ether solvent molecule, all in general positions. The Tm1…O35 and Tm1…O36 distances are 3.433(2) and 3.435(2) Å, respectively.

For **Yb(Mo$_5$)$_2$**, The asymmetric unit contains one trianionic YbMo$_{10}$ cluster, three tetrabutylammonium cations, and one diethyl ether solvent molecule, all in general positions. The Yb1…O35 and Yb1…O36 distances are 3.436(2) and 3.434(2) Å, respectively.

For **Lu(Mo$_5$)$_2$**, The asymmetric unit contains one trianionic LuMo$_{10}$ cluster, three tetrabutylammonium cations, and one diethyl ether solvent molecule, all in general positions. The Lu1…O35 and Lu1…O36 distances are 3.428(2) and 3.429(2) Å, respectively.

Table S2. Crystal data and structure refinement for **Tm(Mo$_5$)$_2$.**

Empirical formula	C60 H142 Mo10 N5 O37 Tm	
Formula weight	2654.11	
Temperature	100.00(10) K	
Wavelength	1.54184 Å	
Crystal system	monoclinic	
Space group	$P2_1/c$	
Unit cell dimensions	*a* = 23.75161(9) Å	a = 90°
	b = 22.43856(9) Å	b = 107.2477(4)°
	c = 18.36130(7) Å	g = 90°
Volume	9345.64(6) Å^3	
Z	4	
Density (calculated)	1.886 Mg/m^3	
Absorption coefficient	12.964 mm^{-1}	
F(000)	5288	
Crystal color, morphology	blue-violet, block	
Crystal size	0.225 x 0.209 x 0.081 mm^3	
Theta range for data collection	2.770 to 80.482°	
Index ranges	$-30 \le h \le 30$, $-28 \le k \le 28$, $-23 \le l \le 20$	
Reflections collected	161332	
Independent reflections	20171 [*R*(int) = 0.0568]	
Observed reflections	19877	
Completeness to theta = 74.504°	99.9%	
Absorption correction	Multi-scan	
Max. and min. transmission	1.00000 and 0.33790	
Refinement method	Full-matrix least-squares on F^2	
Data / restraints / parameters	20171 / 0 / 1040	
Goodness-of-fit on F^2	1.150	
Final *R* indices [*I*>2sigma(*I*)]	*R*1 = 0.0293, *wR*2 = 0.0693	
R indices (all data)	*R*1 = 0.0299, *wR*2 = 0.0697	
Largest diff. peak and hole	1.318 and -0.925 e.Å^{-3}	

Table S3. Crystal data and structure refinement for **Yb(Mo$_5$)$_2$.**

Empirical formula	C60 H142 Mo10 N5 O37 Yb	
Formula weight	2658.22	
Temperature	100.00(10) K	
Wavelength	1.54184 Å	
Crystal system	monoclinic	
Space group	P 1 21/c 1	
Unit cell dimensions	*a* = 23.72926(8) Å	a = 90°
	b = 22.44883(7) Å	b = 107.2886(3)°
	c = 18.35927(6) Å	g = 90°
Volume	9338.02(5) Å^3	
Z	4	
Density (calculated)	1.891 Mg/m^3	
Absorption coefficient	13.048 mm^{-1}	
F(000)	5292	
Crystal color, morphology	blue-violet, needle	
Crystal size	0.178 x 0.147 x 0.048 mm^3	
Theta range for data collection	2.771 to 80.573°	
Index ranges	$-30 \leq h \leq 29$, $-28 \leq k \leq 28$, $-19 \leq l \leq 23$	
Reflections collected	161015	
Independent reflections	20183 [*R*(int) = 0.0439]	
Observed reflections	19575	
Completeness to theta = 74.504°	100.0%	
Absorption correction	Multi-scan	
Max. and min. transmission	1.00000 and 0.38502	
Refinement method	Full-matrix least-squares on F^2	
Data / restraints / parameters	20183 / 0 / 1040	
Goodness-of-fit on F^2	1.093	
Final *R* indices [*I*>2sigma(*I*)]	*R*1 = 0.0298, *wR*2 = 0.0752	
R indices (all data)	*R*1 = 0.0310, *wR*2 = 0.0759	
Largest diff. peak and hole	1.427 and -1.065 e.Å^{-3}	

Table S4. Crystal data and structure refinement for **Lu(Mo$_5$)$_2$.**

Empirical formula	C60 H142 Lu Mo10 N5 O37	
Formula weight	2660.15	
Temperature	100.00(10) K	
Wavelength	1.54184 Å	
Crystal system	monoclinic	
Space group	$P2_1/c$	
Unit cell dimensions	*a* = 23.73410(10) Å	a = 90°
	b = 22.41850(10) Å	b = 107.2180(10)°
	c = 18.37180(10) Å	g = 90°
Volume	9337.24(9) Å^3	
Z	4	
Density (calculated)	1.892 Mg/m^3	
Absorption coefficient	13.220 mm^{-1}	
F(000)	5296	
Crystal color, morphology	violet, block	
Crystal size	0.161 x 0.132 x 0.069 mm^3	
Theta range for data collection	2.772 to 80.415°	
Index ranges	$-30 \le h \le 30$, $-28 \le k \le 25$, $-22 \le l \le 23$	
Reflections collected	161310	
Independent reflections	20149 [*R*(int) = 0.0438]	
Observed reflections	19393	
Completeness to theta = 74.504°	100.0%	
Absorption correction	Multi-scan	
Max. and min. transmission	1.00000 and 0.38333	
Refinement method	Full-matrix least-squares on F^2	
Data / restraints / parameters	20149 / 0 / 1040	
Goodness-of-fit on F^2	1.090	
Final *R* indices [*I*>2sigma(*I*)]	*R*1 = 0.0272, *wR*2 = 0.0683	
R indices (all data)	*R*1 = 0.0287, *wR*2 = 0.0692	
Largest diff. peak and hole	1.192 and -0.967 e.Å^{-3}	

Table S5. Crystal data and structure refinement for **$Y(Mo_5)_2$.**

Empirical formula	C60 H142 Mo10 N5 O37 Y	
Formula weight	2574.09	
Temperature	100.00(10) K	
Wavelength	1.54184 Å	
Crystal system	monoclinic	
Space group	$P2_1/c$	
Unit cell dimensions	*a* = 23.77385(9) Å	a = 90°
	b = 22.47343(8) Å	b = 107.4037(4)°
	c = 18.33998(7) Å	g = 90°
Volume	9350.11(6) $Å^3$	
Z	4	
Density (calculated)	1.829 Mg/m^3	
Absorption coefficient	12.060 mm^{-1}	
F(000)	5168	
Crystal color, morphology	blue-violet, block	
Crystal size	0.215 x 0.197 x 0.105 mm^3	
Theta range for data collection	2.768 to 80.475°	
Index ranges	$-30 \le h \le 27$, $-26 \le k \le 28$, $-23 \le l \le 23$	
Reflections collected	161011	
Independent reflections	20183 [*R*(int) = 0.0472]	
Observed reflections	19782	
Completeness to theta = 74.504°	100.0%	
Absorption correction	Multi-scan	
Max. and min. transmission	1.00000 and 0.49804	
Refinement method	Full-matrix least-squares on F^2	
Data / restraints / parameters	20183 / 21 / 1059	
Goodness-of-fit on F^2	1.108	
Final *R* indices [I>2sigma(I)]	$R1$ = 0.0292, $wR2$ = 0.0700	
R indices (all data)	$R1$ = 0.0300, $wR2$ = 0.0705	
Largest diff. peak and hole	1.363 and -1.058 e.$Å^{-3}$	

Table S6. Crystal data and structure refinement for **Sr(Mo$_5$)$_2$.**

Empirical formula	C72 H168 Mo10 N6 O36 Sr	
Formula weight	2741.13	
Temperature	100.00(10) K	
Wavelength	1.54184 Å	
Crystal system	monoclinic	
Space group	$P2_1/n$	
Unit cell dimensions	*a* = 19.22552(7) Å	a = 90°
	b = 17.83839(8) Å	b = 90.8299(3)°
	c = 46.18943(18) Å	g = 90°
Volume	15839.12(11) Å^3	
Z	6	
Density (calculated)	1.724 Mg/m^3	
Absorption coefficient	10.647 mm^{-1}	
F(000)	8328	
Crystal color, morphology	blue-violet, block	
Crystal size	0.162 x 0.121 x 0.065 mm^3	
Theta range for data collection	2.477 to 80.245°	
Index ranges	$-24 \le h \le 23$, $-22 \le k \le 17$, $-58 \le l \le 58$	
Reflections collected	268630	
Independent reflections	34053 [*R*(int) = 0.0430]	
Observed reflections	31649	
Completeness to theta = 74.504°	99.7%	
Absorption correction	Multi-scan	
Max. and min. transmission	1.00000 and 0.63910	
Refinement method	Full-matrix least-squares on F^2	
Data / restraints / parameters	34053 / 0 / 1726	
Goodness-of-fit on F^2	1.160	
Final *R* indices [*I*>2sigma(*I*)]	*R*1 = 0.0432, *wR*2 = 0.0991	
R indices (all data)	*R*1 = 0.0472, *wR*2 = 0.1006	
Largest diff. peak and hole	2.598 and -1.534 e.Å^{-3}	

Table S7. Average bond length data for the structures discussed. All values in Å. A schematic is given below to highlight bond assignments.

	$Sr(Mo_5)_2$	$Y(Mo_5)_2$	$Tm(Mo_5)_2$	$Yb(Mo_5)_2$	$Lu(Mo_5)_2$
**Mo-O-Mo**	1.921	1.918	1.917	1.918	1.919
**Mo=O**	1.708	1.700	1.701	1.701	1.701
**Mo-O-Ln**	1.732	1.753	1.753	1.753	1.752
**Mo-(μ_5)O (ax)**	2.335	2.120	2.118	2.115	2.116
**Mo-O (eq)**	2.007	2.009	2.009	2.010	2.010
**Mo-O (ax)**	2.300	2.244	2.244	2.245	2.245
**Mo-NO**	1.771	1.771	1.771	1.771	1.771
**O-O**	3.158	3.036	2.990	2.984	2.970
**μ_5O- μ_5O**	7.467	6.911	6.868	6.869	6.857
**Ln-O**	2.607	2.365	2.343	2.335	2.328

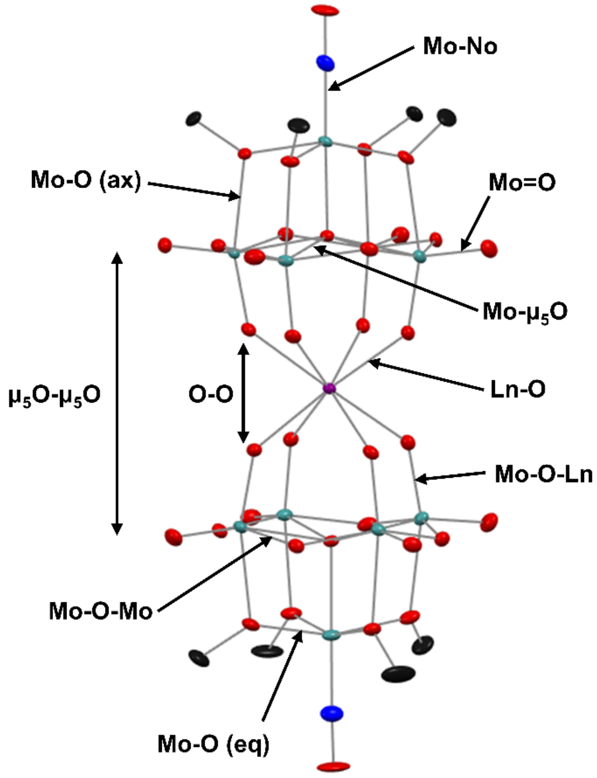

Table S8. Structural parameters in **Ln(Mo$_5$)$_2$** complexes.

Ln	Ionic radius (Å)	<O-Ln-O (º)	d(O-O) (Å)	Avg. Ln-O (Å)	Skew angle (º)	|PD$_1$-PD$_2$| (Å)
**La^{3+}**	1.16	176.959	7.158	2.533	4.84	0.027
**Ce^{3+}**	1.143	176.762	7.095	2.483	8.30	0.028
**Pr^{3+}**	1.126	177.716	7.109	2.473	6.20	0.055
**Nd^{3+}**	1.109	177.31	7.055	2.459	4.55	0.03
**Sm^{3+}**	1.079	177.408	7.006	2.428	4.14	0.032
**Eu^{3+}**	1.066	177.61	6.959	2.413	6.51	0.013
**Gd^{3+}**	1.053	177.48	6.956	2.403	6.43	0.012
**Tb^{3+}**	1.04	178.333	6.942	2.394	6.48	0.004
**Dy^{3+}**	1.027	177.842	6.938	2.386	5.84	0.013
**Ho^{3+}**	1.015	177.928	6.906	2.371	5.63	0.01
**Er^{3+}**	1.004	177.946	6.891	2.359	5.49	0.015
**Tm^{3+}**	0.994	178.876	6.868	2.343	5.85	0.006
**Yb^{3+}**	0.985	178.883	6.869	2.335	5.88	0.005
**Lu^{3+}**	0.977	179.111	6.857	2.328	5.67	0.005

Table S9. Structural parameters in (TBA)$_3$[Ln{Mo$_5$O$_{13}$(OMe)$_4$NNC$_6$H$_4$NO$_2$}$_2$] complexes.

Ln	Ionic radius (Å)	<O-Ln-O (º)	d(O-O) (Å)	Avg. Ln-O (Å)	Skew angle (º)	|PD$_1$-PD$_2$| (Å)	Plane angle	CShM (SAPR)
**Tb^{3+}**	1.04	179.16	6.953	2.395	0.91	0.009	1.4	0.29326
**Dy^{3+}**	1.027	179.18	6.936	2.378	0.84	0.015	1.32	0.28703
**Ho^{3+}**	1.015	176.62	6.924	2.372	5.86	0.012	3.79	0.52007
**Er^{3+}**	1.004	177.62	6.913	2.363	5.01	0.006	2.67	0.40806
**Yb^{3+}**	0.985	176.9	6.878	2.34	5.70	0.013	3.41	0.45155

Table S10. Results for 8-coorditate square antiprismatic (SAPR-8) and cubic (CU-8) continuous shape measurement (CShM) calculations using program SHAPE. Positions in Cartesian coordinates (x,y,z).

	Structure	**La(Mo5)2**	
La	1.4448	7.0378	11.1556
O	0.8751	5.1670	12.7661
O	2.9269	7.2064	13.1872
O	1.8571	4.8099	10.0466
O	1.8036	9.5263	11.0834
O	1.3031	7.8413	8.7826
O	3.8640	6.8307	10.4585
O	-0.8025	6.4947	10.2291
O	-0.3194	8.1601	12.5558

SAPR-8		Ideal structure	CShM =	0.79050
La	M	1.4392	7.0083	11.1405
O	L1	0.7021	5.1894	12.6999
O	L2	2.7436	7.3840	13.2478
O	L4	1.7985	4.8749	9.8746
O	L7	2.0509	9.4388	11.1073
O	L8	1.3826	7.6646	8.7221
O	L3	3.8399	7.0695	10.4224
O	L5	-0.8362	6.3353	10.3326
O	L6	-0.1679	8.1095	12.7178

	Structure	**Pr(Mo5)2**	
Pr	1.3280	7.0245	11.2076
O	-0.8685	6.4848	10.2686
O	1.2550	7.8186	8.8921
O	1.7202	9.4576	11.1923
O	-0.4177	8.1042	12.5869
O	2.7903	7.2227	13.1995
O	0.7830	5.2006	12.8035
O	1.7354	4.8139	10.1290
O	3.7139	6.8289	10.5209

SAPR-8		Ideal structure	CShM =	0.75843
Pr	M	1.3377	6.9951	11.2000
O	L1	-0.8927	6.3354	10.3892
O	L2	1.2993	7.6430	8.8239
O	L3	1.9397	9.3835	11.1746
O	L4	-0.2523	8.0759	12.7399
O	L8	2.6047	7.3615	13.2804
O	L5	0.6019	5.2062	12.7251
O	L6	1.6991	4.9001	9.9560
O	L7	3.7019	7.0554	10.5113

	Structure	$Nd(Mo_5)_2$	
Nd	1.3656	7.0210	11.1252
O	0.7822	5.2002	12.7055
O	2.7909	7.2150	13.1146
O	1.7629	4.8503	10.0253
O	1.7460	9.4495	11.0649
O	1.2557	7.7859	8.8010
O	3.7393	6.8484	10.4425
O	-0.8247	6.4700	10.2269
O	-0.3431	8.1072	12.5180

SAPR-8	Ideal structure		CShM =	0.60585
Nd	M	1.3639	6.9942	11.1138
O	L1	0.6398	5.2164	12.6386
O	L2	2.6343	7.3639	13.1776
O	L4	1.7191	4.9067	9.8783
O	L7	1.9614	9.3716	11.0807
O	L8	1.3143	7.6341	8.7477
O	L3	3.7136	7.0542	10.4172
O	L5	-0.8593	6.3345	10.3185
O	L6	-0.2121	8.0720	12.6515

	Structure	$Sm(Mo_5)_2$	
Sm	5.7251	7.0244	0.1191
O	5.3377	4.8823	1.2069
O	4.3235	7.2330	-1.8518
O	3.3825	6.8617	0.7911
O	6.3157	5.2315	-1.4416
O	5.8358	7.7589	2.4170
O	7.8928	6.4666	0.9937
O	7.4016	8.1044	-1.2640
O	5.3278	9.4211	0.1795

SAPR-8	Ideal structure		CShM =	0.52003
Sm	M	5.7269	6.9982	0.1278
O	L1	5.3795	4.9315	1.3419
O	L3	4.4700	7.3685	-1.9092
O	L2	3.4058	7.0523	0.8176
O	L4	6.4437	5.2476	-1.3849
O	L6	5.7766	7.6228	2.4673
O	L5	7.9247	6.3468	0.9100
O	L8	7.2816	8.0700	-1.3889
O	L7	5.1336	9.3461	0.1684

	Structure	$Eu(Mo_5)_2$	
Eu	1.9898	10.9730	13.1221
O	2.7701	10.5392	10.9097
O	0.9766	8.7819	12.6694
O	2.2915	13.1128	11.9893
O	0.6168	12.3462	14.5328
O	3.5686	12.3345	14.3586
O	4.0743	9.7809	13.2998
O	-0.1087	11.2995	11.9968
O	1.6979	9.8000	15.1822

SAPR-8	Ideal structure	CShM =	0.63337	
Eu	M	1.9863	10.9965	13.1179
O	L1	2.6068	10.5508	10.8368
O	L5	1.1996	8.7664	12.6769
O	L2	2.1919	13.1608	12.0883
O	L7	0.6129	12.4574	14.4468
O	L3	3.5259	12.2112	14.5109
O	L4	3.9408	9.6013	13.2594
O	L6	-0.0370	11.2833	11.8488
O	L8	1.8496	9.9405	15.2749

	Structure	$Gd(Mo_5)_2$	
Gd	4.4389	10.9674	4.1433
O	4.1526	13.0990	5.2649
O	5.4436	8.7904	4.5979
O	4.7303	9.7978	2.0908
O	5.8075	12.3366	2.7422
O	2.8706	12.3119	2.9063
O	2.3683	9.7731	3.9655
O	3.6433	10.5219	6.3535
O	6.5248	11.2820	5.2666

SAPR-8	Ideal structure	CShM =	0.61095	
Gd	M	4.4422	10.9867	4.1479
O	L1	4.2368	13.1421	5.1725
O	L7	5.2231	8.7655	4.5888
O	L8	4.5823	9.9349	2.0004
O	L5	5.8136	12.4404	2.8275
O	L4	2.9130	12.1972	2.7574
O	L3	2.4954	9.5987	4.0028
O	L2	3.8192	10.5435	6.4179
O	L6	6.4544	11.2711	5.4159

	Structure	$Tb(Mo_5)_2$	
Tb	1.9507	11.0097	13.2916
O	0.9639	8.8271	12.8697
O	2.2424	13.0973	12.1312
O	4.0083	9.8108	13.5207
O	0.5664	12.3977	14.6461
O	3.4935	12.3711	14.5307
O	2.7400	10.5113	11.1144
O	1.6589	9.8874	15.3614
O	-0.1371	11.3029	12.1550

SAPR-8		Ideal structure	CShM =	0.56489
Tb	M	1.9430	11.0239	13.2912
O	L1	1.1697	8.8008	12.8924
O	L7	2.1536	13.1504	12.2270
O	L5	3.8832	9.6458	13.4788
O	L3	0.5655	12.4986	14.5666
O	L8	3.4559	12.2605	14.6627
O	L6	2.5809	10.5357	11.0431
O	L4	1.7885	10.0205	15.4518
O	L2	-0.0533	11.2790	12.0072

	Structure	$Dy(Mo_5)_2$	
Dy	4.4755	11.5432	4.1527
O	4.1918	9.4178	5.2684
O	2.9117	10.2132	2.9237
O	5.4526	13.7186	4.6097
O	6.5446	11.2305	5.2700
O	2.4247	12.7431	3.9859
O	4.7503	12.6944	2.1118
O	3.7236	11.9704	6.3488
O	5.8375	10.1807	2.7676

SAPR-8		Ideal structure	CShM =	0.52472
Dy	M	4.4791	11.5235	4.1598
O	L1	4.2802	9.3815	5.1763
O	L2	2.9555	10.3215	2.7834
O	L8	5.2552	13.7298	4.5969
O	L5	6.4829	11.2411	5.4114
O	L3	2.5440	12.9012	4.0245
O	L7	4.6094	12.5703	2.0272
O	L4	3.8687	11.9613	6.4174
O	L6	5.8371	10.0816	2.8417

Structure		$Ho(MO_5)_2$	
Ho	4.4740	11.5067	4.1427
O	4.1936	9.3959	5.2437
O	5.4425	13.6672	4.5897
O	4.7395	12.6514	2.1106
O	3.7369	11.9307	6.3249
O	2.4413	12.6999	3.9894
O	5.8268	10.1521	2.7611
O	2.9170	10.1831	2.9151
O	6.5375	11.1974	5.2558

SAPR-8	Ideal structure		CShM =	0.50072
Ho	M	4.4788	11.4871	4.1481
O	L1	4.2841	9.3572	5.1574
O	L7	5.2508	13.6801	4.5822
O	L8	4.6028	12.5293	2.0287
O	L2	3.8775	11.9205	6.3940
O	L3	2.5546	12.8562	4.0194
O	L5	5.8257	10.0551	2.8334
O	L4	2.9612	10.2930	2.7828
O	L6	6.4737	11.2059	5.3869

Structure		$Er(MO_5)_2$	
Er	4.4667	11.4998	4.1444
O	4.1840	9.3970	5.2413
O	2.9153	10.1872	2.9261
O	5.4293	13.6538	4.5966
O	6.5126	11.1947	5.2530
O	2.4441	12.6953	3.9918
O	4.7338	12.6279	2.1238
O	3.7306	11.9240	6.3175
O	5.8087	10.1472	2.7759

SAPR-8	Ideal structure		CShM =	0.48727
Er	M	4.4695	11.4808	4.1523
O	L1	4.2717	9.3628	5.1582
O	L2	2.9591	10.2935	2.7935
O	L8	5.2402	13.6619	4.5829
O	L5	6.4527	11.1992	5.3870
O	L3	2.5571	12.8455	4.0211
O	L7	4.5963	12.5155	2.0427
O	L4	3.8697	11.9148	6.3859
O	L6	5.8088	10.0528	2.8468

	Structure	**$Tm(Mo_5)_2$**	
Tm	13.6025	11.0039	12.9676
O	12.0908	12.3277	11.7471
O	13.3270	13.0413	14.1074
O	12.8528	10.4968	15.1069
O	11.6056	9.7989	12.7361
O	15.6431	11.2776	14.0986
O	14.5320	8.8520	13.3779
O	13.8640	9.9246	10.9317
O	14.9608	12.3726	11.6524

SAPR-8		Ideal structure	CShM =	0.43015
Tm	M	13.6087	11.0106	12.9695
O	L1	12.1261	12.2296	11.6332
O	L2	13.4050	13.0891	14.0224
O	L3	12.9855	10.5222	15.1703
O	L4	11.7067	9.6627	12.7810
O	L7	15.5659	11.2530	14.2268
O	L8	14.3650	8.8302	13.3490
O	L5	13.7574	10.0376	10.8479
O	L6	14.9582	12.4604	11.7257

	Structure	**$Tm(Mo_5)_2$**	
Yb	13.5776	11.0089	12.9615
O	12.0687	12.3334	11.7485
O	13.2976	13.0383	14.0975
O	12.8304	10.5038	15.0879
O	11.5865	9.8146	12.7336
O	15.6108	11.2760	14.0834
O	14.5077	8.8606	13.3735
O	13.8411	9.9314	10.9333
O	14.9333	12.3693	11.6556

SAPR-8		Ideal structure	CShM =	0.45106
Yb	M	13.5837	11.0151	12.9639
O	L1	12.1077	12.2308	11.6329
O	L2	13.3820	13.0854	14.0133
O	L3	12.9625	10.5283	15.1559
O	L4	11.6882	9.6737	12.7756
O	L7	15.5334	11.2550	14.2165
O	L8	14.3357	8.8426	13.3413
O	L5	13.7313	10.0464	10.8502
O	L6	14.9290	12.4588	11.7254

Structure	$Lu(Mo_5)_2$		
Lu	13.5998	11.0034	12.9725
O	12.0949	12.3214	11.7670
O	13.3206	13.0258	14.1063
O	12.8578	10.4986	15.0973
O	11.6151	9.8059	12.7505
O	15.6248	11.2678	14.0869
O	14.5196	8.8607	13.3758
O	13.8599	9.9374	10.9462
O	14.9544	12.3643	11.6664

SAPR-8	Ideal structure		CShM =	0.43115
Lu	M	13.6052	11.0095	12.9743
O	L1	12.1313	12.2233	11.6509
O	L2	13.4056	13.0726	14.0236
O	L3	12.9892	10.5213	15.1605
O	L4	11.7148	9.6720	12.7878
O	L7	15.5513	11.2472	14.2207
O	L8	14.3558	8.8426	13.3468
O	L5	13.7492	10.0461	10.8652
O	L6	14.9447	12.4507	11.7390

Structure	$(TBA)_4[Ba\{Mo_5O_{13}(OMe)_4NO\}_2]$		
Ba	-0.0939	9.0910	7.7964
O	-2.5082	7.8437	7.4175
O	-0.4759	7.0164	9.5787
O	-0.3162	7.8164	5.3374
O	1.7010	7.0092	7.4643
O	2.3204	10.3383	8.1753
O	0.2881	11.1656	6.0142
O	0.1285	10.3656	10.2554
O	-1.8888	11.1728	8.1286

CU-8	Ideal structure		CShM =	0.39490
Ba	M	-0.0939	9.0910	7.7964
O	L1	-2.5960	7.9783	7.4703
O	L2	-0.4816	7.1283	9.6945
O	L4	-0.2869	7.9849	5.2776
O	L3	1.8275	7.1349	7.5018
O	L7	2.4082	10.2037	8.1225
O	L8	0.2938	11.0537	5.8984
O	L6	0.0991	10.1971	10.3153
O	L5	-2.0153	11.0471	8.0911

SAPR-8	Ideal structure	CShM =	11.07178	
Ba	M	-0.0939	9.0910	7.7964
O	L1	-2.0056	7.3224	7.7105
O	L2	0.1579	7.7094	9.9914
O	L5	-0.6578	8.8206	5.2668
O	L6	1.0643	6.8713	7.0744
O	L7	2.4019	9.3679	8.4925
O	L8	0.6798	11.3172	6.6849
O	L3	-0.1141	10.8531	9.7159
O	L4	-2.2775	10.4661	7.4351

Structure	**$Sr(Mo_5)_2$**		
Sr	9.4344	8.5885	15.2608
O	7.6895	10.5047	15.5245
O	9.8532	10.5177	13.5663
O	11.7297	9.6905	15.6866
O	9.5647	9.6855	17.6268
O	11.1765	6.6596	14.9643
O	9.0746	6.6901	17.0167
O	7.1095	7.4668	14.9356
O	9.2104	7.4274	12.9137

CU-8	Ideal structure	CShM =	0.15332	
Sr	M	9.4269	8.5812	15.2772
O	L1	7.6156	10.4353	15.5365
O	L2	9.8233	10.4378	13.4935
O	L3	11.7903	9.6225	15.6181
O	L4	9.5826	9.6199	17.6611
O	L7	11.2383	6.7271	15.0180
O	L8	9.0306	6.7246	17.0610
O	L5	7.0636	7.5399	14.9364
O	L6	9.2713	7.5425	12.8934

SAPR-8	Ideal structure	CShM =	10.57529	
Sr	M	9.4269	8.5812	15.2772
O	L1	7.3688	9.8872	14.9086
O	L5	10.1777	10.6862	14.2367
O	L8	11.7797	8.8373	15.9674
O	L4	9.3875	10.2474	17.0938
O	L7	10.5269	6.4790	14.6076
O	L3	9.6344	7.2724	17.3561
O	L2	7.6157	6.9122	15.1708
O	L6	8.9249	8.3279	12.8770

Structure	$(TBA)_2[U\{Mo_5O_{13}(OMe)_4NO\}_2]$		
U	16.0425	5.8249	5.3506
O	15.0611	4.4527	6.9719
O	14.3721	4.5566	4.3002
O	14.5190	7.3322	4.3677
O	15.2241	7.2252	7.0659
O	17.2463	3.8499	4.9169
O	16.8319	5.9158	3.1366
O	17.3881	7.7631	5.1265
O	17.8164	5.6717	6.9326

SAPR-8	Ideal structure		CShM =	0.26873
U	M	16.0557	5.8436	5.3521
O	L1	15.1818	4.3843	6.9800
O	L2	14.4695	4.5294	4.2119
O	L3	14.6014	7.3859	4.3277
O	L4	15.3138	7.2408	7.0958
O	L6	17.1265	3.7821	4.9685
O	L7	16.7161	5.9046	3.0930
O	L8	17.3131	7.8219	5.1322
O	L5	17.7235	5.6994	7.0077

Structure	$(TBA)_2[Th\{Mo_5O_{13}(OMe)_4NO\}_2]$		
Th	13.5103	4.8718	14.8179
O	13.2702	5.5375	12.4994
O	14.0462	7.2175	14.6296
O	13.6725	2.7820	13.6354
O	12.0323	6.1112	16.2493
O	11.2601	4.4224	14.1171
O	12.8851	3.1033	16.3349
O	15.7631	4.6285	14.0666
O	14.9902	4.9519	16.7538

SAPR-8	Ideal structure		CShM =	0.41847
Th	M	13.4922	4.8473	14.7893
O	L1	13.3476	5.4671	12.4701
O	L2	14.2218	7.1374	14.7044
O	L5	13.6390	2.7764	13.5753
O	L3	12.1070	6.0072	16.3767
O	L4	11.2328	4.3369	14.1424
O	L8	12.7618	3.1583	16.3377
O	L6	15.7525	4.7567	13.9728
O	L7	14.8753	5.1386	16.7352

Structure	$(TBA)_2[Np\{Mo_5O_{13}(OMe)_4NO\}_2]$		
Np	5.1419	1.6890	5.3433
O	3.4941	2.9544	4.2730
O	3.6454	0.1775	4.3518
O	4.3345	0.3009	7.0442
O	4.1843	3.0658	6.9509
O	5.9127	1.6051	3.1281
O	6.4792	-0.2422	5.1319
O	6.8936	1.8383	6.9379
O	6.3286	3.6660	4.9261

SAPR-8		Ideal structure	CShM =	0.19369
Np	M	5.1571	1.6727	5.3430
O	L1	3.5813	2.9794	4.2018
O	L2	3.7142	0.1360	4.3187
O	L3	4.4133	0.2820	7.0767
O	L4	4.2804	3.1254	6.9598
O	L6	5.8227	1.6115	3.0967
O	L7	6.4110	-0.2958	5.1295
O	L8	6.8113	1.8181	6.9970
O	L5	6.2230	3.7254	4.9642

Structure	$(TBA)_2[Zr\{Mo_5O_{13}(OMe)_4NO\}_2]$		
Zr	4.5027	5.8055	18.7716
O	3.2560	7.6149	18.9883
O	3.8235	5.8707	20.8634
O	3.4253	3.9259	19.1631
O	2.8557	5.6563	17.2770
O	5.3824	4.4959	17.2664
O	5.2297	7.1055	17.1665
O	5.8944	7.2350	19.7082
O	6.0407	4.6209	19.7928

SAPR-8		Ideal structure	CShM =	0.11600
Zr	M	4.4901	5.8145	18.7775
O	L1	3.3054	7.6560	18.9854
O	L2	3.8799	5.8697	20.8900
O	L3	3.5021	3.8832	19.1408
O	L4	2.9275	5.6696	17.2362
O	L8	5.3092	4.4548	17.2550
O	L5	5.1701	7.1225	17.1451
O	L6	5.8435	7.2640	19.7287
O	L7	5.9826	4.5963	19.8386

Structure	(TBA)2[Hf{Mo5O13(OMe)4NO}2]		
Hf	4.5093	5.8066	18.7801
O	3.2750	7.6082	18.9972
O	3.8362	5.8750	20.8653
O	3.4340	3.9403	19.1698
O	2.8685	5.6596	17.2940
O	5.3840	4.4972	17.2771
O	5.2313	7.1009	17.1815
O	5.8924	7.2318	19.7082
O	6.0415	4.6255	19.7973

SAPR-8	Ideal structure		CShM =	0.11308
Hf	M	4.4969	5.8161	18.7856
O	L1	3.3197	7.6510	18.9929
O	L2	3.8919	5.8729	20.8894
O	L3	3.5116	3.8946	19.1495
O	L4	2.9394	5.6727	17.2529
O	L8	5.3093	4.4606	17.2697
O	L5	5.1736	7.1168	17.1590
O	L6	5.8471	7.2583	19.7304
O	L7	5.9828	4.6022	19.8411

S8. IR Spectra

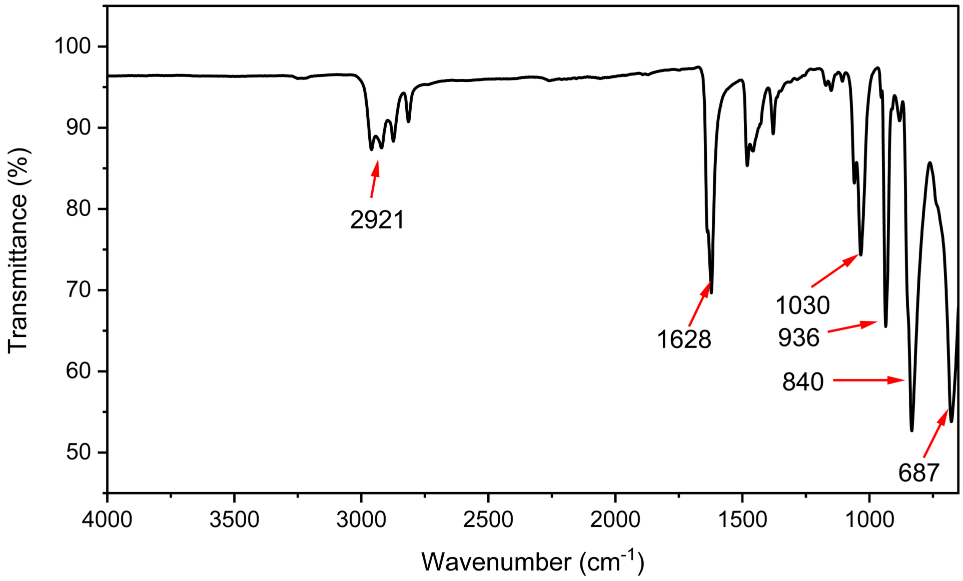

Figure S60. FT-IR spectrum (ATR, 4000–600 cm^{-1}) of **La(Mo$_5$)$_2$**, recorded at room temperature on the pristine compound.

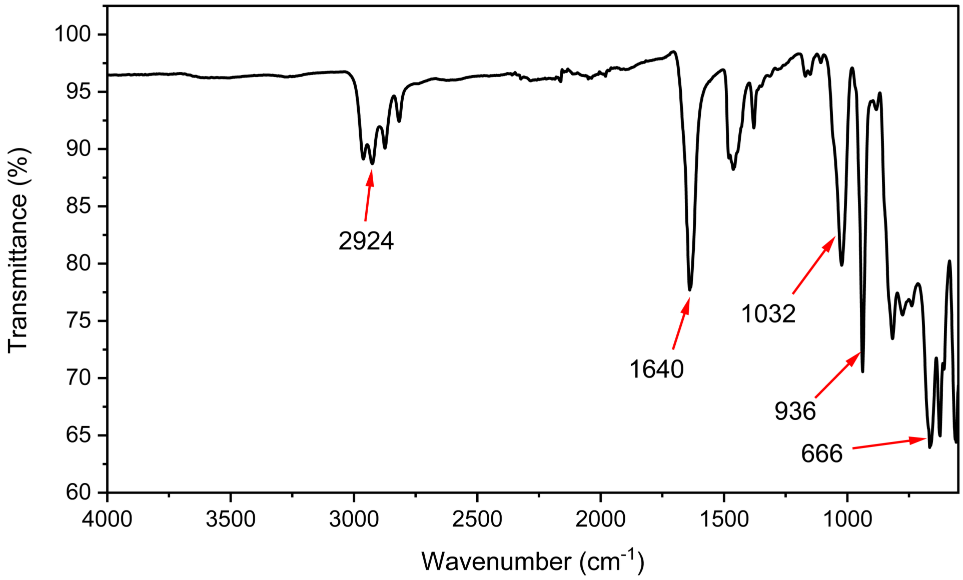

Figure S61. FT-IR spectrum (ATR, 4000–600 cm^{-1}) of **Ce(Mo$_5$)$_2$**, recorded at room temperature on the pristine compound.

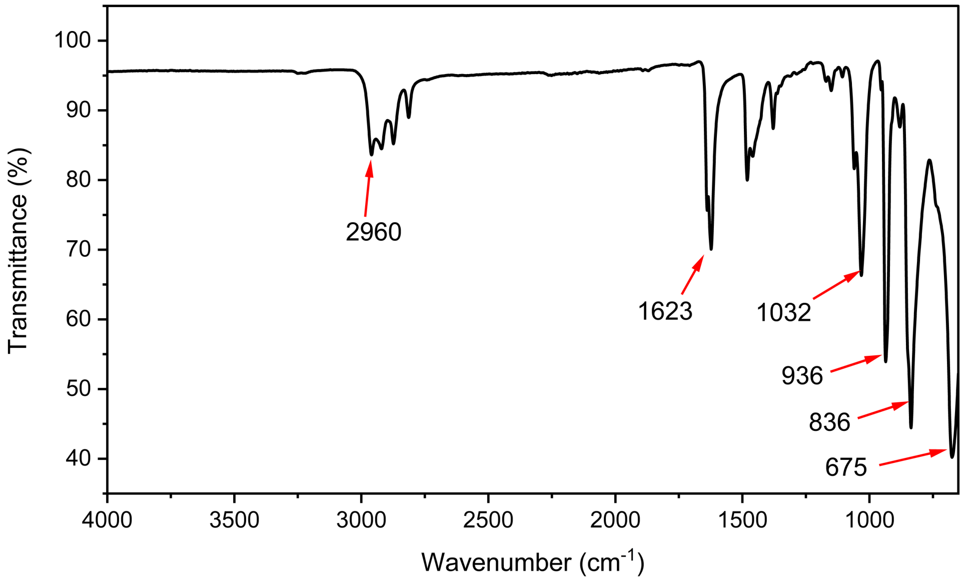

Figure S62. FT-IR spectrum (ATR, 4000–600 cm^{-1}) of **Pr(Mo$_5$)$_2$**, recorded at room temperature on the pristine compound.

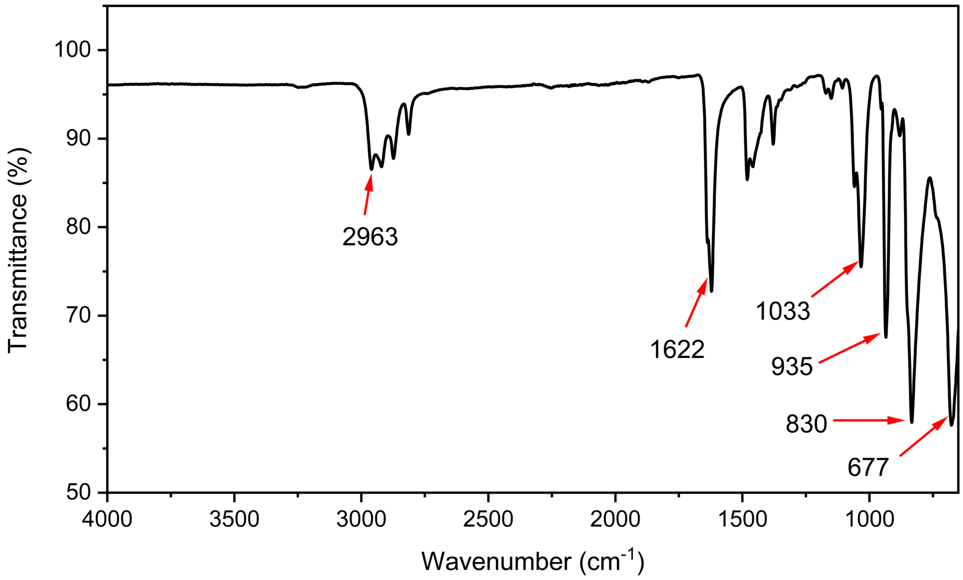

Figure S63. FT-IR spectrum (ATR, 4000–600 cm⁻¹) of **Nd(Mo₅)₂**, recorded at room temperature on the pristine compound.

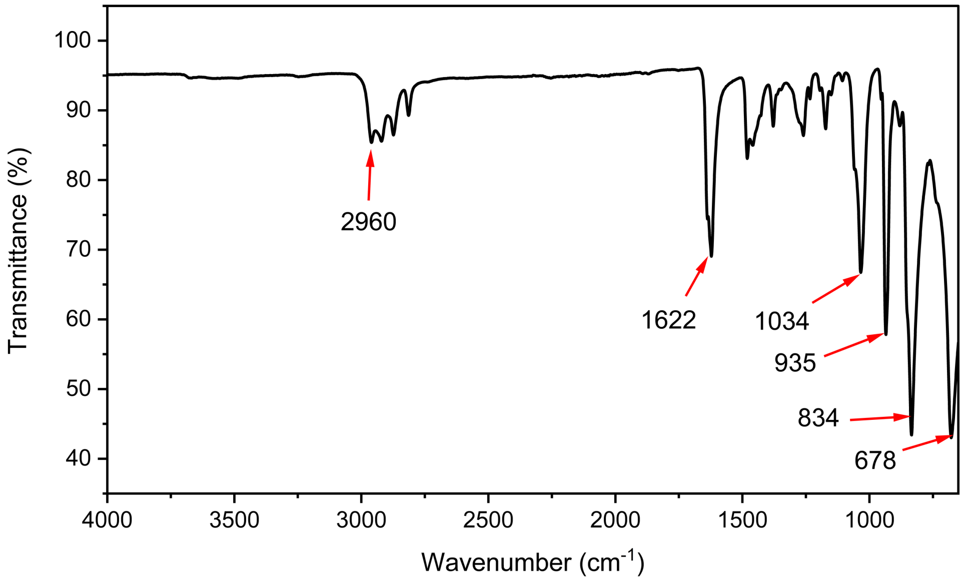

Figure S64. FT-IR spectrum (ATR, 4000–600 cm⁻¹) of **Sm(Mo₅)₂**, recorded at room temperature on the pristine compound.

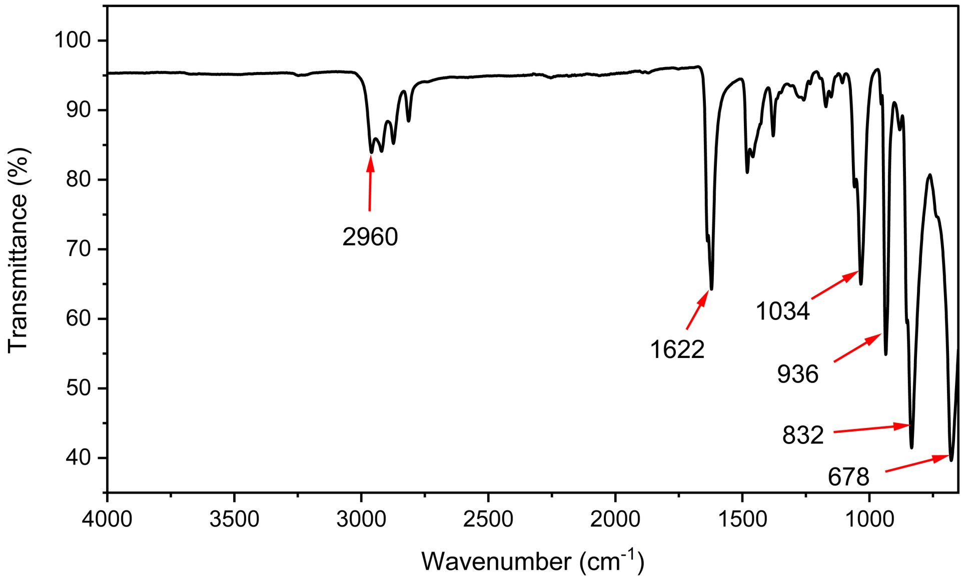

Figure S65. FT-IR spectrum (ATR, 4000–600 cm^{-1}) of **Eu(Mo$_5$)$_2$**, recorded at room temperature on the pristine compound.

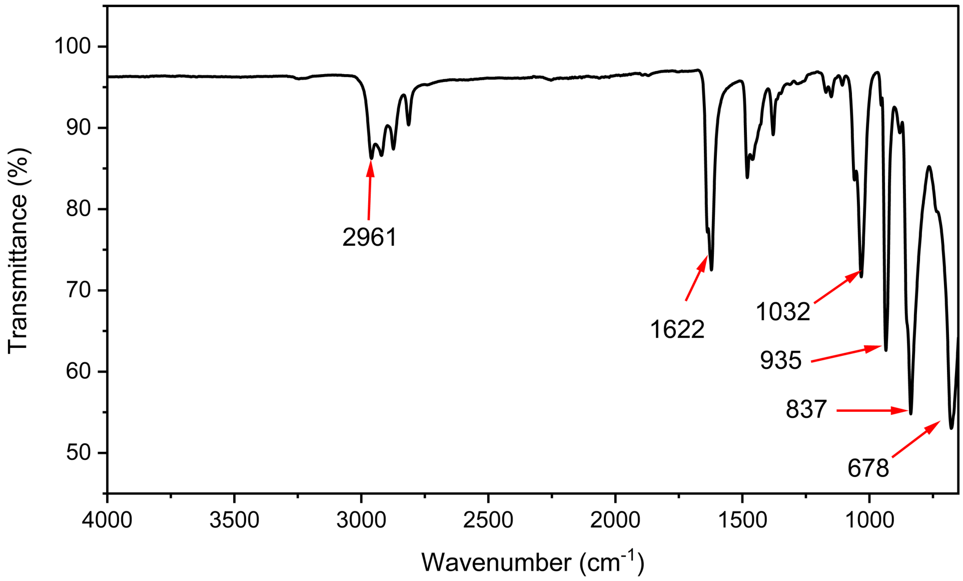

Figure S66. FT-IR spectrum (ATR, 4000–600 cm^{-1}) of **Gd(Mo$_5$)$_2$**, recorded at room temperature on the pristine compound.

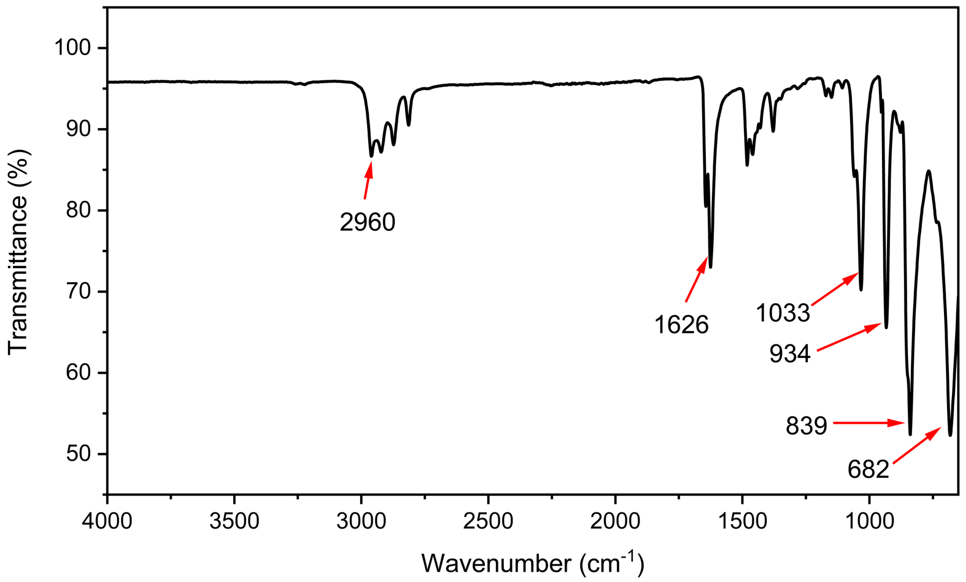

Figure S67. FT-IR spectrum (ATR, 4000–600 cm^{-1}) of **Tb(Mo$_5$)$_2$**, recorded at room temperature on the pristine compound.

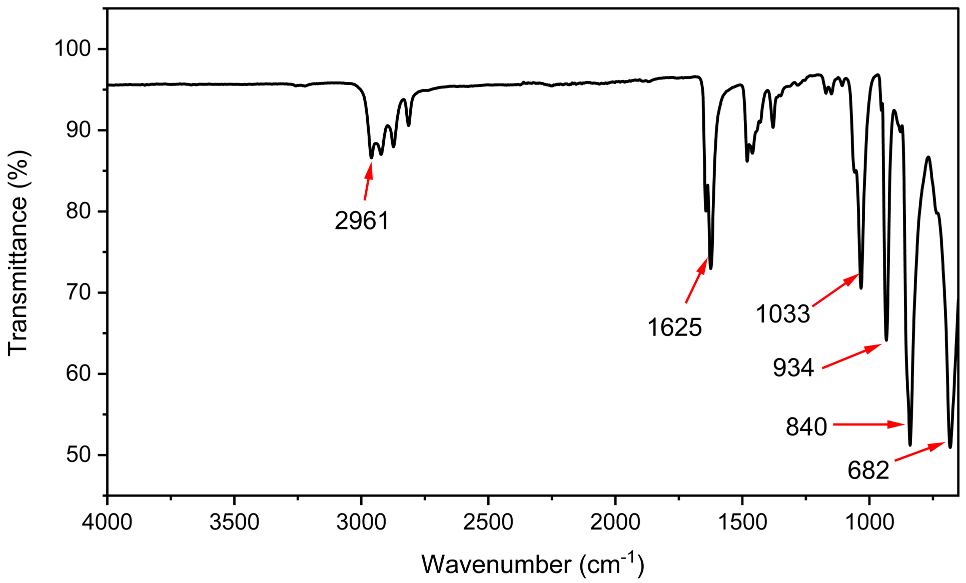

Figure S68. FT-IR spectrum (ATR, 4000–600 cm^{-1}) of **Dy(Mo$_5$)$_2$**, recorded at room temperature on the pristine compound.

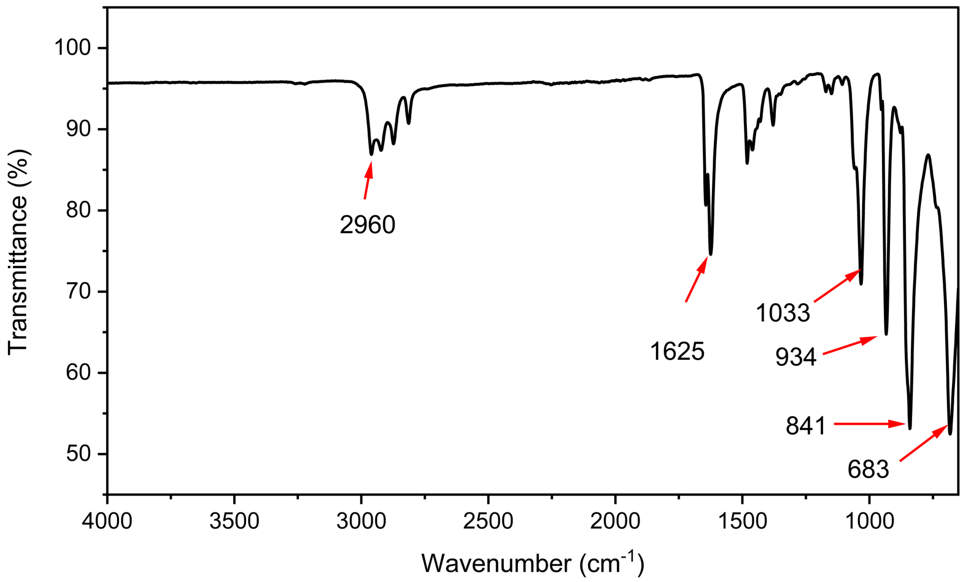

Figure S69. FT-IR spectrum (ATR, 4000–600 cm^{-1}) of **Ho(Mo$_5$)$_2$**, recorded at room temperature on the pristine compound.

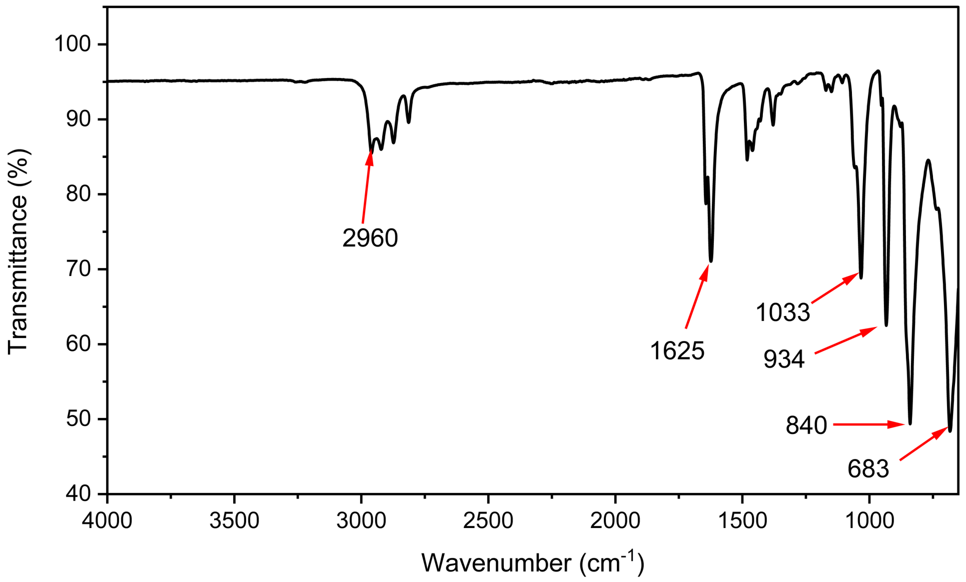

Figure S70. FT-IR spectrum (ATR, 4000–600 cm^{-1}) of **Er(Mo$_5$)$_2$**, recorded at room temperature on the pristine compound.

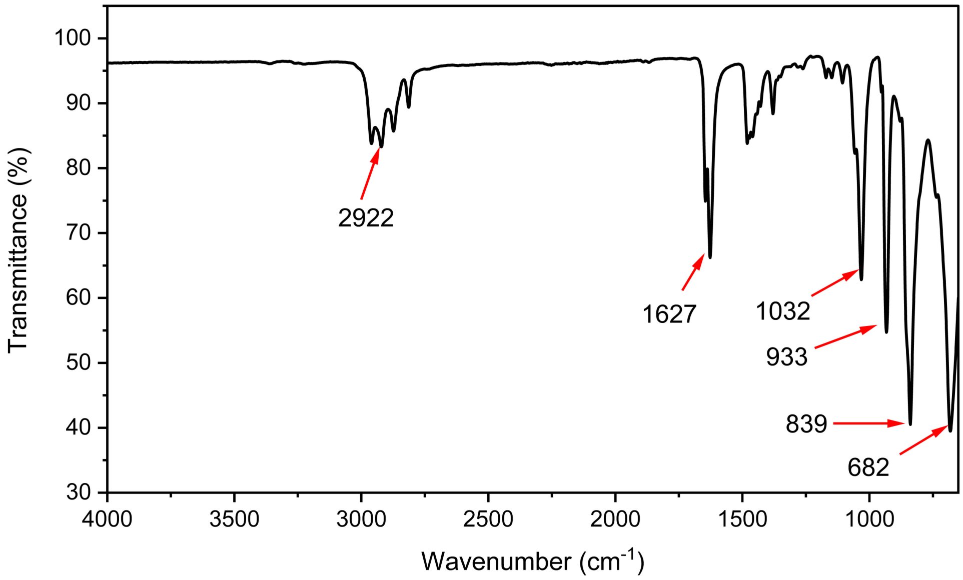

Figure S71. FT-IR spectrum (ATR, 4000–600 cm^{-1}) of **Tm(Mo$_5$)$_2$**, recorded at room temperature on the pristine compound.

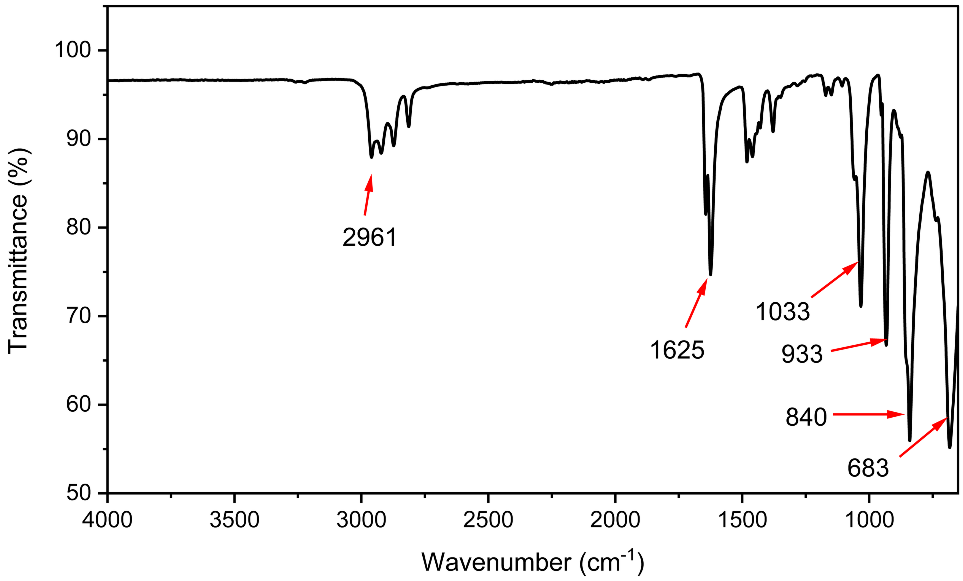

Figure S72. FT-IR spectrum (ATR, 4000–600 cm^{-1}) of **Yb(Mo$_5$)$_2$**, recorded at room temperature on the pristine compound.

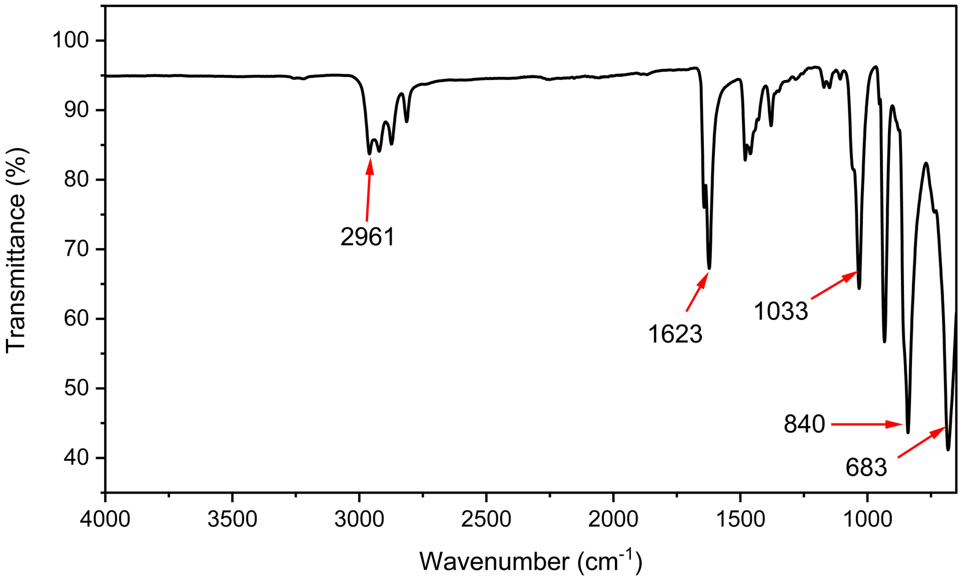

Figure S73. FT-IR spectrum (ATR, 4000–600 cm^{-1}) of **Lu(Mo$_5$)$_2$**, recorded at room temperature on the pristine compound.

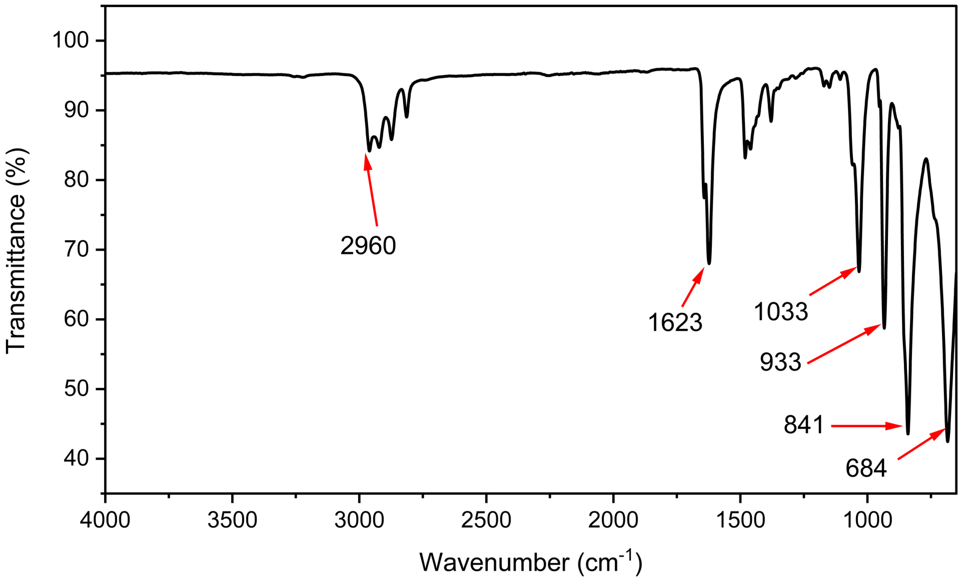

Figure S74. FT-IR spectrum (ATR, 4000–600 cm^{-1}) of **Y(Mo$_5$)$_2$**, recorded at room temperature on the pristine compound.
